# ATP-dependent conformational dynamics in a photoactivated adenylate cyclase revealed by fluorescence spectroscopy and small-angle X-ray scattering

**DOI:** 10.1038/s42003-024-05842-1

**Published:** 2024-02-02

**Authors:** K. Ujfalusi-Pozsonyi, E. Bódis, M. Nyitrai, A. Kengyel, E. Telek, I. Pécsi, Z. Fekete, N. Varnyuné Kis-Bicskei, C. Mas, D. Moussaoui, P. Pernot, M. D. Tully, M. Weik, G. Schirò, S. M. Kapetanaki, A. Lukács

**Affiliations:** 1 Department of Biophysics, Medical School, University of Pécs, 7624 Pécs, Hungary; 2 Univ. Grenoble Alpes, CNRS, CEA, EMBL, ISBG, F-38000 Grenoble, France; 3 European Synchrotron Radiation Facility (ESRF), Grenoble, France; 4 Institut de Biologie Structurale (IBS), Université Grenoble Alpes, CEA, CNRS, Grenoble, France; 5 Present Address: Department of Biophysics, Medical School, University of Pécs, 7624 Pécs, Hungary

**Keywords:** Molecular biophysics, SAXS

## Abstract

Structural insights into the photoactivated adenylate cyclases can be used to develop new ways of controlling cellular cyclic adenosine monophosphate (cAMP) levels for optogenetic and other applications. In this work, we use an integrative approach that combines biophysical and structural biology methods to provide insight on the interaction of adenosine triphosphate (ATP) with the dark-adapted state of the photoactivated adenylate cyclase from the cyanobacterium *Oscillatoria acuminata* (OaPAC). A moderate affinity of the nucleotide for the enzyme was calculated and the thermodynamic parameters of the interaction have been obtained. Stopped-flow fluorescence spectroscopy and small-angle solution scattering have revealed significant conformational changes in the enzyme, presumably in the adenylate cyclase (AC) domain during the allosteric mechanism of ATP binding to OaPAC with small and large-scale movements observed to the best of our knowledge for the first time in the enzyme in solution upon ATP binding. These results are in line with previously reported drastic conformational changes taking place in several class III AC domains upon nucleotide binding.

## Abstract (web summary)

Small and large-scale movements are observed in the dark-adapted state of a photoactivated adenylate cyclase from the cyanobacterium *Oscillatoria acuminata* upon ATP binding using fluorescence and solution X-ray scattering methods.

## Introduction

Adenosine 3’,5’-cyclic monophosphate (cAMP) is a universal regulator of metabolism and gene expression in all life forms^1^. cAMP-producing enzymes are traditionally termed adenylate cyclases (ACs)^2–4^. Coupling of enzymatic domains with photoreceptor protein domains forms powerful light-regulated biocatalysts utilized in optogenetics^5,6^. Hence, photoactivated adenylate cyclases (PACs) have emerged as promising tools in the field of optogenetics for the fine-tuning of intracellular cAMP levels in heterologous cell systems^7,8^.

In the photosynthetic cyanobacterium *Oscillatoria acuminata* the formation of cAMP from ATP is catalysed by the photoactivated adenylate cyclase OaPAC, a recently discovered flavoprotein that translates a blue-light signal into the production of cAMP^9^. OaPAC is a homodimer of a 366-aa residue protein comprising of an N-terminal blue-light using flavin (BLUF) domain and a C-terminal class III adenylyl cyclase (AC) domain. Blue-light absorption by the flavin is characterized by a rearrangement of the hydrogen bond network as reflected by the red-shift of 10 nm of the S_0_→S_1_ transition^10^. The AC activity of OaPAC has been reported to be stimulated by light up to 20-fold^9,11^ more than basal levels in the dark, whereas the following *k*_cat_, *K*_M_, and *k*_cat_/*K*_M_ values of 205 ± 11 min^−1^, 0.12 ± 0.01 mM, and 1888 mM^−1^ min^−1^, respectively have been estimated for the light-dependent conversion of ATP to cAMP^12^. Three-dimensional X-ray crystal structures of OaPAC both in the dark- and light-adapted states have been solved. A non-hydrolyzable ATP analogue has been co-crystallized with OaPAC, yet no electron density for the analogue has been observed^9,10^. Recently, ATP-bound dark-state structures of OaPAC have been solved by cryo-crystallography at a synchrotron and by room temperature serial femtosecond crystallography (SFX^13^) at an X-ray free electron laser^14^. The former revealed a non-productive ATP binding mode in the AC domain and the latter a second ATP conformation with 50% occupancy and differing in the orientation of the ribose group and the position of the adenine. For PAC from *Beggiatoa sp*. (bPAC), which is homologous to OaPAC (~57% sequence identity), the structure has been solved for a Y7F mutant in a pseudo-lit state in complex with adenosine-5’-(α-thio)-triphosphate (ATP-S(Rp))^15^. In that homologue, a canonical type III fold characterizes the AC domains which face each other in an antiparallel arrangement and form two active sites at the dimer interface with an AC opening angle of 32.8° that is smaller than the >36° angle observed in other AC structures bound to other nucleotides^15^. Molecular dynamics simulations suggested that ATP binding has significant effects on the structure and flexibility of adenylate cyclases^16^.

In this work, we provide a detailed experimental characterization of the structural and dynamic changes induced by ATP-binding to OaPAC in solution, and discuss the energetics that define binding specificity. We demonstrate that ATP binding results in a significant expansion of OaPAC. We further identify thermodynamic parameters as well as the binding affinity and the binding kinetics of the ATP binding to OaPAC. Our study provides the first in-solution biophysical characterization of the complex of OaPAC with its natural substrate ATP.

## Methods

### Expression and purification of full-length OaPAC

Full-length OaPAC was expressed and purified as described previously^12^. The pCold-I-OaPAC plasmid was transformed into BL21(DE3) *E. coli* and grown on an LB-agar plate containing 100 µg/mL ampicillin. A single colony was used to inoculate 10 mL of Luria Broth (LB) medium containing 100 µg/ml ampicillin. The starting culture was used to inoculate 1 L of LB/ampicillin medium in a 4-L flask. The 1-L culture was incubated at 37 °C until the optical density 600 (OD_600_) reached 0.4-0.5, after which the temperature was decreased to 18 °C followed by the addition of 1 mM isopropyl-β-D-1-thiogalactopyranoside (IPTG) to induce protein expression. The cells were harvested after overnight incubation by centrifugation (6000 rcf, 4°C) and the cell pellet was stored at −20 °C. The cell pellet was resuspended in lysis buffer containing protease cocktail inhibitor, DNAse I (20 units/ml), lysozyme (0.5 mg/ml) and *β*-mercaptoethanol (0.15 μl/ml). The resuspended cells were lysed by sonication and the cell debris was removed by centrifugation (39,000 rcf, 30 min). The supernatant was loaded onto a Ni-NTA (Qiagen) column, which was washed with a buffer containing 20 mM imidazole, and the protein was eluted with a wash buffer using 300 mM imidazole. The fractions containing the protein were pooled together and purified to homogeneity using size-exclusion chromatography (Superdex-200) and the purity was assessed by SDS-PAGE. The protein concentration was determined using the extinction coefficients ε_445nm_ = 11,300 M^−1^cm^−1^ and ε_280nm_ = 28,590 M^−1^cm^−1^. The following buffers were used: lysis/wash buffer (50 mM Tris pH 8.00, 300 mM NaCl, 20 mM imidazole), elution buffer (50 mM Tris pH 8.00, 300 mM NaCl, 300 mM imidazole) and gel-filtration buffer (50 mM Tris pH 8.00, 150 mM NaCl).

### Mass photometry

Mass photometry^17^ experiments were performed in duplicates using a Reyfen OneMP (Reyfen Ltd. Oxford, UK) MP system. Movies of single molecules of dimeric OaPAC and buffer were recorded with the AcquireMP software whereas data analysis was carried out using the DiscoverMP software (Supplementary Movies 1 and 2). Clean microscope coverslips (high precision glass coverslips Marienfeld) were used. The sample droplet was kept in shape with the help of self-adhesive silicon culture wells (Grace Bio-Labs reusable CultureWell^TM^ gaskets). The focal point was identified and secured in place with an autofocus system based on total internal reflection for the entire measurement. A mix of proteins with molecular weight 66, 146, 480 and 1048 kDa was used to achieve a contrast-to-mass calibration. Sample drops were formed by adding 1–2 μl of OaPAC solution into 18–19 μl of buffer, to reach a drop volume of 20 μl and a final protein concentration of ~100 nM. OaPAC was dissolved in 50 mM Tris pH 8.00, 150 mM NaCl, 2 mM MgCl_2_. The mass photometry movie was recorded at 1 kHz, with an exposure time for a single frame 0.95 ms. 5994 frames were collected in 60 s.

### Small-angle X-ray scattering (SAXS) data collection and analysis

OaPAC was dissolved in 50 mM Tris buffer pH 8.5, 150 mM NaCl, 1 mM MgCl_2_ buffer and spun down at room temperature before the SAXS measurements. Experiments were performed in duplicates in batch-mode at the BM29 beamline at the European Synchrotron Radiation Facility (ESRF)^18^ (Supplementary Table 1). Scattered X-rays at a wavelength of 0.992 Å (corresponding to an energy of 12.5 keV) were recorded with a PILATUS3 X 2 M (Dectris) detector in the SAXS region (*q* = 0.02–0.5 Å^−1^). Data were acquired for OaPAC at various concentrations of the enzyme (1, 2, 5, 10 mg/ml), at various ATP concentrations (10, 50 70, 100, 150, 200, 250, 300, 400, 500, 700, 800, and 1000 μM) and in the presence of the ligands ApCpp (Adenosine-5’-[(α,β)-methyleno]triphosphate, non-hydrolyzable ATP analogue) (1 mM) and cAMP (1 mM). For each sample measurement, ten X-ray scattering measurements of 1 second exposure were collected with ten analogous signals of the buffer before and after each sample measurement. Each two-dimensional pattern was converted to a one-dimensional scattering pattern by azimuthal integration using the upstream data reduction pipeline available at the BM29 beamline^18,19^. The scattering patterns were averaged and protein signals were obtained by subtraction of the corresponding buffer using BioXTAS RAW^20,21^ (Supplementary Table 2). The radius of gyration *R*_g_ was estimated by the Guinier approximation. The real space interatomic distance distribution function P(r), was obtained by indirect Fourier transformation of the scattering data by using the program GNOM^22^ of the RAW software. Kratky plots were also generated with RAW and the graphs were plotted using OriginPro 2022b (OriginLab). Porod volumes (Vp) and an independent calculation of the molecular weight were also estimated with RAW. Quantification of the structural transitions occurring upon ATP binding to OaPAC was obtained by the estimation of the apparent equilibrium constants for the Rg and Dmax (*K*_Rg_,_app_, *K*_Dmax_,_app_) and those derived from the Kratky analysis (*K*_q=0.05_, and *K*_q=0.11_)^23^. Theoretical scattering curves were calculated from the crystal structures of OaPAC (pdb: 4yus and 4yut) using FoXS^24,25^ and CRYSOL^26^. The dimeric model of 4yus was generated using PyMOL (Schrödinger, New York, USA). 4yus has been solved at 1.8 Å resolution with ApCpp added whereas 4yut has been solved at 2.9 Å without the addition of any nucleotide. A low-resolution electron density model was generated by the DENSS^27^ algorithm in the RAW software. The model was visualized in PyMOL and was superimposed with the model obtained from the crystal structure. In addition, SEC (size-exclusion chromatography)-SAXS (online SAXS)^28^ experiments were performed at BM29 with a Superdex 200 increase 10/300 column (GE Healthcare) and purified OaPAC (20 mg/ml). SEC-SAXS data were analyzed with CHROMIXS^29^. All measurements were performed under red-light conditions to ensure that OaPAC is present in the dark-adapted state.

### Isothermal calorimetry

Isothermal titration calorimetry^30^ (ITC) assays were employed using a PEAQ-ITC micro-calorimeter (Malvern Panalytical). Purified OaPAC (50 μΜ) was dialyzed in 50 mM Tris pH 8.0, 150 mM NaCl, 5 mM MgCl_2_. Solutions of ATP (800 μM), ApCpp (3 mM) and cAMP (5 mM) were prepared in the same buffer immediately prior to the titration. OaPAC was placed in the cell and the nucleotide in the syringe. Titrations were performed in duplicates at 25 °C with 1 injection of 1 μl followed by 15 injections of 2.5 μl each, separated by 3 min, into the 200 μl sample cell. Continuous stirring was performed at 700 rpm. Data were fitted to one-set-of-sites model using the PEAQ-ITC software (Malvern). Each binding isotherm was measured in duplicate. Control experiments were performed by titrating each one of the nucleotides in the same buffer. The change in enthalpy (Δ*H*), free energy (Δ*G*), and entropy (Δ*S*) were determined using the manufacturer’s MALVERN PEAQ-ITC analysis software.

### Thermal denaturation assays

Determination of the melting temperature of OaPAC and its complexes with the nucleotides was accomplished using the following biophysical methods:

i. Miniaturized differential scanning fluorimetry (nanoDSF)^31^ was applied using the Prometheus NT.48 device (Nanotemper) controlled by PR. ThermControl (version 2.1.2). Standard-grade glass capillaries were filled with 10–15 μl OaPAC (1.35 mg/ml) and its complexes with ATP (1 mM) and ApCpp (1 mM) and placed onto the capillary tray of the instrument. OaPAC was dissolved in 50 mM Tris pH 8.0, 150 mM NaCl, 1 mM MgCl_2_. Excitation power was pre-adjusted to get fluorescence readings above 2000 arbitrary units for the emission wavelengths at 330 nm (F330) and 350 nm (F350). Samples were measured in duplicates in the temperature range 20–95 °C with a temperature slope of 1 °C/min. An XLSX file with ‘processed data’ was exported from PR and OriginPro 2022b and was used for further analysis.
ii. Differential scanning calorimetry (DSC)^32^ measurements were done using a Setaram Micro DSC-III calorimeter. Conventional Hastelloy batch vessels were used with 800 μl sample volume. The DSC measurements were performed in duplicates in the 20–100 °C temperature range, using a 0.3 K/min heating rate. The sample and reference vessels were equilibrated with a precision of 10^−5^ g and a second thermal scan of the denatured OaPAC samples was measured for baseline correction. OaPAC (400 μM) was dissolved in 50 mM Tris pH 8.0, 150 mM NaCl, 2 mM MgCl_2_ and ApCpp dissolved in the same buffer was added in a 1:5 ratio (final concentration 2 mM). The thermal unfolding curve was not measured for the OaPAC-ATP complex as ATP due to aggregation over the course of the measurement (~12 h). The heat flow measured in mW was plotted against temperature. The data were analyzed with OriginPro 2022b, melting temperatures (*T*_m_) were determined from the maximum of the thermal transition and the enthalpy (Δ*H*^cal^) from the area of the thermal unfolding curves.
iii. Temperature-dependent fluorescence emission measurements of OaPAC and its complex with ApCpp were carried out using a Horiba Jobin Yvon Fluorolog 3.22 spectrofluorimeter. The temperature range spanned from 15 to 85 °C using a Quantum Northwest temperature control and the emission spectra were recorded in 5 °C increments with a ramping rate of 1 °C min^−1^. OaPAC was dissolved in 50 mM Tris pH 8.0, 150 mM NaCl and 2 mM MgCl_2_ and ApCpp was added to OaPAC (30 μM) in a 5-fold molar excess. The excitation was set at 360 nm and the emission was detected between 420–650 nm, using 5 nm slits. The emission maximum was plotted against the temperature and the data were fit using a Boltzmann equation (Eq. 1) assuming a two-state transition:
  1 $$F=\frac{{F}_{\min }-{F}_{\max }}{1+{e}^{\left(T-{T}_{m}\right)/m}}+{F}_{\max }$$

where *F* = fluorescence, *F*_min_ is the minimum fluorescence, *F*_max_ is the maximum fluorescence, *T*_m_ is the mid-point of the curve (melting point), m is the slope, and *T* is the temperature at which the fluorescence was measured. The *T*_m_ can also be easily identified as the lowest part of the curve of the first derivative of the fluorescence emission as a function of temperature. The thermal denaturation curve was truncated to remove post-peak quenching. The denaturing agent GuHCl (guanidinium chloride) was also used to chemically unfold OaPAC (30 μM) and emission spectra were recorded at various GuHCl concentrations (0–3 M). The emission maxima were plotted against [GuHCl]. Data were analyzed using OriginPro 2022b.

### Fluorescence anisotropy-based nucleotide-binding assays

Fluorescence anisotropy-based^33^ nucleotide-binding assays were performed in duplicates using 2 μM mantATP, a fluorescent analogue of ATP which, upon excitation at 350 nm emits at ~450 nm. Measurements were conducted at room temperature (RT) in 50 mM Tris pH 8.0, 150 mM NaCl, 2 mM MgCl_2_. Steady-state fluorescence anisotropy measurements were performed with a Fluorolog Jobin Yvon Horiba spectrofluorometer in L-format configuration equipped with a polarization accessory. The measurements were performed in triplicate at an excitation wavelength of *λ*_exc_ = 350 nm with a vertical polarization filter and by measuring the emission at 450 nm (30 measurements) with the polarization filter both parallel and perpendicular with respect to the excitation light polarization. Fluorescence anisotropies were calculated from the fluorescence intensities detected according to Eq. (2)

2 $$r=\frac{{I}_{v}-2G(\lambda ){I}_{h}}{{I}_{v}+{2G(\lambda )I}_{h}}$$

where *r* is the fluorescence anisotropy, *I*_*v*_ is the fluorescence emission intensity detected with vertical polarization, *I*_*h*_ is the fluorescence emission intensity detected with horizontal polarization, and G(λ) is the correction factor experimentally determined measuring the ratio Iv/Ih with a horizontally polarized excitation. Data processing was done using OriginPro 2022b and *K*_D_ values were determined by fitting to a quadratic binding Eq. (3)

3 $$\frac{r-{rA}}{{rAP}-{rA}} = \frac{{A}_{o}+{P}_{o}+{K}_{D} - \sqrt{({{A}_{o}+{P}_{o}+{K}_{D}})^{2}-4* {A}_{o}* {P}_{o}}}{2}$$

where *A*_*o*_ and *P*_*o*_ are the total mantATP and OaPAC concentrations, respectively, rA is the steady-state anisotropy of mantATP, rAP is the steady-state anisotropy of mantATP at a saturating amount of OaPAC and *K*_D_ is the dissociation equilibrium constant of the mantATP-OaPAC complex.

### Stopped flow kinetics measurement

Transient kinetic experiments were performed with an Applied Photophysics SX18 stopped flow apparatus at 20 °C. mantATP and OaPAC dissolved in 50 mM Tris pH 8.0, 150 mM NaCl, 2 mM MgCl_2_ were mixed in a 20 μl flow cell in a 1:1 ratio using two identical 2.5 ml syringes. mantATP was excited at 350 nm and its emission was monitored at ~450 nm using a band-pass filter, FS450 in order to exclude the fluorescence of the flavin, which appears at higher wavelengths (~520 nm, see the temperature-dependent fluorescence measurements). The dead time of the instrument was 1.26 ms. The time-dependent fluorescence signal was measured using a photomultiplier tube (PMT). At each concentration at least three individual traces were averaged and the rate constants and amplitudes were obtained by fitting of the data to an equation describing a double exponential function. The association and dissociation rate constants were determined by plotting *k*_obs_ against mantATP concentration and fitting of the data to a hyperbolic function, ${k}_{{obs}}=\frac{{k}_{\max }}{1+\frac{{k}_{1/2}}{[{mantATP}]}}+{k}_{d}$ to obtain the gradient (rate constant for mantATP association in M^−1^s^−1^) and the *y*-axis intercept (dissociation rate constant). The association constant was obtained by ${k}_{A}=\frac{{k}_{\max }}{{k}_{1/2}}$ and the dissociation constant by ${K}_{D}=\frac{{k}_{{off}}}{{k}_{\max }}.$ The analysis was done with the OriginPro 2022b.

### Circular dichroism

Circular dichroism (CD) spectra were recorded using a CD spectropolarimeter (MOS-500, BioLogic). The background signal from the buffer solution (20 mM Tris pH 8.5, 10 mM NaCl, 5 mM MgCl_2_) was subtracted from all the measurements. The optical pathlength of the cell was 1 mm and the concentration of OaPAC was 3 μM. The final concentrations of ATP were 30 μM and 100 μM. All measurements were performed in duplicates at 20 °C and each spectrum represents the average of 10 scans over the range 190–260 nm. To estimate the secondary structure content, the millidegrees ellipticity was converted to mean residue molar ellipticity using Eq. (4) where n is the number of peptide bonds and ellipticity is the raw data from the instrument

4 $$\theta \left(\deg * {{cm}}^{2}* {{dmol}}^{-1}\right)=\frac{{ellipticity}\left({mdeg}\right)* {10}^{6}}{{pathlength}\,\left({mm}\right)* \left[{protein}\right]\left(\mu {\rm M}\right)* n}$$

The BeStSel web server (http://bestsel.elte.hu/) was used to analyze the far-UV CD spectra in terms of the secondary structure content^34^. The reconstructed spectra were essentially superimposable on the experimental data over the wavelength range 195–250 nm with a low rmsd (normalized root mean square deviation) value.

### Reporting summary

Further information on research design is available in the Nature Portfolio Reporting Summary linked to this article.

## Results

### OaPAC retains a dimeric configuration at low concentrations

**Fig. 1:**
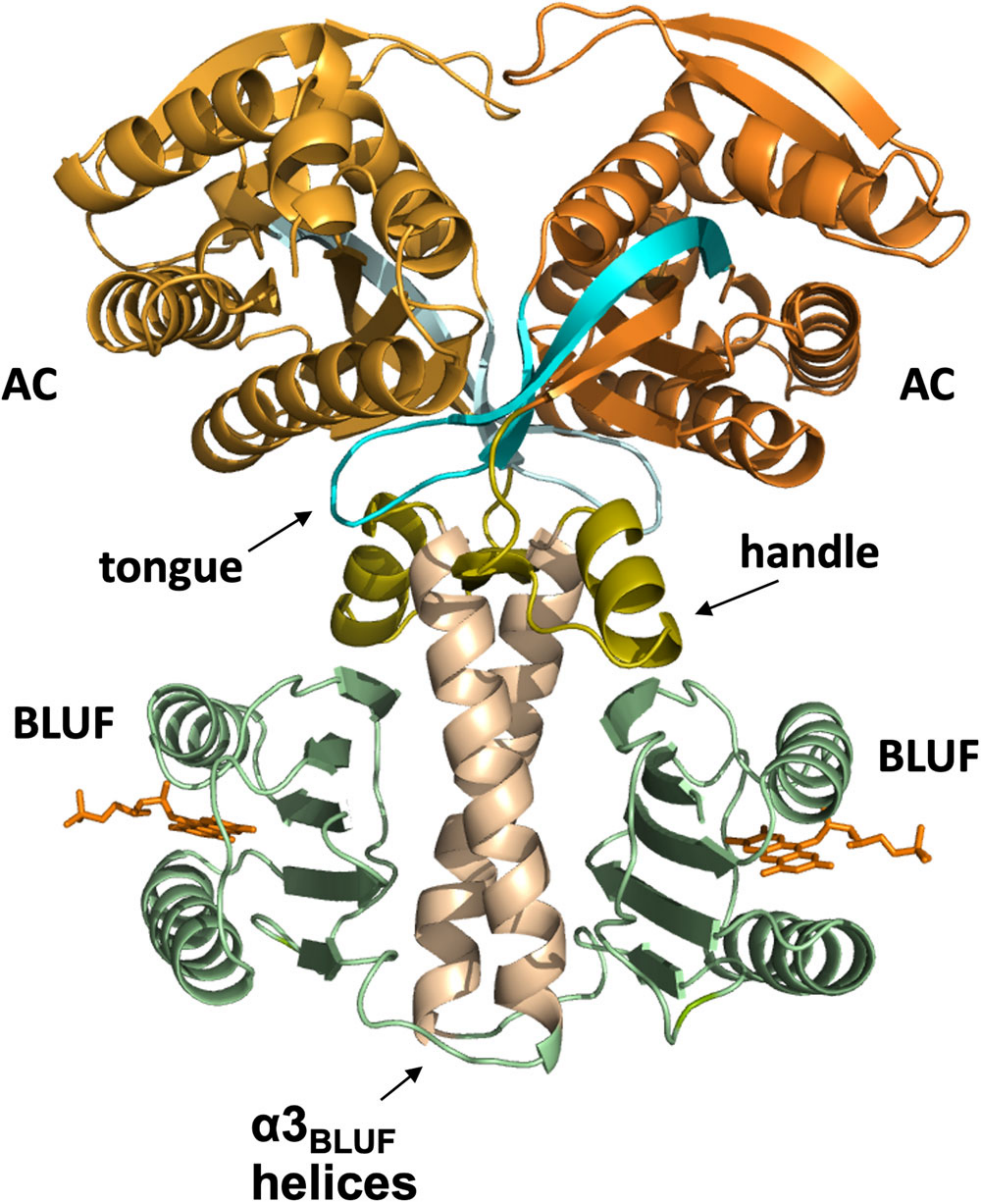
Crystal structure of OaPAC. Ribbon diagram of OaPAC (pdb:4yut) showing the BLUF and AC domains as well as the α3_BLUF_ helices, the handles and the β4_AC_-β5_AC_ central tongue discussed in the text.

Supplementary Fig. 1 shows the mass distribution for OaPAC (Fig. 1) and buffer obtained from a mass photometry (MP) measurement (Supplementary Note 1). Single molecules of OaPAC arrive at the cover slide of the MP set up and the binding events (Supplementary Movies 1 and 2) are converted to molecular mass with about 2–5% mass accuracy by performing a calibration with a set of proteins of known mass. Molecular masses of the events detected through out the movies of the buffer and OAPAC are also shown in Supplementary Fig. 1. The resulting mass distribution shows that the majority of the OaPAC molecules form a dimer with a complex mass of 85 kDa, in agreement with the theoretical calculated molecular weight (86 kDa) (Supplementary Fig. 1iv).

### Conformational dynamics of OaPAC is distinctly altered in presence of specific nucleotides

**Fig. 2:**
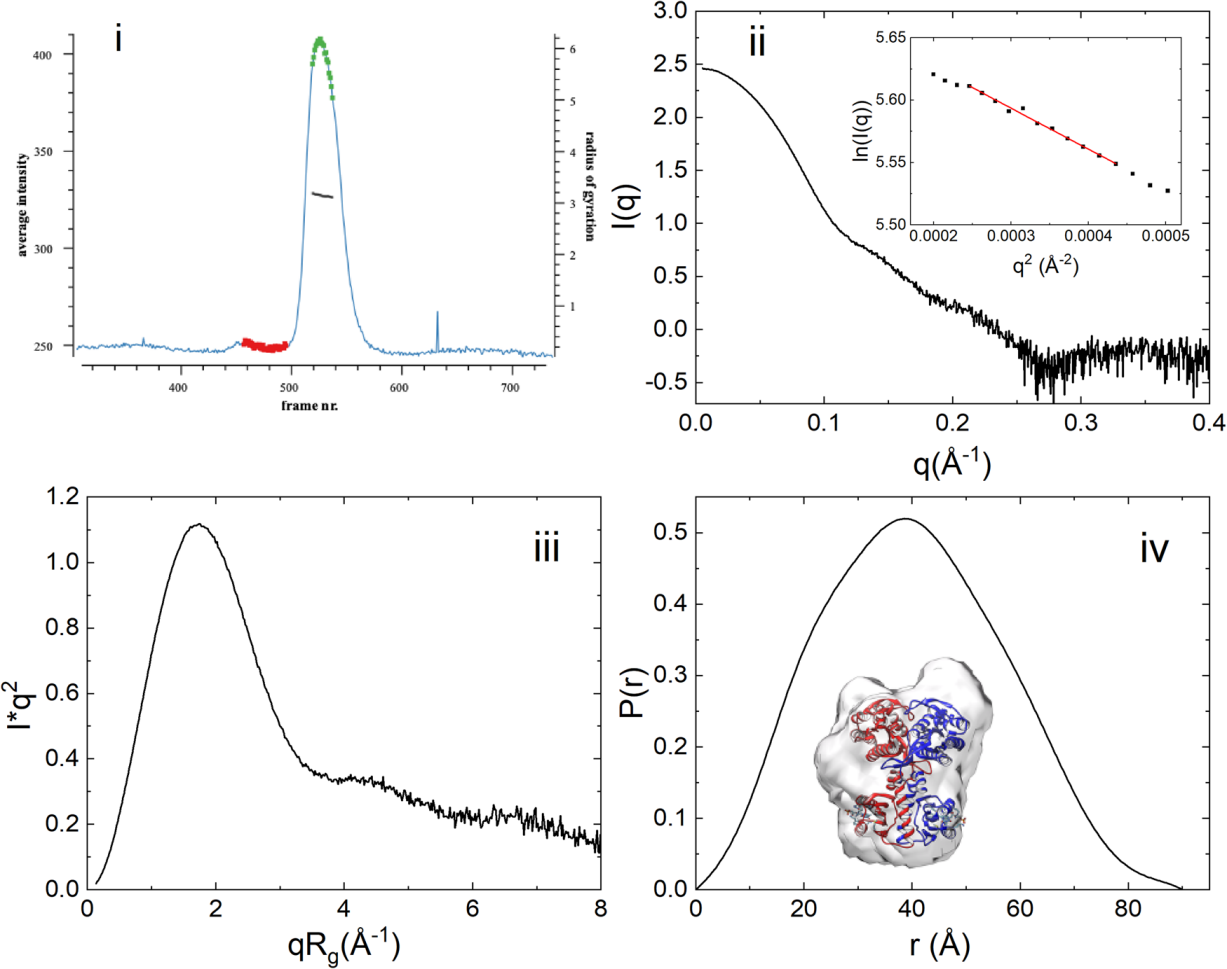
SAXS data from OaPAC subjected to size-exclusion (SEC-SAXS). (i) UV-trace (λ = 280 nm) of the eluting OaPAC in the SEC-SAXS experiment built by CHROMIXS, showing integrated intensities (light blue) versus time (frame number) for a SEC-SAXS run on OaPAC. Superimposed is the Rg-estimate for individual SAXS data-frames. The frames corresponding to the buffer (red dots) and sample (green dots) are automatically identified, averaged and subtracted one from another. (ii) X-ray scattering pattern of eluted OaPAC. Inset: Guinier plot of the data (iii) Dimensionless Kratky plot of OaPAC. (iv) Pair distribution function P(r) of OaPAC. Ab initio electron density envelope model (gray) based on the SAXS data is superimposed on the crystal structure of OaPAC (pdb:4yut).
ii. Effect of nucleotides on the conformation of OaPAC

i. Conformation of OaPAC in the absence of nucleotides
  Size-exclusion chromatography coupled SAXS (SEC-SAXS) has been employed to study the conformation of OaPAC in solution. Figure 2i shows the scattered intensity plot of nucleotide-free OaPAC from the in-line size exclusion chromatography where each point corresponds to the averaged scattering intensity from each individual frame and plotted as intensity versus frame number. The first and last frames of the chosen data set were indistinguishable, suggesting a monodisperse sample. The protein eluted has an estimated molecular mass of 91.7 kDa, derived from I(q) using SAXSMoW^35^. The sequence-based molecular mass is calculated to 43 kDa (Supplementary Fig. 1iv), this then suggests that in solution OaPAC is consistent with a dimer. Guinier analysis yields a radius of gyration, *R*_g_, of 31.4 ± 0.52 Å (Fig. 2ii, inset), with a particle volume of ~110,000 Å^3^. Inspection of the scattering profile of OaPAC using the dimensionless Kratky representation (Fig. 2iii) reveals a clear Gaussian peak at qRg = 1.7 and (qRg)^2^I(q)/I(0) = 1.1, suggesting a compact and globular shape that is also consistent with the shape of the distance distribution function, (*D*_max _= 90 Å) that displays only a slight anisometry (Fig. 2iv). Hence, it can be concluded that OaPAC adopts a quasi-globular conformation in solution. Supplementary Table 3 summarizes all SAXS derived parameters mentioned above whereas Fig. 2 shows all related SAXS curves. An ab initio electron density molecular envelope of OaPAC using the program DENSS (Supplementary Table 2) is also shown in Fig. 2iv. The corresponding SAXS parameters derived from the theoretical scattering curves are presented in Supplementary Table 3. It should be noted that these theoretically derived SAXS parameters from the crystal structures (4yus, 4yut) (Supplementary Fig. 2 in Supplementary Note 2) are smaller than the experimental ones from solution (Fig. 2, Supplementary Table 3). Also, the SAXS profiles of OaPAC at increasing concentrations (1, 2, 5, and 10 mg/ml) indicate that there are no appreciable concentration-induced conformational changes (Supplementary Fig. 3 in Supplementary Note 3, Supplementary Table 3).

**Fig. 3:**
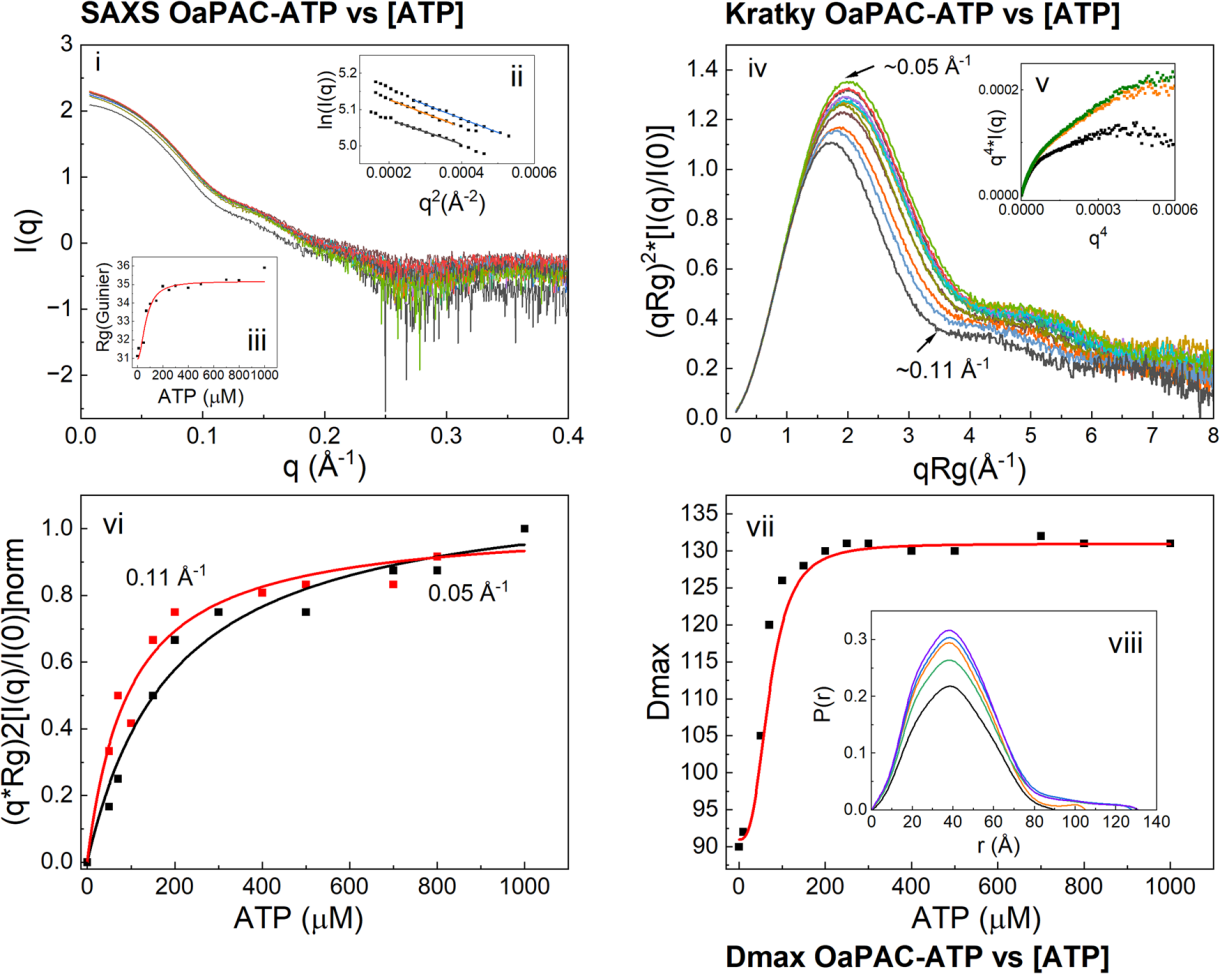
SAXS data from titration series of OaPAC with ATP. (i) SAXS profiles of OaPAC in the presence of various concentrations of ATP [ATP]. (ii) Guinier plots shown for 50 μΜ (black line), 100 μΜ (red line) and 150 μΜ (blue line) [ATP]. (iii) Plot of Rg derived from the Guinier analysis vs [ATP]. (iv) Dimensionless Kratky plots of OaPAC in the presence of various concentrations of ATP. (v) Porod-Debye plots of OaPAC (black line) and of OaPAC in the presence of 10 μΜ ATP (orange line) and 200μΜ ATP (green line). (vi) Plot of the Kratky values vs [ATP] at *q* = 0.05 Å^−1^ and *q* = 0.11 Å^−1^ (vii) Plot of Dmax derived from the pair distribution function (viii) vs [ATP]. (viii) Pair distribution function P(r) at selected ATP concentrations (0 μΜ: black line, 50 μΜ: orange line, 150 μΜ: blue line, 300 μΜ: green line, 1000 μΜ: violet line).

- Effect of adenosine triphosphate (ATP)
  Figure 3i shows the scattering profiles of OaPAC in the presence of increasing concentrations of ATP and representative Guinier plots (Fig. 3ii) indicating that OaPAC increases in size with the addition of ATP. After plotting the Rg as a function of ATP concentration (Table 1), fitting a hyperbolic equation yields an apparent equilibrium constant for OaPAC expansion (*K*_Rg_,_app_ = 70.13 ± 12.6 μM) (Fig. 3iii). This concentration is nearly identical to the concentration of ATP required to occupy one binding site in the AC domain ([OaPAC] = 70 μΜ or 3 mg/ml). As expected, the addition of increasing amounts of ATP to OaPAC also results in a shift of the normalized Kratky peak (Fig. 3iv), which is linked to the Rg (Fig. 3vi) and a corresponding increase in the Dmax of the enzyme (Fig. 3vii, viii) which is also described with an apparent *K*_Dmax_,_app_ = 71 ± 8.7 μM, similar to *K*_Rg_,_app_. Figure 3v shows the Porod-Debye plots of ATP-free OaPAC and of OaPAC in the presence of ATP (10 μΜ and 200 μΜ). The Kratky values (Fig. 3iv) at the maximum *q* = 0.05 Å^−1^ and at the minimum *q* = 0.11 Å^−1^ were plotted against the ATP concentration and provided the apparent equilibrium constants *K*_q=0.05_ = 194 ± 35 μΜ and *K*q_=0.11_ = 95 ± 15 μΜ. Τable 1 summarizes the basic structural parameters calculated from the data.

**Table 1:** Basic SAXS parameters R_g_ (radius of gyration), D_max._ (maximum size) and Rg(GNOM) (radius of gyration calculated using GNOM) derived from the SAXS profiles of the titration series of OaPAC with ATP (Fig. 3).

[ATP_final_]	Molar ratio (ATP:OaPAC)	R_g_(Å) (Guinier) /r^2^ (fit)	ΔR_g_(Å) (Guinier)	R_g_(Å) (GNOM*)	ΔR_g_(Å) (GNOM)	*D*_max_ (Å) /χ^2^
0 μΜ	–	30.63 ± 0.31 /0.9790	–	30.65 ± 0.02	–	90 /1.0281
10 μΜ	0.14	31.54 ± 0.48 /0.9873	0.91	30.44 ± 0.02	−0.21	92 /1.0556
50 μΜ	0.71	31.84 ± 0.67 /0.9857	1.21	31.04 ± 0.04	0.39	105 /1.2875
70 μΜ	1.0	33.57 ± 0.41 /0.9921	2.94	33.21 ± 0.05	2.56	120 /1.2590
100 μΜ	1.4	33.95± 0.62 /0.9902	3.32	32.81 ± 0.05	2.16	126 /1.0839
150 μΜ	2.1	34.12 ± 0.39 /0.9957	3.49	33.94 ± 0.05	3.29	128 /1.0093
200 μΜ	2.9	34.90 ± 0.38 /0.9937	4.27	34.31 ± 0.06	3.66	130 /1.0808
250 μΜ	3.6	34.70 ± 0.61 /0.9903	4.07	34.28 ± 0.05	3.63	131 /1.0616
300 μΜ	4.3	34.93 ± 0.38 /0.9906	4.3	34.02 ± 0.06	3.37	131 /1.1655
400 μΜ	5.7	34.82 ± 0.50 /0.9930	4.19	34.43 ± 0.05	3.78	130 /1.0904
500 μΜ	7.1	35.01 ± 0.66 /0.9888	4.38	34.16 ± 0.05	3.51	130 /1.1407
700 μΜ	10.0	35.25 ± 0.65 /0.9867	4.62	33.93 ± 0.06	3.28	132 /1.1421
800 μΜ	11.4	35.23 ± 0.45 /0.9913	4.6	33.67 ± 0.05	3.02	131 /1.1337
1000 μΜ	14.3	35.91 ± 0.41 /0.9817	5.28	33.35 ± 0.05	2.7	131 /1.4938

GNOM^22^ is an indirect transform program for small-angle scattering data processing. It reads in one-dimensional scattering curves (possibly smeared with instrumental distortions) and evaluates the particle distance distribution function P(r). χ^2^ is the chi square. All residual plots are presented in Supplementary Fig. 4 (Supplementary Note 4).
- Effect of adenosine-5’-[(α,β)-methyleno]triphosphate (ApCpp) and cyclic adenosine monophosphate (cAMP)

**Fig. 4:**
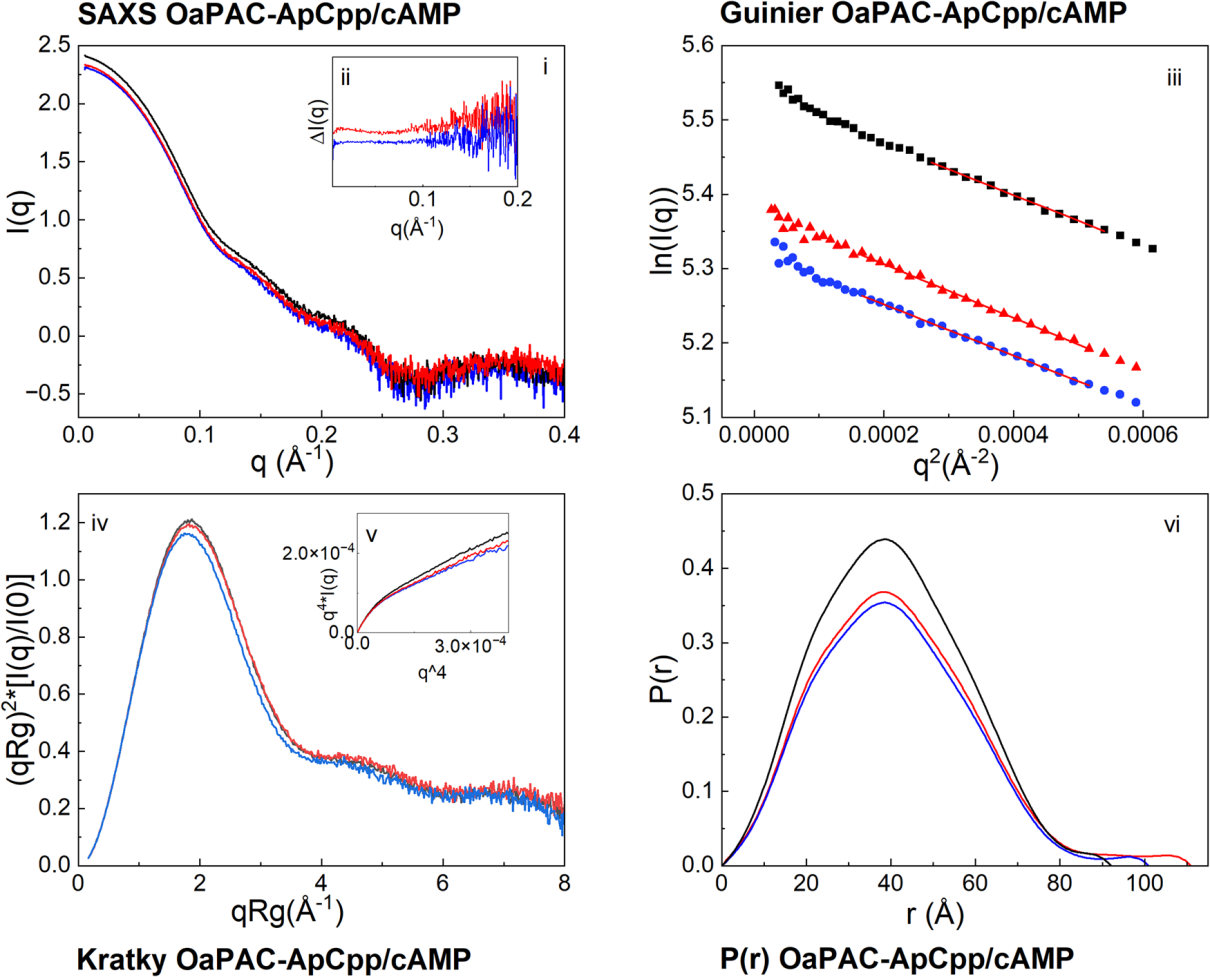
SAXS data of the OaPAC complexes with ApCpp and cAMP. (i) SAXS profiles of OaPAC and OaPAC in the presence of ApCpp and cAMP. (ii) Difference SAXS profiles of the OaPAC-ApCpp and OaPAC-cAMP complexes created after the subtraction of substrate-free OaPAC. (iii) Guinier plots, (iv) Kratky plots, (v) Porod-Debye plots, and (vi) Pair distribution function P(r) of OaPAC and of its complexes with ApCpp and cAMP. OaPAC (black line), OaPAC with ApCpp (red line), OaPAC with cAMP (blue line).

Figure 4 shows the (i) scattering profiles, (iii) the Guinier analysis, (iv) the Kratky plots, (v) the Porod-Debye plots, and (vi) the pair distribution functions of OaPAC and of OaPAC after the addition of either ApCpp or cAMP. The difference SAXS profiles of the OaPAC-ApCpp and OaPAC-cAMP complexes created after the subtraction of substrate-free OaPAC are shown in Fig. 4ii. Addition of the non-hydrolyzable ATP analogue, ApCpp, does not induce a similar increase in R_g_ and *D*_max_ values (Table 2, Fig. 4iii, vi, red line) as observed for ATP whereas an increase of ~1 Å is observed for the R_g_ and ~20 Å for the *D*_max_ of the OaPAC-ApCpp complex compared to the nucleotide-free OaPAC. On the other hand, cAMP does not induce any significant changes in these values (Table 2, Fig. 4iii, vi, blue line).

**Table 2:** Basic SAXS parameters Rg (radius of gyration), Rg(GNOM) (radius of gyration calculated using GNOM) and *D*_max_ (maximum size) calculated from the SAXS profiles of OaPAC incubated with cAMP and ApCpp.

SAXS parameters	OaPAC	OaPAC-cAMP	OaPAC-ApCpp
R_g_(Å) Guinier/r^2^	32.5 ± 0.2 /0.9977	32.1 ± 0.3/0.9966	32.9 ± 0.3/0.9987
R_g_(Å) (GNOM)	30.8 ± 0.01	31.01 ± 0.02	31.7 ± 0.03
D_max_ (Å)/χ^2^	92/2.0454	101/1.3059	111/1.3076

### ATP shows a moderate binding affinity to OaPAC

**Fig. 5:**
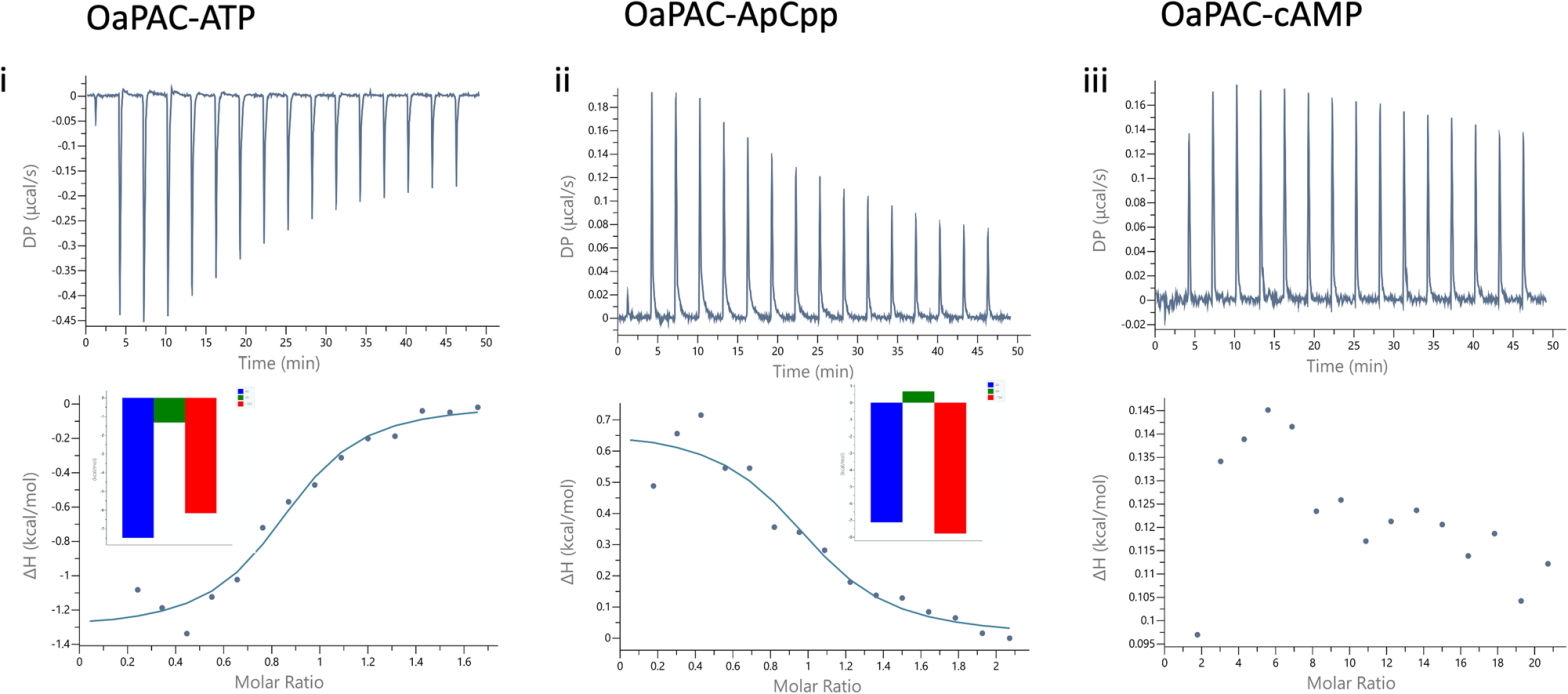
Calorimetric titrations for the binding of nucleotides to OaPAC. Isothermal titration calorimetry (ITC) profiles corresponding to the binding of (i) ATP, (ii) ApCpp and (iii) cAMP to OaPAC. The upper panels show the raw data for the titration of nucleotides with OaPAC and the lower panels show the integrated heats of binding obtained from the raw data, after subtracting the heat of dilution. The solid lines in the lower panels represent the best fits to the experimental data, using the *one set of sites* model from the MicroCal PEAQ Analysis Software. No binding was observed for cAMP (iii). The binding signature plots (thermodynamic signatures; ΔG: blue, Δ*H*: green, -Δ*TS*: red) are also displayed for the binding events for ATP and ApCpp (inset in the lower panels).

Isothermal titration calorimetry (ITC) assays were employed to determine the binding affinity and the thermodynamic parameters of the interaction of OaPAC with the nucleotides ATP, ApCpp and cAMP. Titration of ATP to OaPAC produced exothermic peaks (Fig. 5i). A fitting of the binding isotherm indicates that one ATP molecule binds per protein monomer with a dissociation constant, *K*_D_ = 3.24 ± 1.9 μM. The binding is both entropy and enthalpy driven, showing a positive favorable Δ*S* (*T*Δ*S* = 6.17 kcal/mol) and a negative favorable Δ*H* (Δ*H* = −1.32 ± 0.16 kcal/mol). In contrast, titration of OaPAC with ApCpp produced endothermic peaks (Fig. 5ii). One molecule of ApCpp binds per protein monomer with a dissociation constant *K*_D_ = 5.92 ± 5.06 μM. Fitting of the binding isotherm reveals that the binding is entropy driven as it is characterized by a positive entropy change (*T*Δ*S* = 7.76 kcal/mol) and an unfavorable positive enthalpy change (Δ*H* = 0.67 ± 0.13 kcal/mol). Figure 5i and ii also show the binding signature plots for both nucleotides. No binding was observed when OaPAC was titrated with cAMP as indicated by the presence of constant peak sizes (Fig. 5iii). Table 3 summarizes the thermodynamic parameters for the binding of all three nucleotides.

**Table 3:** Thermodynamic parameters of nucleotide binding in OaPAC.

Ligand	*N*	*K*_*D*_ (μM)	Δ*G* (kcal/mol)	Δ*H* (kcal/mol)	-*T*Δ*S*(kcal/mol)
ATP	0.83 ± 0.04	3.24 ± 1.9	−7.49	−1.32 ± 0.16	−6.17
ApCpp	0.97 ± 0.09	5.92 ± 5.06	−7.13	0.67 ± 0.13	−7.8

*N* number of binding sites, *K*_*D*_ dissociation constant, Δ*G* Gibbs energy, Δ*H* binding enthalpy, Δ*S* entropy.

### Nucleotide binding elevates the thermodynamic stability of OaPAC

**Fig. 6:**
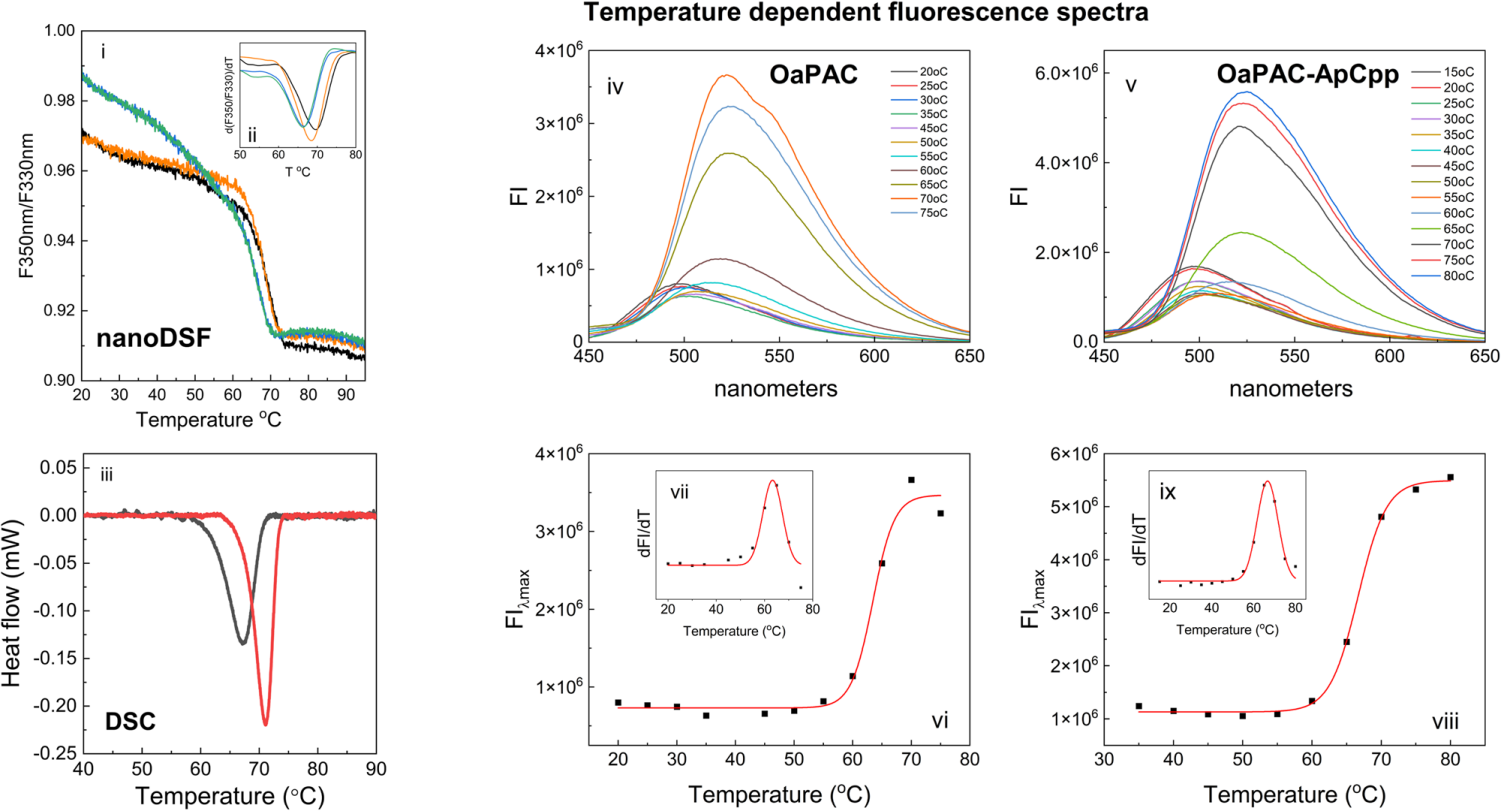
Determination of melting temperatures for OaPAC and its complexes with ATP and ApCpp using fluorescence based nanoDSF, the DSC technique and emission of the flavin during enzyme unfolding. (i) Tryptophan fluorescence ratio (F350nm/F330nm), (ii) the first derivative of the fluorescence ratio of OaPAC (green), OaPAC-ATP (orange), OaPAC-ApCpp (black) and cAMP (blue). (iii) DSC denaturation curves of OaPAC (black line) and its complex with ApCpp (red line). The heat flow measured in mW is plotted against temperature after baseline correction. (iv) temperature dependent fluorescence spectra of OaPAC. (v) temperature dependent fluorescence spectra of OaPAC and its complex with ApCpp. (vi) Plot of the emission maxima of OaPAC against the temperature (vii) First derivative of the fluorescence emission of OaPAC as a function of the temperature (viii) Plot of the emission maxima of the OaPAC-ApCpp complex against the temperature (ix) First derivative of the fluorescence emission of OaPAC as a function of the temperature.

We used two thermal shift assays based on fluorescence as well as differential scanning calorimetry to measure OaPAC denaturation and stability in the absence and presence of nucleotides. Protein unfolding was monitored using the distinct signatures of the fluorescence emission spectra of (i) tryptophan in a hydrophobic/hydrophilic environment and (ii) the free flavin.

i. The changes in fluorescence upon thermal unfolding of OaPAC and its complexes with ATP and ApCpp were monitored using nanoDSF. This approach uses the intrinsic tryptophan fluorescence of the protein which has an emission maximum around 330 nm in a hydrophobic environment and around 350 nm in a hydrophilic environment. In OaPAC and its nucleotide-bound complexes the ratio F350/F330 drops following a sigmoidal curve (Fig. 6i). An apparent melting temperature (*T*m) is obtained from the inflection point (Fig. 6i) or the first derivative of the curve (Fig. 6ii). The *T*m value for OaPAC is 66.31 °C whereas that of the ATP and ApCpp complexes is 68.46 °C and 69.5 °C, respectively (Table 4). No changes are observed for the melting point of OaPAC upon addition of the product cAMP.

**Table 4:** Thermal stability of OaPAC in the presence of ApCpp and ATP as measured by temperature dependent fluorescence (nanoDSF and emission of the flavin) and DSC.

Nucleotides	*T*m (^o^C) nanoDSF	*T*m (^o^C) emission flavin	*T*m (^o^C) DSC
no nucleotide	66.31 ± 0.06	63.4 ± 0.5	67.2
ApCpp	69.5 ± 0.14	66.6 ± 0.19	71.1
ATP	68.46 ± 0.01	n.m.	n.m
cAMP	66.49 ± 0.01	n.m.	n.m

*n.m*. not measured
ii. The thermodynamic stability of the ApCpp-bound OaPAC complex was also investigated using DSC. Figure 6iii shows the DSC thermographs of OaPAC and its ApCpp complex. OaPAC denatures in the temperature interval 58–72 °C with a transition midpoint at 67.2 °C (*T*m), whereas its ApCpp complex displays a higher *T*m of 71.10 °C with a denaturation temperature interval shifted to higher temperatures (between 63 °C and 74 °C). The calorimetric enthalpy ΔH^cal^ (enthalpy difference between folded and unfolded states) was calculated to be 0.184 J/g for OaPAC and 0.211 J/g for the OaPAC-ApCpp complex.
iii. The measured temperature dependent fluorescence spectra of OaPAC and its complex with ApCpp are shown in Fig. 6iv and Fig. 6v, respectively. In both cases the emission maxima red shifted from 500 to 525 nm as the temperature increases, indicative of the unfolding of the protein and the exposure of the flavin in the solution. It should be mentioned that a similar red-shift was observed during chemical-induced unfolding of OaPAC after the addition of increasing amounts of the denaturing agent GuHCl. Unfolding of the protein results in the release of the flavin which has a characteristic emission maximum at 525 nm (Supplementary Fig. 5i and Supplementary Fig. 5ii in Supplementary Note 5). A plot of the emission maxima against the temperature follows a sigmoidal curve that represents cooperative unfolding of OaPAC (Fig. 6vi) and its complex with ApCpp (Fig. 6viii). Non-linear fitting to a Boltzmann equation gives the apparent melting temperature (*T*m) that occurs at the mid-point of the unfolding transition. *T*m is also easily identified by plotting the first derivative of the fluorescence emission as a function of the temperature (Fig. 6vii, ix). In the presence of ApCpp a significant increase in the melting point is observed (OaPAC *T*m: 63.4 ± 0.5 °C, OaPAC-ApCpp: 66.6 ± 0.19 °C) suggesting that nucleotide binding results in a more thermostable complex. Table 4 summarizes the melting point values obtained from all three methods discussed above (i, ii, iii).

### Binding kinetics of an ATP analogue to OaPAC provides evidence for nucleotide-induced conformational changes

**Fig. 7:**
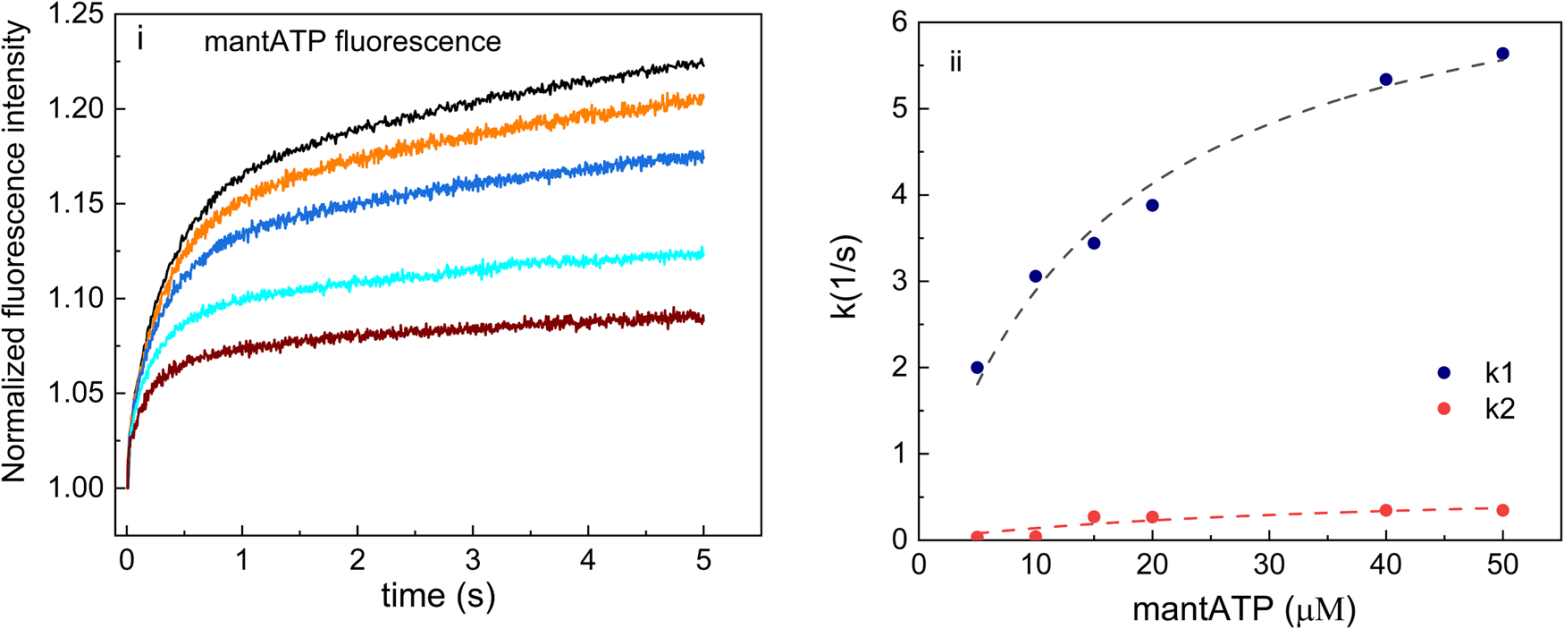
Steady-state kinetics of mANTP binding to OaPAC. (i) Time-dependent changes in fluorescence intensity of the mantATP upon binding to OaPAC measured by stopped-flow fluorescence spectroscopy (mantATP concentrations; black line: 10 μΜ, orange line: 15μΜ μΜ, blue line 20μΜ, cyan line: 40 μΜ, brown line: 50 μΜ) (ii) Plot of the rate constants (*k*obs) determined versus the mantATP concentration. Fitting of the data to a double exponential allows determination of both the association and the dissociation rate constant for mantATP from OaPAC.

mantATP is a hydrolysable, fluorescent ATP analogue that has been extensively used for kinetic studies^36–38^. OaPAC binds mantATP with a *K*_D_ = 2.25 ± 0.4 μM as determined by steady-state fluorescence anisotropy measurements (Supplementary Fig. 5iii in Supplementary Note 5). Binding of mantATP to OaPAC results in an increase of the quantum yield of mantATP by 50% compared to the quantum yield of mantATP in buffer. mantATP is well-known to exhibit an increased fluorescence intensity upon binding to proteins^39,40^ and hence it is an ideal fluorescent ATP analogue to characterize the kinetics of ATP binding by stopped-flow fluorescence spectroscopy. 4 μM OaPAC was rapidly mixed with mantATP at various concentrations (5–50 μM) using pseudo-first order conditions. The time (up to 5 s) dependent changes in the fluorescence intensity were monitored by exciting OaPAC at 350 nm and monitoring the emission of mantATP at 450 ± 20 nm (Fig. 7i). The averaged traces of a single concentration were fitted with a double exponential and the obtained rate constants (*k*_obs_) were plotted against the mantATP concentrations, in order to deconvolute the association and dissociation rate constants (Fig. 7ii). The biphasic kinetics of mantATP are characterized by a large, rapid increase (*k*_obs1_: 2–5.64 s^−1^) followed by a slower increase (*k*_obs2_: 0.07–0.347 s^−1^). For the fast phase, a hyperbolic fit of the observed rates vs mantATP concentration gives an asymptotic phase with a rate constant that corresponds to a maximum rate, *k*_max_ = 7.45 ± 0.62 s^−1^, a *K*_1/2_ = 30.01 ± 10.01 μM and a dissociation rate constant *k*_d_ = 1.001 ± 0.345 s^−1^ of mantATP from OaPAC. An association constant could be estimated *k*_*A*_ = 0.248 ± 0.08 μM^−1^s^−1^, whereas from the ratio of *k*_d_ and *k*_A_, a dissociation constant *K*_D_ could be extracted, *K*_D_ = 4.03 ± 1.90 μM. This value for *K*_D_ determined kinetically is in close agreement with the value determined by isothermal calorimetric titration for ATP (*K*_D_ = 3.24 ± 1.9 μΜ) (Fig. 5i, Table 3). The rates of the slow phase are slightly dependent of the concentration of mantATP. This phase can be attributed to the conformational changes in OaPAC induced by binding of mantATP as well as to the larger hydrodynamic radius of the mantATP-OaPAC complex. Table 5 summarizes the kinetic parameters derived from the binding of mantATP to OaPAC.

**Table 5:** Kinetic parameters for the binding of mantATP to OaPAC.

[mantATP] (μM)	*k*_obs1_ (s^−1^)	*k*_obs2_ (s^−1^)
5	2	0.07
10	3.058	0.17
15	3.44	0.271
20	3.88	0.268
40	5.34	0.346
50	5.64	0.347

### ATP binding induces small secondary structure changes in OaPAC

To evaluate if addition of ATP results in changes in the secondary structure of OaPAC, we recorded the CD spectra of OaPAC and its complex with ATP in two different concentrations (10-fold and ~30-fold excess). The CD spectrum of OaPAC in the far-UV region has a maximum at ~200 nm and signals in the 212–222 nm region characteristic of α-helical and β-structured elements^41^ (Supplementary Fig. 5iv in Supplementary Note 5). Addition of ATP results in a decrease of the signal at 212 nm with a concomitant increase of the signal at 222 nm, changes that are more notable as the ATP concentration increases. To deconvolute the contribution of each secondary element, the BeStSel (Beta Structure Selection) method was used^34^. The results of the analysis are summarized in Table 6 and demonstrate small secondary structure changes (~4–8% increase of the β-sheet content and ~7–9% decrease of the α-helical content) upon addition of ATP.

**Table 6:** Analysis of the structure of OaPAC from the CD data.

	OaPAC	+ ATP 30 μM	+ ATP 100 μM
helix	19.1	16.4	16.1
antiparallel β-sheet	15.2	15.6	15.8
parallel β-sheet	11.3	14.0	15.1
turn	14.3	14.6	13.7
others	40.0	39.4	39.3
nrmsd	0.01436	0.01474	0.01422

## Discussion

The photoactivated adenylate cyclase from *Oscillatoria acuminata* converts ATP to cAMP in a reaction that is stimulated 20-fold by light^9,11,12^. The signaling mechanism, however, remains unknown. A key step in understanding this mechanism is to obtain molecular level structural information on the interaction of the natural substrate ATP with OaPAC in its dark-adapted state in solution. Here, we present the first in-solution biophysical study that combines spectroscopic, biophysical and structural biology methods to shine light on this interaction and the binding dynamics of ATP to OaPAC. It should be pointed out that the basal activity of OaPAC in the dark is negligible as quantified in our previous work^42^ (Supplementary Fig. 6 in Supplementary Note 6) and also demonstrated in previous in vitro and in vivo studies^9^. Hence all following measurements reflect solely ATP binding to OaPAC and not a combination of ATP binding and catalysis.

### Molecular architecture of OaPAC in the absence of nucleotides

Using mass photometry, we accurately measured the mass of single molecules of OaPAC in solution confirming the dimeric nature of the enzyme at very low concentrations (100 nM) (Supplementary Fig. 1i). OaPAC is known to form a homodimer at μM concentrations as revealed by its crystal structure^9,10,14^. In addition, the molecular mass estimations from the SEC-SAXS data indicate a protein mass in the range 90–100 kDa (Supplementary Table 3) which is consistent with the sequence-based calculated value of 86 kDa (Supplementary Fig. 1iv) (molecular weight of the monomer 43 kDa). The dimeric nature of OaPAC has been also supported by the recent transient grating experiments by Nakasone et al. as well as by size-exclusion chromatography^43^ and analytical ultracentrifugation^9^. The Guinier region of the SEC-SAXS scattering curve gives a radius of gyration of 31.4 ± 0.52 Å, whereas analysis of the pair distribution function (P(r)) suggests a maximum intramolecular distance of 90 Å, providing an estimation of the overall size of the enzyme in solution. This latter value is significantly larger than the maximum distance observed in the crystal structure of OaPAC^9,10^ (~76 Å, pdb:4yut, Supplementary Fig. 7i in Supplementary Note 7), suggesting that the conformation of dimeric OaPAC in solution is less compact than that observed in the crystal structure. Our values for R_g_ and D_max_ are in line with the recently reported values of 30 ± 1 Å and 88 ± 3 Å, respectively for the dark-adapted state of OaPAC^43^. Our SAXS analysis indicates the sample was of sufficient quality for further ab initio analysis using DENSS. The ab initio molecular envelope constructed from the SECS-SAXS data of OaPAC (Fig. 2iv, Supplementary Fig. 7ii in Supplementary Note 7) shows a globular conformation for OaPAC which is in line with the globular conformation indicated by the dimensionless Kratky analysis (Fig. 2iii). The ab initio molecular envelope shows that the average conformation of OaPAC in solution is not fully represented by the crystal structure as indicated by comparison of the experimental SEC-SAXS curve and the calculated one (χ^2^ = 3.486) (Supplementary Fig. 7iii in Supplementary Note 7). In particular, the model is of the same size as the one based on the solution SEC-SAXS data, noted by the good match in the Guinier region; however there are some differences in the Porod region of the curve. This is probably caused by flexibility of loops or α-helices being more relaxed and in slightly different positions or orientations in the dimer cleft in solution when compared to the crystal structure. In line with the above, the calculated SAXS parameters derived from the crystal structures using the FoXS server are smaller than the experimental ones (Supplementary Table 3). This difference can be attributed to the fact that the protein volume in the crystal is reduced compared to that in solution due to the crystal packing and cryo-cooling^44^.

### Biophysical characterization of nucleotide binding to OaPAC

We have used a variety of biophysical methods to characterize the binding of ATP in the dark-adapted state of OaPAC. ATP binds to OaPAC with a dissociation constant *K*_D_ = 3.24 ± 1.9 μΜ as indicated by the ITC measurements. Similarly, the non-hydrolyzable ATP analogue, ApCpp and the fluorescent ATP analogue mantATP bind to OaPAC with a dissociation constant in the μM range (*K*_D_^ΑpCpp^ = 5.92 ± 5.06 μΜ, *K*_D_^mantATP^ = 2.25 ± 0.4 μM) in line with a moderate affinity of the substrate for the enzyme. It should be mentioned that the mant fluorophore has often little or no effect on the binding, dissociation or hydrolysis of the nucleotide when conjugated to the 2’ or 3’ position on the ribose in kinesins^38^ and hence it is not expected to affect significantly the binding mode of ATP in OaPAC.

Binding of the nucleotides results in a change in the protein environment due to new molecular interactions and/or conformational changes that can stabilize OaPAC through a reduction of the Gibbs energy of the complex. The increase in the Gibbs energy leads to an increase in the thermal stability which is reflected in the melting temperature (*T*m). Using differential scanning fluorimetry^31^ and differential scanning calorimetry^45,46^, we determined the *T*m values of OaPAC and its nucleotide complexes. In particular, we exploited the intrinsic fluorescence of the tryptophans as well as of the flavin chromophore of OaPAC to gain insight into the thermal stability of the enzyme and its complexes. In a hydrophobic environment, Trp fluorescence has a maximum at around 330 nm whereas in a hydrophilic environment is around 350 nm. OaPAC has three surface exposed tryptophan residues (W90, W303, W307) and many burried tyrosine residues. During heating-induced unfolding of the protein, the ratio F350/F330 drops due to the decrease of the fluorescence at 350 nm, following a sigmoidal curve that represents cooperative unfolding of OaPAC (Fig. 6i). A similar sigmoidal curve was also obtained by plotting the emission maxima in the 500–530 nm range where the flavin emits versus the temperature (Fig. 6vi, 6viii). Flavin emission red-shifts from 500 to 525 nm as OaPAC and its OaPAC-ApCpp complex unfold and flavin gets exposed to the solvent. For both approaches, a non-linear fitting to a Boltzmann equation gives the apparent melting temperature (*T*m) that occurs at the mid-point of the unfolding transition. The thermal shift observed indicates the formation of a more thermostable complex upon nucleotide binding. A more thermostable complex is also suggested by the direct calorimetric DSC method, where we observe a positive thermal shift Δ*T*m = 3.9 °C upon ApCpp binding to OaPAC (Fig. 6iii). The differences in the *T*m values between the three methods (Table 4) are attributed to the different markers (aromatic residues versus flavin) used to monitor the unfolding of the protein and to the fact that *T*m is actually an apparent melting temperature as a 50% unfolding state does not necessarily correlate to a 50% change in the intrinsic fluorescence signal. Regardless of the method used, addition of the nucleotide results in a more thermostable protein as indicated by the slight increase of the *T*m value.

Further thermodynamic parameters on the binding of ATP and ApCpp to OaPAC were obtained by the ITC measurements. The ΔΗ and ΔG values for ATP binding were −1.32 ± 0.16 kcal/mol and −7.49 kcal/mol, respectively, characteristic for an exothermic reaction that is both enthalpy and entropy driven (Δ*H* < 0, Δ*S* > 0 favorable reaction). On the other hand, ApCpp binding is endothermic and entropic driven (Δ*H* > 0 and Δ*S* > 0). The interaction is spontaneous for both nucleotides as indicated by the negative Δ*G* values whereas the electrostatic force accompanied by hydrophobic binding forces may play a role in the binding. For ATP the interaction is also driven by hydrogen bonding and van der Waals forces. A stoichiometry of one molecule of nucleotide per OaPAC monomer was estimated for both ATP and ApCpp. Differences in the binding mode of ATP and the non-hydrolysable analogue ApCpp have also been revealed by the SAXS data (Figs. 3 and 4). Whereas ATP binding to OaPAC results in a ~4 Å and ~20–40 Å increase in the Rg and Dmax, respectively, the corresponding values for ApCpp are much smaller, 0.5–1 Å for the Rg and 13 Å for the Dmax.

The recent crystal structure of the dark-state ATP-OaPAC complex has allowed a better understanding of the exact configuration of the catalytic active site in the presence of the natural substrate^14^. ATP has been shown to be coordinated mainly via the phosphates with the adenosine moiety showing a flexibility^14^, in line with the available structural and mutagenesis studies on the well characterized class III adenylate cyclases^47^. These studies have shown that the catalytic center is formed at the dimer interface whereas the active site configuration involves acidic residues for the coordination of divalent ions and basic residues for stabilization of the negatively charged ATP in the catalytic pocket^47^. Another structure that has provided insight at the atomic level on the interaction of PACs with ATP is that of the ATP complex of the Y7F mutant of bPAC (pdb:5mbk), which is a homologue of OaPAC^15^. Comparison of the SAXS theoretical profile of the ATP-bound bPAC-Y7F complex (in a pseudo-lit state) and the SAXS theoretical profile of the ATP-bound OaPAC complexes at room-temperature (pdb:8qfh) and 100 K (pdb:8qff)^14^ with the experimental SAXS profile of the ATP-bound OaPAC complex (in the dark state) (Supplementary Figure 8 in Supplementary Note 8) reveals a similar pattern. However, it should be mentioned that none of the available dark-state structures of type III AC domains in complex with ATP is considered to provide a fully active, nucleotide-bound conformation^14,15^, presumably due to the crystal packing and/or the conformation of OaPAC in the dark-adapted state.

### Binding mechanism of mantATP to OaPAC

Mant-labeled ATP has been shown to be an environmentally sensitive probe displaying an increase of the emission maximum upon exposure to the hydrophobic environment of the binding pocket of a protein and hence an excellent reporter molecule by which the kinetics of ATP binding and dissociation can be monitored^36–39^. In particular, a fluorescence quantum yield of ~0.7 was measured for mantATP after binding to OaPAC, using the classical method of Adams et al.^48^. The increase in the fluorescence of the molecule is attributed to the restricted motion of its adenine and anthraniloyl rings. We have observed a similar effect in the presence of high glycerol concentration (99%). mantATP binds to OaPAC with a dissociation constant in the μM range (*K*_D_^mantATP^ = 2.25 ± 0.4 μM) in line with the moderate affinity of the natural substrate, ATP for OaPAC (*K*_D_^ATP^ = 3.24 ± 1.9 μM) and the non-hydrolyzable ATP analogue, ApCpp (*K*_D_^ΑpCpp^ = 5.92 ± 5.06 μΜ). This is not unexpected as the mant fluorophore has been reported often to have little or no effect on the binding, dissociation or hydrolysis of the nucleotide when conjugated to the 2´ or 3´ position on the ribose^38^. The time-dependent changes in fluorescence intensity of the mantATP measured by stopped-flow fluorescence spectroscopy suggest a two-step binding mechanism. The fast phase which is dependent on the concentration of mantATP and therefore corresponds to the bimolecular association event and the slow phase which is slightly dependent of the concentration of mantATP and can be attributed to conformational changes occurring in OaPAC upon mantATP binding. However, it cannot be excluded that the slower kinetics might also arise to an increase of the hydrodynamic radius of OaPAC due to the binding of mantATP, similar to the increase of the radius of gyration upon ATP binding as observed by SAXS measurements. Hence the slower kinetics which show a small dependence on the concentration of ATP may arise from a combination of conformational changes and the larger hydrodynamic radius of the complex. This mechanism supports the structural plasticity within the ATP binding sides that may be essential for catalysis.

### Binding of ATP results in an expansion of OaPAC and an increase in its conformational flexibility

SAXS is one of the most powerful methods to assess the conformational transitions upon ligand binding in a protein in solution as well as changes in its flexibility and size. The structural equilibria of the various states of a protein solution can be described by using small-angle scattering parameters (e.g. R_g_, Dmax), which represent the average contribution to scattering of all of the particles in the protein^49^. It is well known that when a ligand binds to a protein, conformational changes take place which may involve large domain reorientations, accompanied by loop motions and sidechain movements and change in the radius of gyration of the protein^50^. ATP binding to OaPAC results in a significant increase of the Rg and D_max_ parameters (>3 Å and ~40 Å, respectively) suggesting an expansion of the enzyme. It also results in a more flexible enzyme as suggested by the changes in the Kratky plot. In particular, globular proteins like OaPAC display a maximum at 1.1 at qRg~1.73. ATP binding results in a widening and a shift of the Kratky peak from ~1.7 to 2 concomitant with an increase of the maximum from 1.1 to 1.35, indicative of an expansion of the enzyme and a more flexible structure. The lack of a plateau in the Porod-Debye plots^51^ (Fig. 3v) is also in line with a flexible structure for OaPAC which becomes even more flexible with ATP binding^51,52^. Moreover, the changes observed in the Kratky plot, in a maximum at *q* ~ 0.05 Å^−1^ (*λ* = q/π, ~57 Å) and in a minimum at *q* ~ 0.11 Å^−1^ (*λ* = q/π, ~125 Å) upon ATP binding (Fig. 3iv) reflect large-scale domain movements and smaller-scale structural modifications upon ATP binding. Fitting of the Kratky plot values plotted versus ATP concentration shows that both sets of data can be fit to a hyperbolic function with apparent equilibrium constants *K*_q = 0.05_ = 194 ± 35 μΜ and *K*_q = 0.11_ = 95 ± 15 μΜ. The pseudoequilibrium constant obtained for *q* = 0.11 Å^−1^ is similar to the apparent equilibrium constant obtained for Rg (*K*_Rg_, _app_ = 70.13 ± 12.6 μM). The agreement between these constants (*K*_q=0.11_ and *K*_Rg_, _app_) and the ATP concentration required suggests that one ATP molecule triggers the smaller-scale movements (q ~ 0.11 Å^−1^, Fig. 3iv) that result in an expansion of OaPAC, whereas larger-scale structural rearrangements (*q* ~ 0.05 Å^−1^, Fig. 3iv) which also take place could be tentatively attributed to the binding of the second ATP molecule, resulting in a further expansion of the enzyme. Such a mode of binding is in line with an allosteric mechanism for ATP binding. It should be noted that although the flexibility of the enzyme increases with increasing amounts of ATP, the compactness of OaPAC does not change significantly as the pair distribution function retains its symmetrical shape (Fig. 3viii). In line with that observation is the higher thermostability of the ATP-OaPAC complex, as indicated by the increased *T*m compared to the substrate-free OaPAC (Table 4). Although thermostability is generally associated with enhanced rigidity, it has been argued that thermal tolerance of a protein is not necessarily correlated with the suppression of internal fluctuations and mobility^53^. In support to that, enzymatic activity requires flexibility whereas the positive change in the entropy observed with substrate binding (Table 3) indicates an increase in disorder. It should be mentioned that conformational changes in OaPAC upon ATP binding have been also suggested from the stopped-flow data using mantATP (Fig. 7). Conformational changes and orientational flexibility upon nucleotide analog binding are a well-known feature in class III adenylate cyclases believed to reflect an induced-fit mechanism of substrate binding in which substrate binds to one active site and both change shape to create an ideal fit for catalysis^54^. Of particular note is the class IIIc AC isoform Rv1900c from *M. tuberculosis* which upon binding of an ATP analogue displays dramatic changes in the orientation of the two monomers (rotation by 16.6^o^ and translation by 11.4 Å) in the crystal form^55^. It is worth mentioning that in the recent dark-state structure of the ATP-bound OaPAC complex, the adenine moiety has been reported to be able to move freely within a 5 Å range of motion^14^, which suggests a flexibility of the AC domains. On the contrary, binding of the non-hydrolyzable ATP analogue, ApCpp, does not seem to induce major conformational changes in the structure of the enzyme^9^, in line with our present solution studies. Structural flexibility and a clamshell-like movement but in a smaller scale (1.8^o^ increase of the AC opening angle and 0.7 Å translation of the AC domain centers) has been reported to take place upon blue-light illumination of crystalline bPAC^15^. BLUF domains, on the other hand, are well-known to be rigid; however, the possibility that ATP binding is able to induce a flexibility in the BLUF domain cannot be excluded. It should be mentioned that conformational changes in the BLUF domain of OaPAC have been reported in crystals of the ATP-bound complex in the light-adapted state. In particular, a major structural rearrangement taking place in the loop region between the *β*4-strand and *β*5-stand and the loop between the *β*5-stand and the *α*3-helix accompanied by a flip of the residues Trp90 and Met92 in the vicinity of the flavin has been observed^14^. Studies in solution using transient absorption^56^ and transient grating methods^43^ have also indicated conformational changes in the C-terminal region in the absence of the natural substrate ATP under blue-light illumination.

### Proposed model for ATP binding to OaPAC

**Fig. 8:**
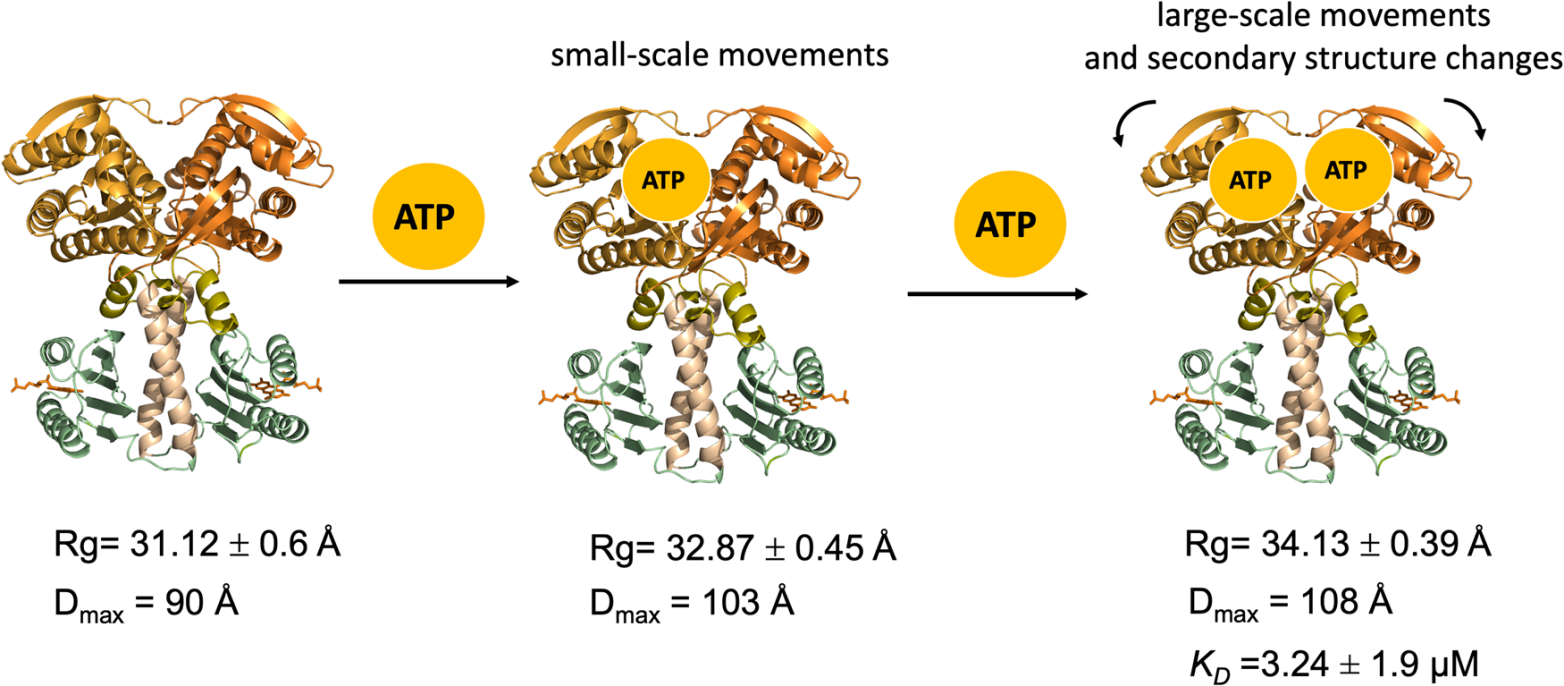
Binding of ATP results in significant conformational changes in the enzyme. Schematic diagram summarizing the findings of the present study.

Based on the integrative approach presented in this work, we can propose the following model for binding of ATP to OaPAC illustrated in Fig. 8. One ATP molecule binds per monomeric OaPAC in the dark-adapted state with a moderate affinity (*K*_D_ in the micromolar range), resulting in the formation of a thermodynamically more stable enzyme.

The interaction is spontaneous, exothermic and driven by hydrogen bonding, hydrophobic interactions and van der Waals forces. The flexibility of the enzyme is further enhanced without losing compactness with small changes in the secondary structure. Small scale movements take place during the binding of the first ATP molecule, whereas the binding of the second ATP molecule is characterized by larger scale movements. These conformational changes due to substrate binding are accompanied by a significant increase of the size of the enzyme. Rotational movements have been considered to be triggered by regulatory inputs present in the class III adenylate cyclases^55^. Such regulatory inputs have been identified in bPAC and are the α3_BLUF_ helices and the handles. Interaction of this regulatory domain with the β4_AC_-β5_AC_ central tongue (Fig. 1) is believed to adjust the AC opening and to prepare the active site for catalysis^15^. The same regulatory elements are present in OaPAC (Fig. 1) and whether a similar regulatory input from these elements to the catalytic mechanism of OaPAC can be proposed given the high homology between the two enzymes remains to be investigated.

Although it is well known that solution studies do not necessarily correlate with in vivo studies, the former allow the evaluation of many parameters at a molecular level, providing the opportunity to design the engineering of PACs with desired properties. This is the case for the current study, which allows us to obtain a better insight on the interaction of PACs with its natural substrate before the light-triggering event. Light-triggering of the AC activity at the cellular level has shown that OaPAC is able to control cell functions in zebrafish^57^, and can also work in different cell types, showing lower light sensitivity compared to other PAC proteins^58^ that allows for a finer control of the degree of stimulation^9^. In addition, bPAC has been targeted to different cell compartments (plasma membrane, outer mitochondrial membrane and nucleus), triggering the phosphorylation of a downstream effector, the protein kinase A (PKA) occupying the specific compartment^59^. Besides PKA^60^, other prominent effectors like the exchange protein Epac^61^, the hyperpolarization-activated cyclic nucleotide-gated ion channels (HCN)^62^ and the cyclic nucleotide-gated ion channels (CNG)^63^ have been the immediate recipients of light-stimulated PAC-derived cAMP. In particular, important information on the localization and trafficking of HCN channels and their response to dynamic cAMP signal transduction at the nanoscale level has been reported using various PACs^64^. Membrane-bound PACs have also enhanced the optogenetics toolbox by manipulating cAMP or K^+^-fluxes in *C.elegans*^65^. The above mentioned studies show clearly the vast potential of PACs in optogenetic applications. To that goal, enzymatic assays that can report on intracellular AC activity and screen efficiently engineered photoactivated adenylate cyclases^66^, as well as advanced imaging techniques like confocal microscopy and super-resolution microscopy can bring deeper insights on the molecular pathways in a native biological environment.

### Supplementary information

Supporting information

Description of Additional Supplementary Files

Supplementary Movie 1

Supplementary Movie 2

Reporting Summary

## Data Availability

All raw data and metadata from experiments and simulations are available from the corresponding authors on request.
