# Supplementary material for: ATP-dependent conformational dynamics in a photoactivated adenylate cyclase revealed by fluorescence spectroscopy and small-angle X-ray scattering
Source: Commun Biol. 2024 Feb 2;7:147. doi: 10.1038/s42003-024-05842-1 (PMC10837130; doi:10.1038/s42003-024-05842-1)
Supplement: Supplementary file 1 — Supporting information [file 42003_2024_5842_MOESM1_ESM.docx]

**Supporting Information**

**ATP-dependent conformational dynamics in a photoactivated adenylate cyclase revealed by fluorescence spectroscopy and small-angle X-ray scattering**

K. Ujfalusi-Pozsonyi^1^, E. Bódis^1^, M. Nyitrai^1^, A. Kengyel^1^, E. Telek^1^, I. Pécsi^1^, Z. Fekete^1^, N. Varnyuné Kis-Bicskei^1^, C. Mas^2^, D. Moussaoui^3^, P. Pernot^3^, M. D. Tully^3^, M. Weik^4^, G. Schirò^4^, S.M. Kapetanaki^4#*^, A. Lukács^1*^

^1^ Department of Biophysics, Medical School, University of Pécs, 7624 Pécs, Hungary

^2^ Univ. Grenoble Alpes, CNRS, CEA, EMBL, ISBG, F-38000 Grenoble, France

^3^ European Synchrotron Radiation Facility (ESRF), Grenoble, France

^4^ Institut de Biologie Structurale (IBS), Université Grenoble Alpes, CEA, CNRS, Grenoble, France

# present address: Department of Biophysics, Medical School, University of Pécs, 7624 Pécs, Hungary

*Correspondence: [sofia.kapetanaki@aok.pte.hu](mailto:sofia.kapetanaki@aok.pte.hu), [andras.lukacs@aok.pte.hu](mailto:andras.lukacs@aok.pte.hu)

Outline

- Supplementary Table 1. SAXS-data collection parameters.
- Supplementary Table 2. Software for SAXS data reduction, analysis and interpretation.

Supplementary Note 1: Mass photometry study on OaPAC.

Supplementary Note 2: Theoretical SAXS patterns derived from the crystal structures of OaPAC.

- Supplementary Note 3: Effect of the enzyme concentration on the oligomeric structure of OaPAC.
- Supplementary Note 4: Residual plots from Guinier and Pair Distribution Function analysis of selected SAXS data.
- Supplementary Note 5: Effect of GuHCl on OaPAC, calculation of the dissociation constant *K*_D_ of mantATP to OaPAC and circular dischroism spectra of OaPAC and its ATP complex.

Supplementary Note 6: The conversion of ATP to cAMP by OaPAC is negligible in the dark.

- Supplementary Note 7: Comparison of the experimental SAXS data and SAXS-derived models with the OaPAC crystal structure.
- Supplementary Note 8: SAXS curves of OaPAC and its complex with ATP and theoretical curves of bPAC-Y7F and OaPAC and their complexes with an ATP analogue and ATP, respectively.
- **Supplementary Table 1. SAXS-data collection parameters**

| **Beamline** | ESRF BM29 with Dectris PILATUS 1M detector |
| --- | --- |
| **Wavelength (nm)** | 0.992 |
| **Sample distance (m)** | 2.867 |
| **q measurement range (**Å^-1^**)** | 0.02 to 0.5 |
| **Absolute scaling method** | Data were scaled to absolute intensity against water to forward scattering at I(0), q=0. |
| **Normalization** | Direct beam |
| **Exposure time** | 10 times 1s frames |
| **Sample delivery** | Batch mode with robot sample changer (Arinax) |
| **Sample temperature (^o^C)** | 20 |

- **Supplementary Table 2. Software for SAXS data reduction, analysis and interpretation**

| **SAXS data reduction and basic analysis** | BioXTAS RAW |
| --- | --- |
| **Atomic structure modelling** | DENSS |

**Supplementary Note 1: Mass photometry study on OaPAC**

A short movie of the ratiometric frames (frame 450 to 900) of OaPAC at 50 nM was recorded showing a total of 1429 binding events and 133 unbinding events recorded in the full movie (5994 frames). Also, a short movie of ratiometric frames (frame 450 to 900) of the buffer alone was recorded and showing little noise signature with 135 binding and 145 unbinding events respectively in the full movie (5994 frames). After mass calibration, it is obvious that the main peak of the binding events at 85 kDa fitted with a Gaussian distribution corresponds to the dimeric form of OaPAC. As shown in the histogram, from the above comparison it is clear that the particles arriving on the coverslip are single molecules of dimeric OaPAC.

i


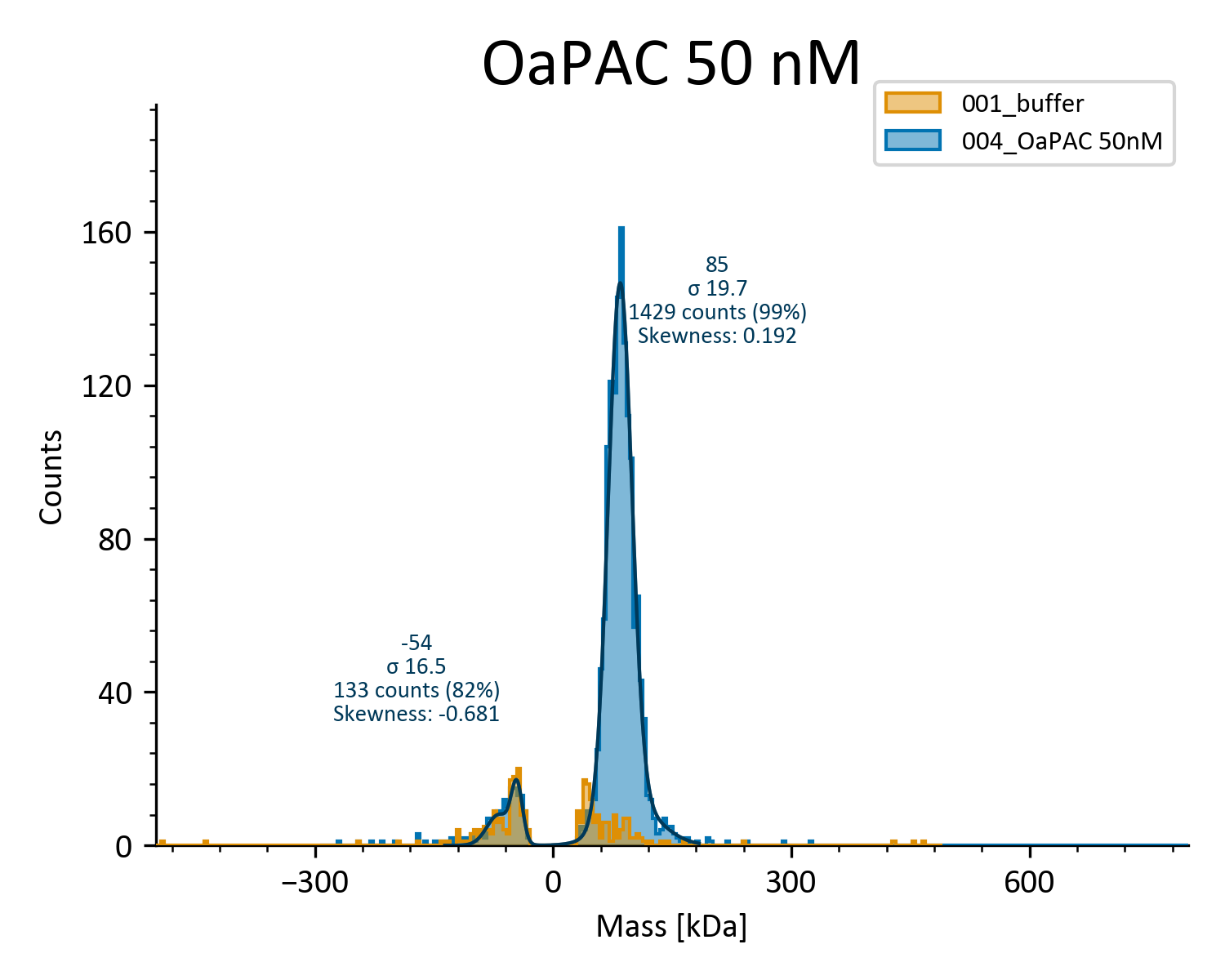


buffer

OaPAC

ii


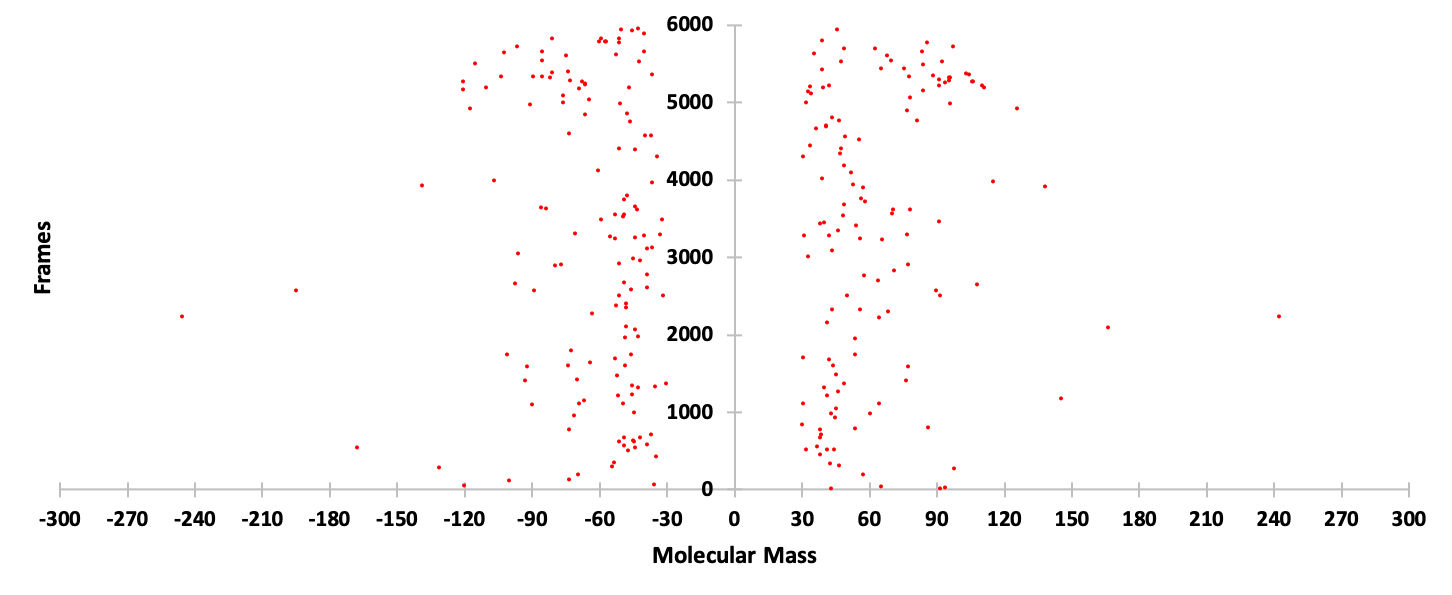


iii


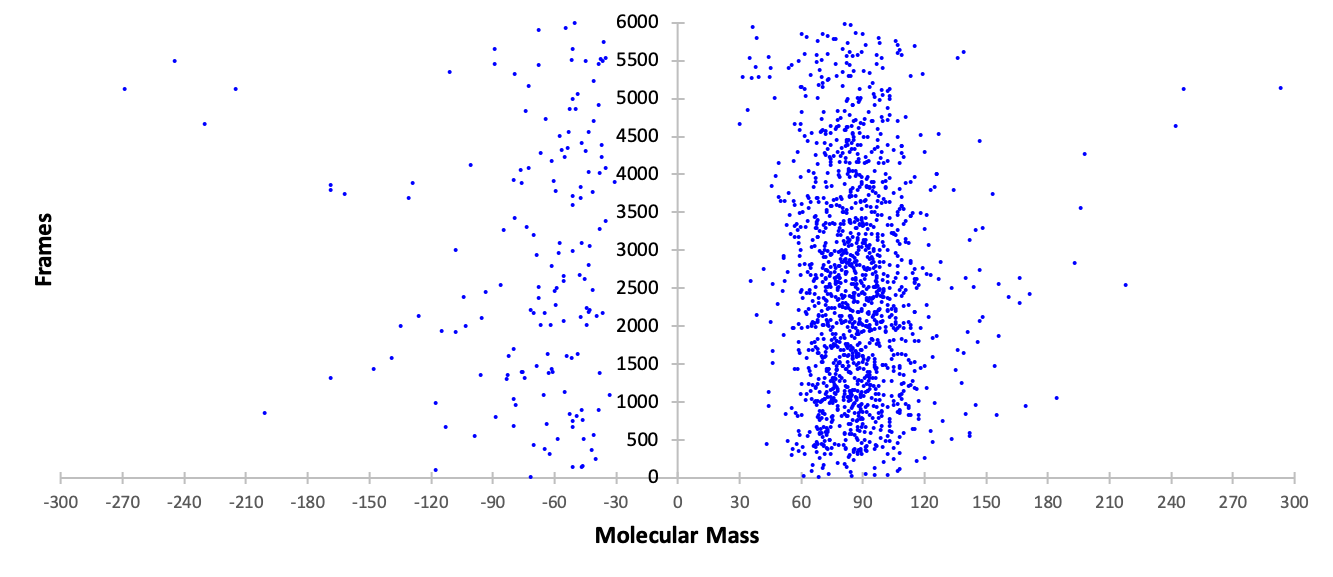


iv

MNHKVHHHHHHIEGRHMKRLTYISKFSRPLSGDEIEAIGRISSQKNQQANVTGVLLCLDGIFFQILEGEAEKIDRIYERILADERHTDILCLKSEVEVQERMFPDWSMQTINLDENTDFLIRPIKVLLQTLTESHRILEKYTQPSIFKIISQGTNPLNIRPKAVEKIVFFSDIVSFSTFAEKLPVEEVVSVVNSYFSVCTAIITRQGGEVTKFIGDCVMAYFDGDCADQAIQASLDILMELEILRNSAPEGSPLRVLYSGIGLAKGKVIEGNIGSELKRDYTILGDAVNVAARLEALTRQLSQALVFSSEVKNSATKSWNFIWLTDSELKGKSESIDIYSIDNEMTRKSSGGLEIARNIGHYLERVGDRQPSQIFGVKSLPL

MW: 43kDa

Supplementary Figure 1. i. Molecular mass distribution histogram of OaPAC (blue) and buffer (orange) obtained by mass photometry. The solid line represents a fit with a Gaussian function to the dimeric OaPAC peak. ii. Molecular masses of the events detected through out the movies for the buffer. iii. Molecular masses of the events detected though out the movies for OaPAC. Binding events for single molecules of OaPAC can be observed in the Supplementary movie 1. Binding events for single molecules of buffer can be observed in the Supplementary movie 2 iv. OaPAC sequence and molecular weight of the monomeric enzyme.

- **Supplementary Note 2: Theoretical SAXS patterns derived from the crystal structures of OaPAC**

A.


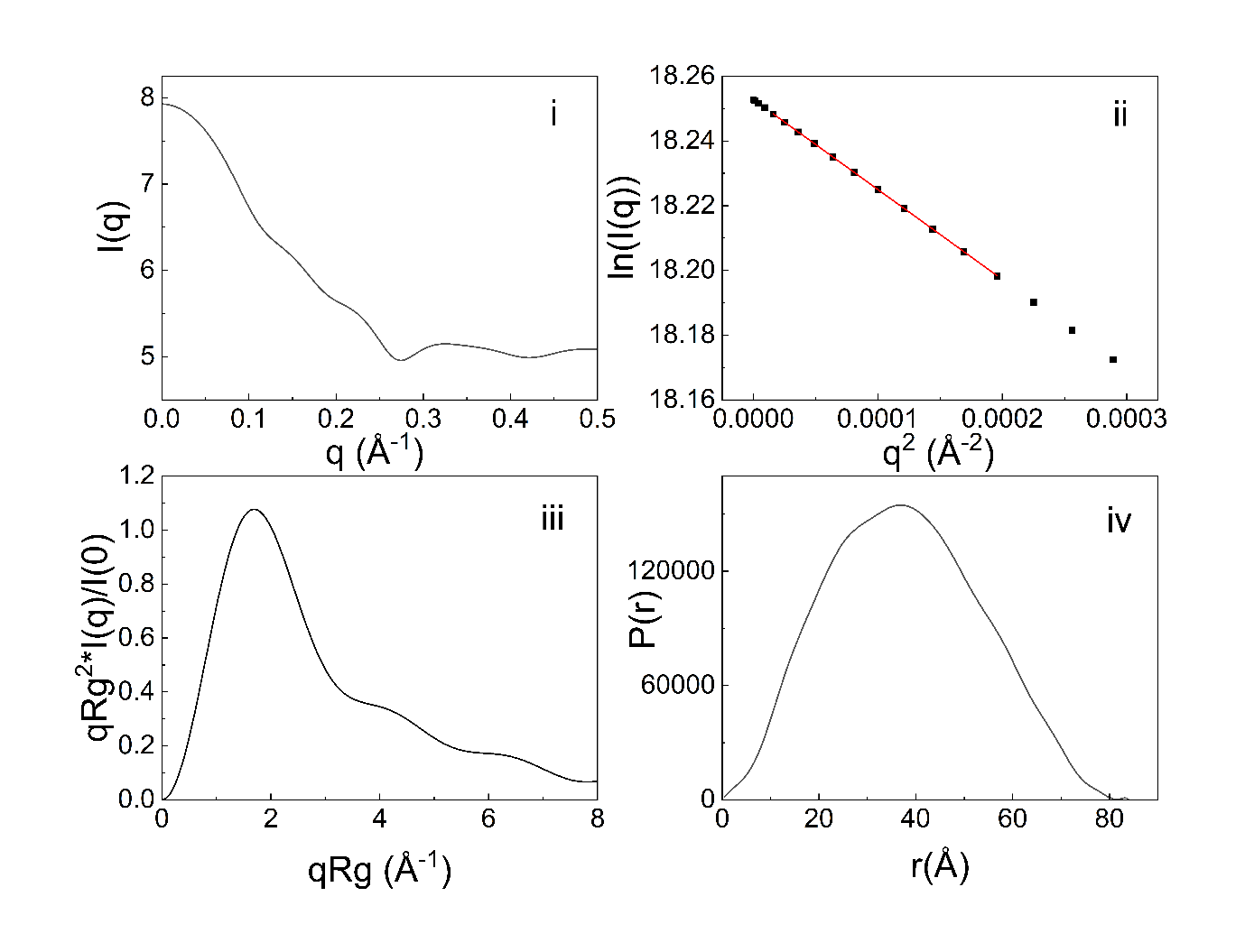


B.


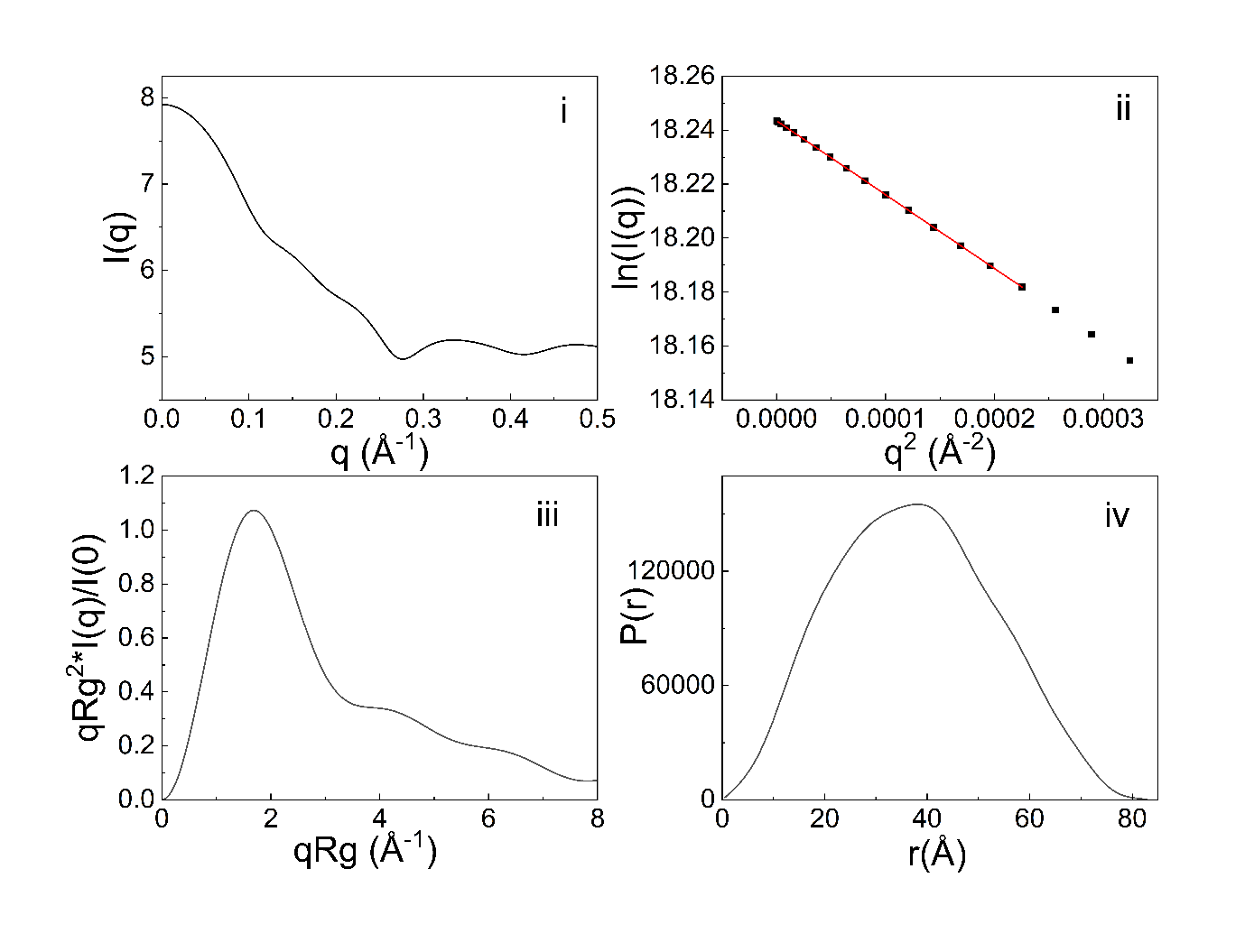


Supplementary Figure 2. Theoretical SAXS data derived from the available crystal structures of OaPAC using the FoXS server. A. pdb: 4yut B. pdb:4yus. i) theoretical SAXS profile ii) Guinier analysis iii) Kratky plot iv) Pair distribution function, P(r).

- **Supplementary Note 3: Effect of the enzyme concentration on the oligomeric structure of OaPAC**

In order to test for oligomerization or aggregation of the protein, we determined the scattering profiles of OaPAC at increasing concentrations (1, 2, 5, and 10 mg/ml). Figure S3 shows the X-ray scattering patterns at different concentrations which are characterized by the typical increase in I(0) with increasing concentration (Fig. S3vi). Guinier analysis of the SAXS curves of OaPAC at all concentrations (Fig. S3ii) reveals a deviation from the R_g_=31.4 ± 0.52 Å value estimated for the dimeric OaPAC probed immediately after the size-exclusion column. The *R*g values for all concentrations are summarized in Table S3. A bell-shaped Gaussian peak is observed for the Kratky plot (Fig. S3iv) at all four concentrations in line with the globular shape of the protein. However, at high concentration (10 mg/ml) the peak position deviates, suggesting strong interparticle effects and/or aggregation. The P(r) curves (Fig. S3v) are largely identical at all concentrations indicating that there are no appreciable concentration-induced conformational changes. The curves are single peaks with a tail at higher concentrations. This tail is absent in the eluted OaPAC (Fig. 2iv) pointing towards the presence of interparticle effects in the samples which have not been subjected to size-exclusion chromatography immediately before the SAXS measurement. The increase of the Rg value from the Guinier analysis and the shift of the Kratky peaks with increasing concentrations are also in line with that interpretation. We attribute these deviations to a minor population of aggregated structures as indicated by the presence of a small peak in the elution profile (frame ~450) (Fig. 2i).


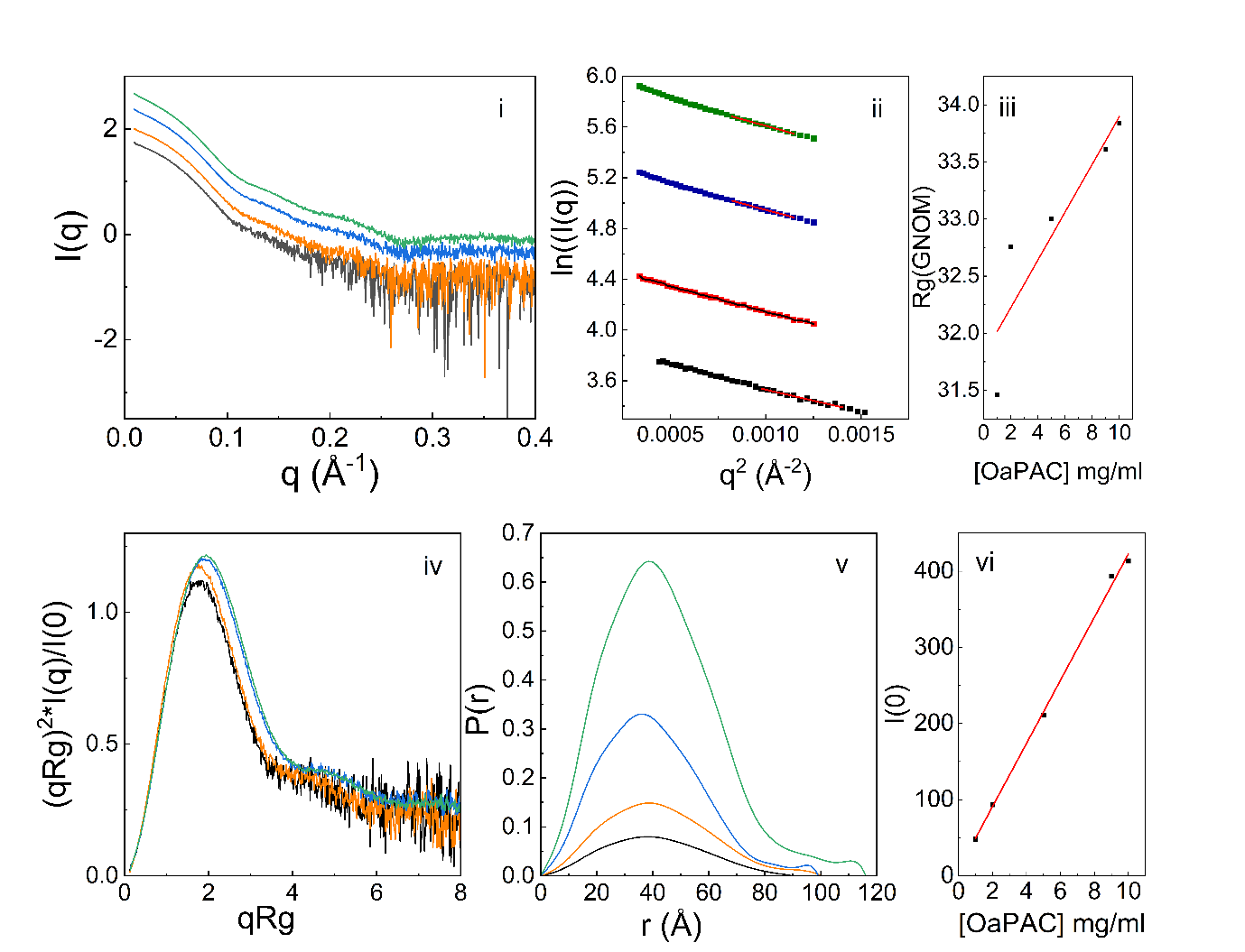


Supplementary Figure 3. i) X-ray scattering patterns, ii) Guinier plots iii) Plot of Rg versus OaPAC concentrations iv) Dimensionless Kratky plots, v) Normalized pair distribution functions P(r) of OaPAC at various concentrations vi) Plot of I(0) versus OaPAC concentrations. OaPAC concentrations: 1mg/ml (black line), 2mg/ml (orange line), 5mg/ml (blue line) and 10 mg/ml (green line).

**Supplementary Table 3**. Structural parameters derived from the SAXS data. Radius of gyration (R_g_) and maximum size (D_max_) calculated from the SAXS profiles for eluted OaPAC (SEC-SAXS) and OaPAC at different concentrations.

|  | **SEC-SAXS** | **1mg/ml** | **2mg/ml** | **5mg/ml** | **10mg/ml** | **4yus_dimer** | **4yut** |
| --- | --- | --- | --- | --- | --- | --- | --- |
| R_g_(Å)/r^2 | 31.4 ±  0.52  /0.9908 | 32.3 ± 0.67  /0.9697 | 34.05 ± 0.75  /0.9921 | 34.86 ± 0.3  /0.9979 | 35.3 ± 0.19  /0.9988 | 28.65 ± 0.53  /1 | 28.87 ± 0.5  /1 |
| Rg(Å) (GNOM) | 30.71 ±  0.01 | 31.46 ± 0.5 | 32.76 ± 0.05 | 33 ± 0.04 | 33.84 ± 0.04 | 28.63 ± 0.05 | 28.85 ± 0.04 |
| D_max_ (Å)  /χ^2^ | 90/1.0728 | 90  /1.1889 | 100  /1.4722 | 106  /1.5433 | 116  /1.8035 | 83  /2.95E-07 | 84  /2.40E-07 |
| Vp (Å^3^) | 110,000 | 110,000 | 121,000 | 121,000 | 124,000 | 93,000 | 95,700 |
| MW (kDa) | 91.7 | 91.5 | 100.3 | 100.1 | 102.7 | 77.2 | 79.4 |

4yus and 4yut refer to the pdb entries of OaPAC (4yus was solved at 1.8 Å with ApCpp added whereas 4yut was solved at 2.9 Å without the addition of any nucleotide). All residual plots are presented in Supplementary Figure 4.

- **Supplementary Note 4: Residual plots from Guinier and Pair Distribution Function analysis of selected SAXS data.**

Supplementary Figure 4. The following figures show: (left) the Guinier plot and linear fit (top) and the corresponding normalized residuals of the fit (bottom); (right) the P(r) function (top), the data (middle, blue points) and the fit line which is the Fourier transform of the P(r) function also called regularized intensity (middle, red line) and the fit residual (bottom) of the corresponding data in Table 1 and Table S3.


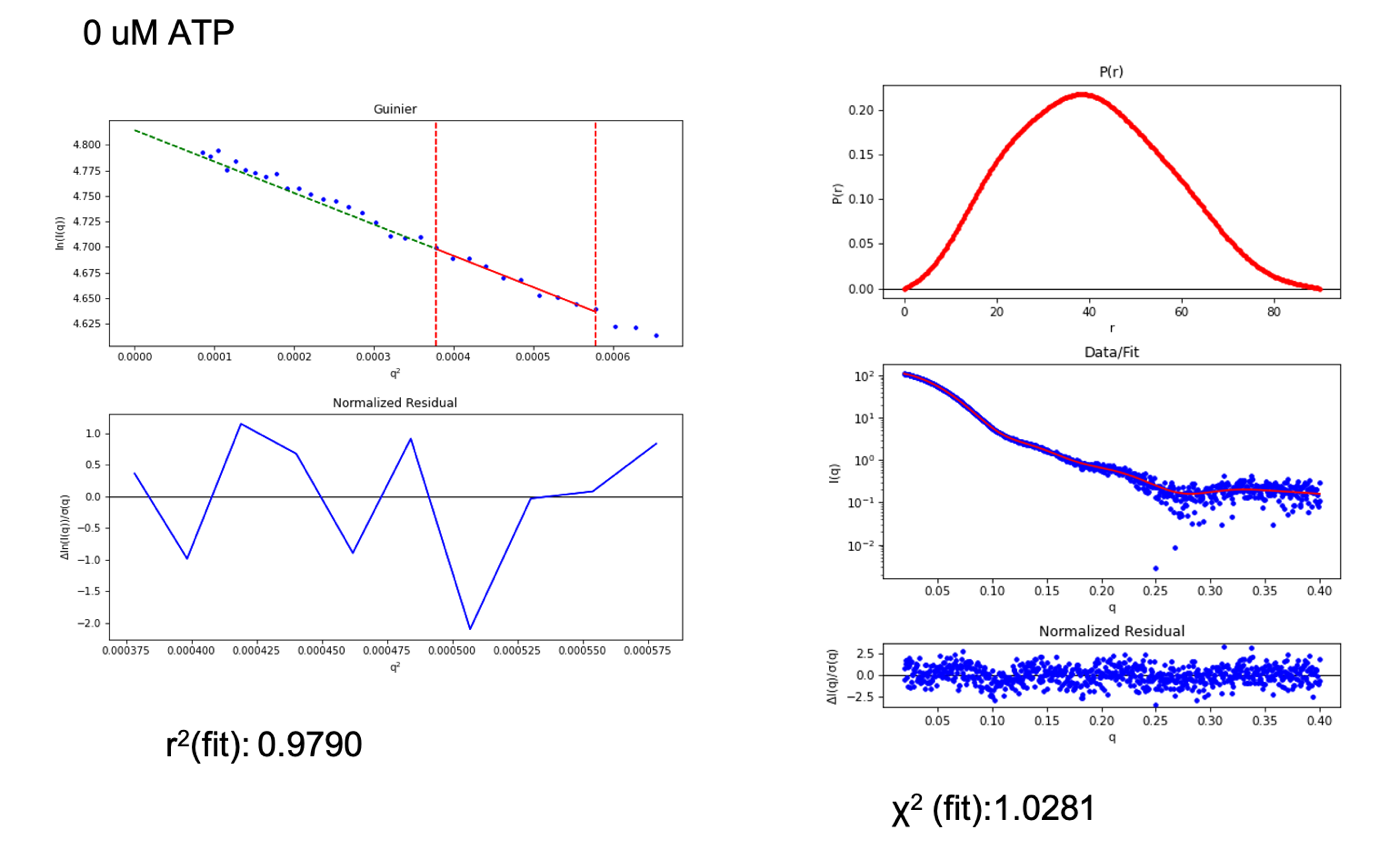


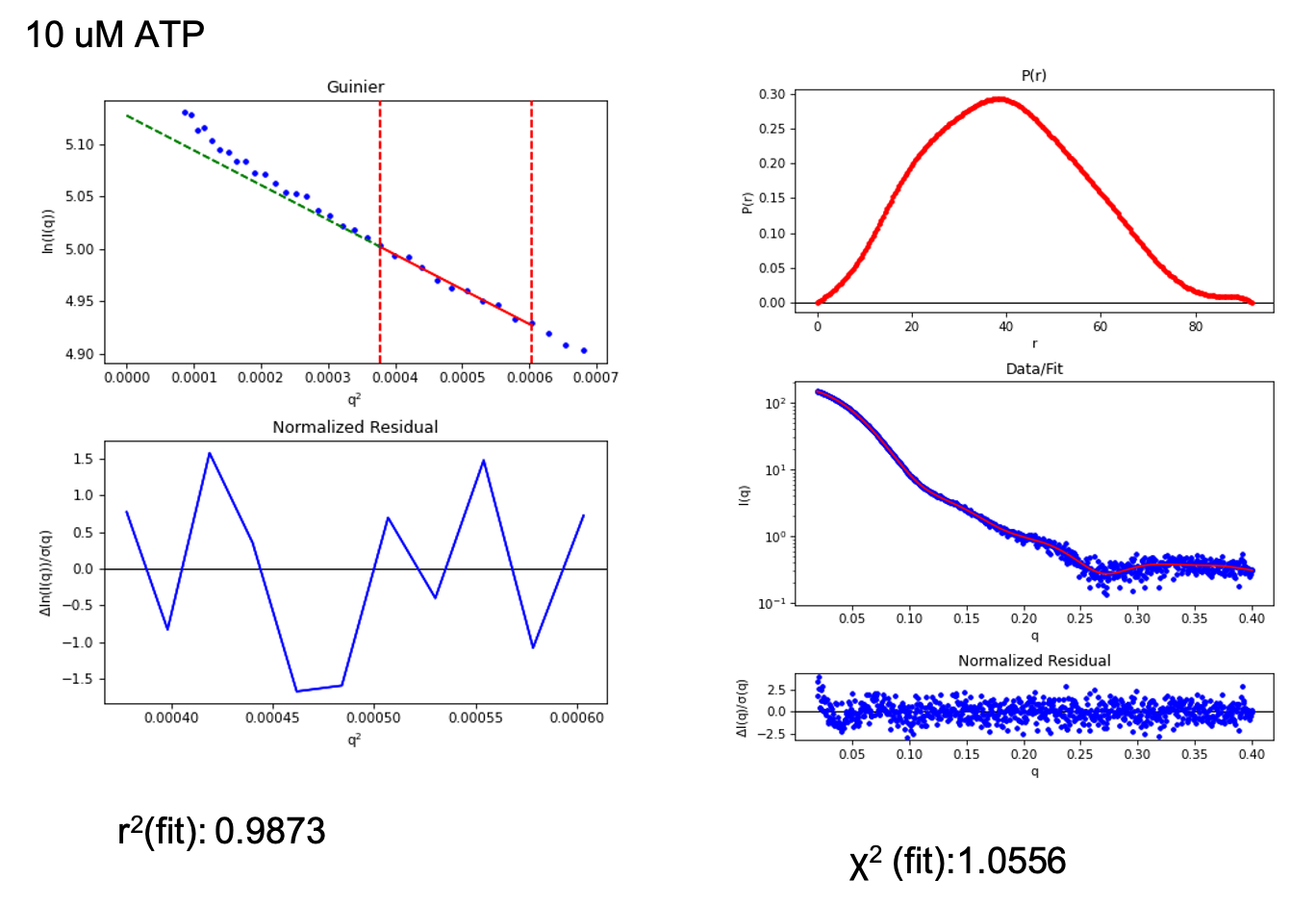


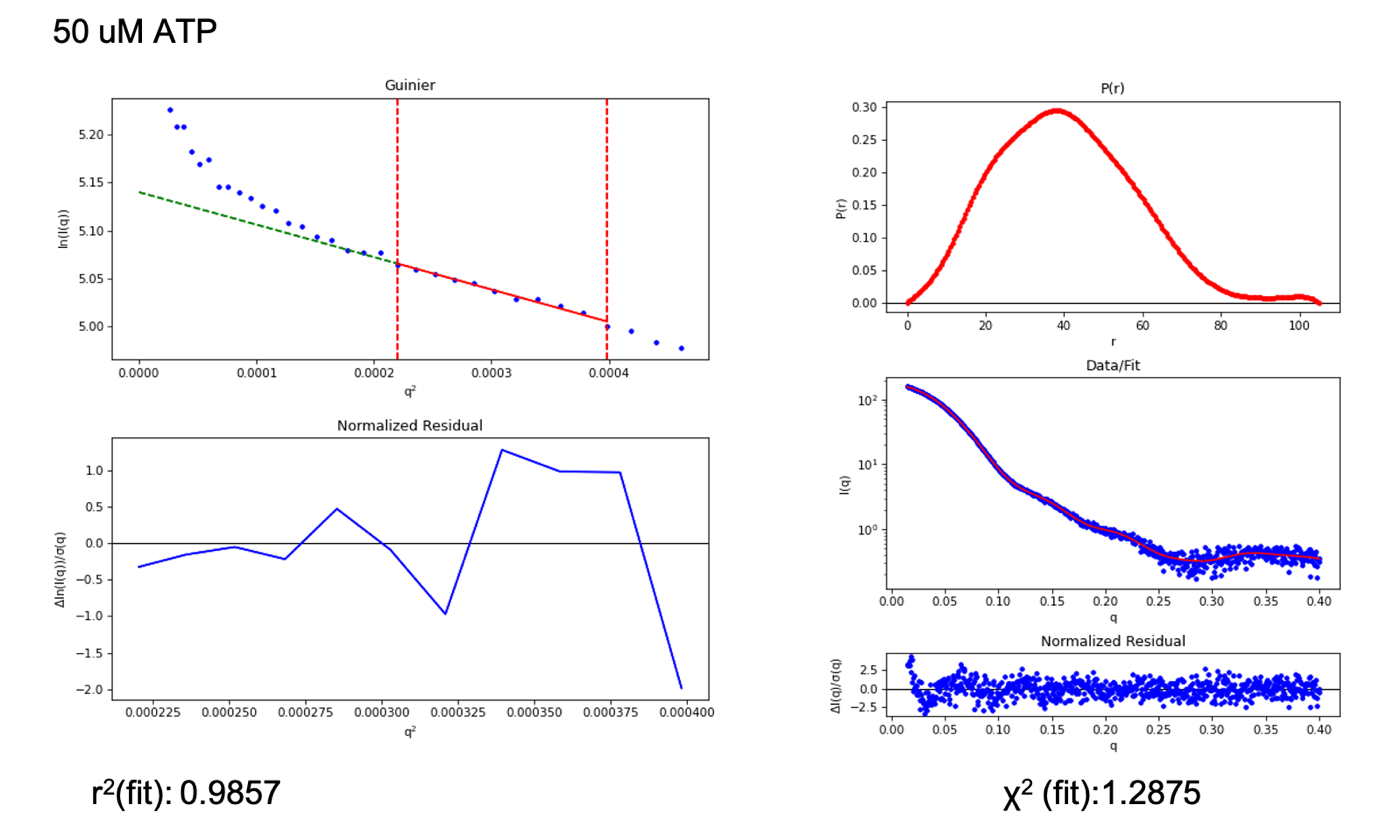


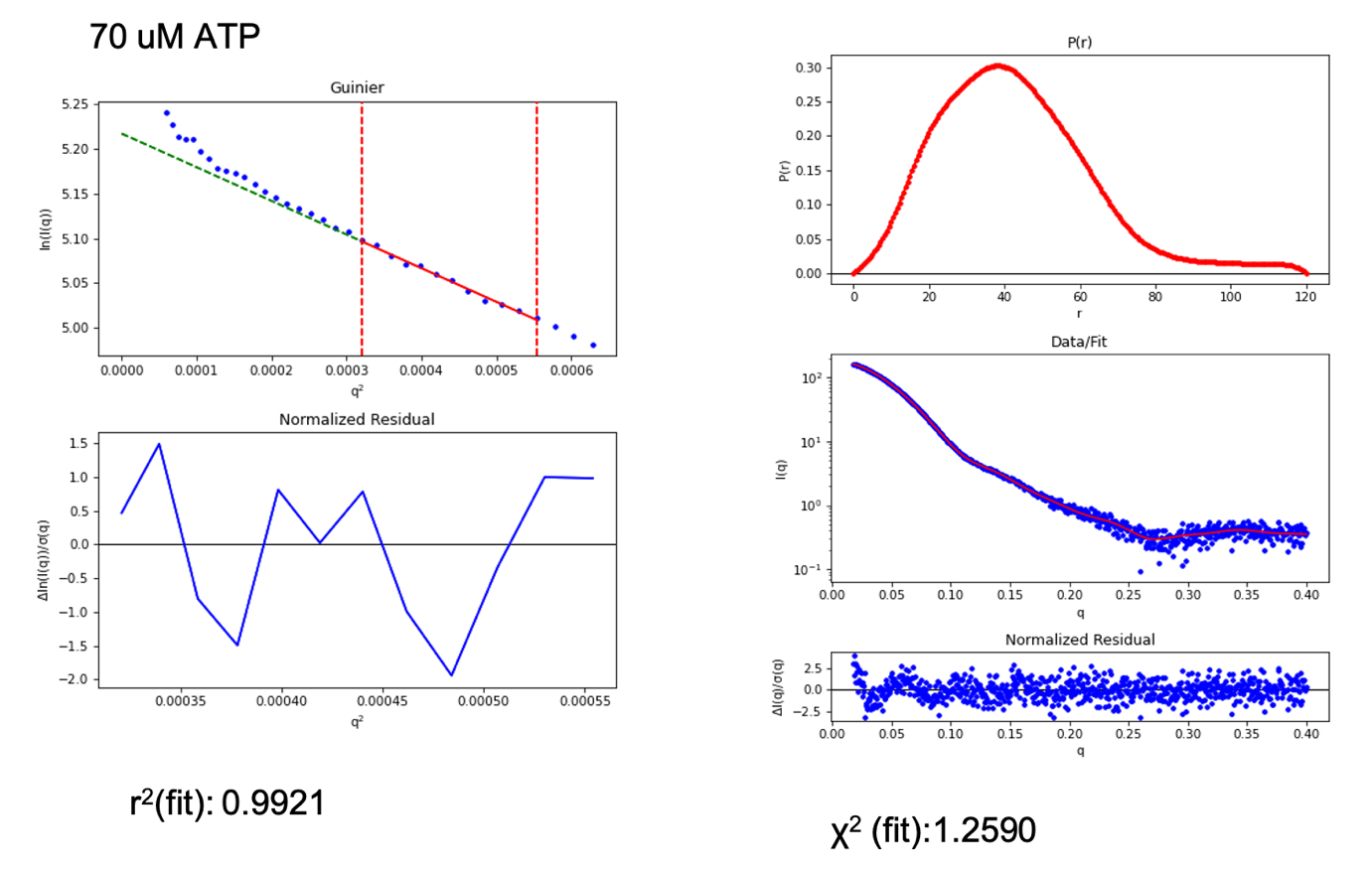


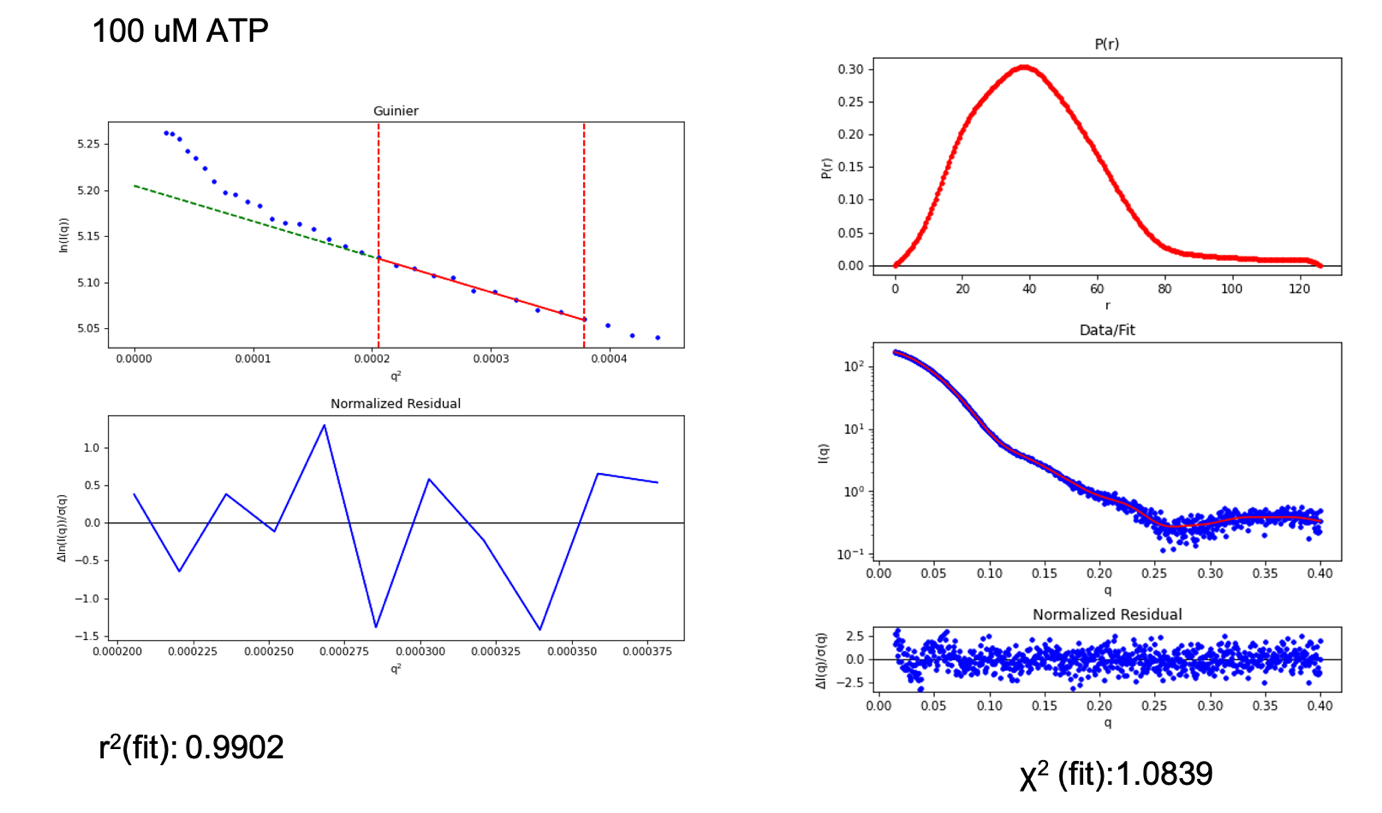


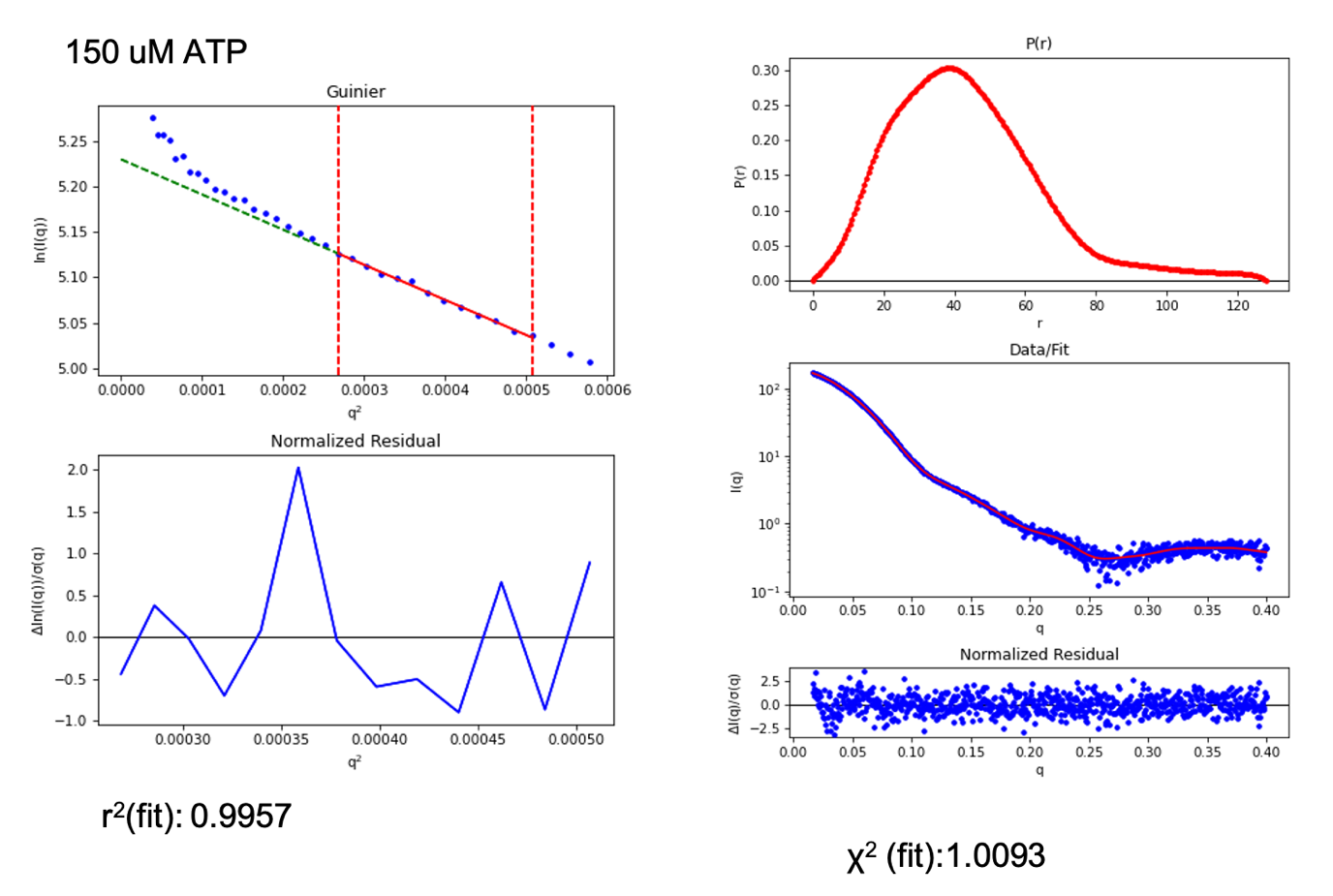


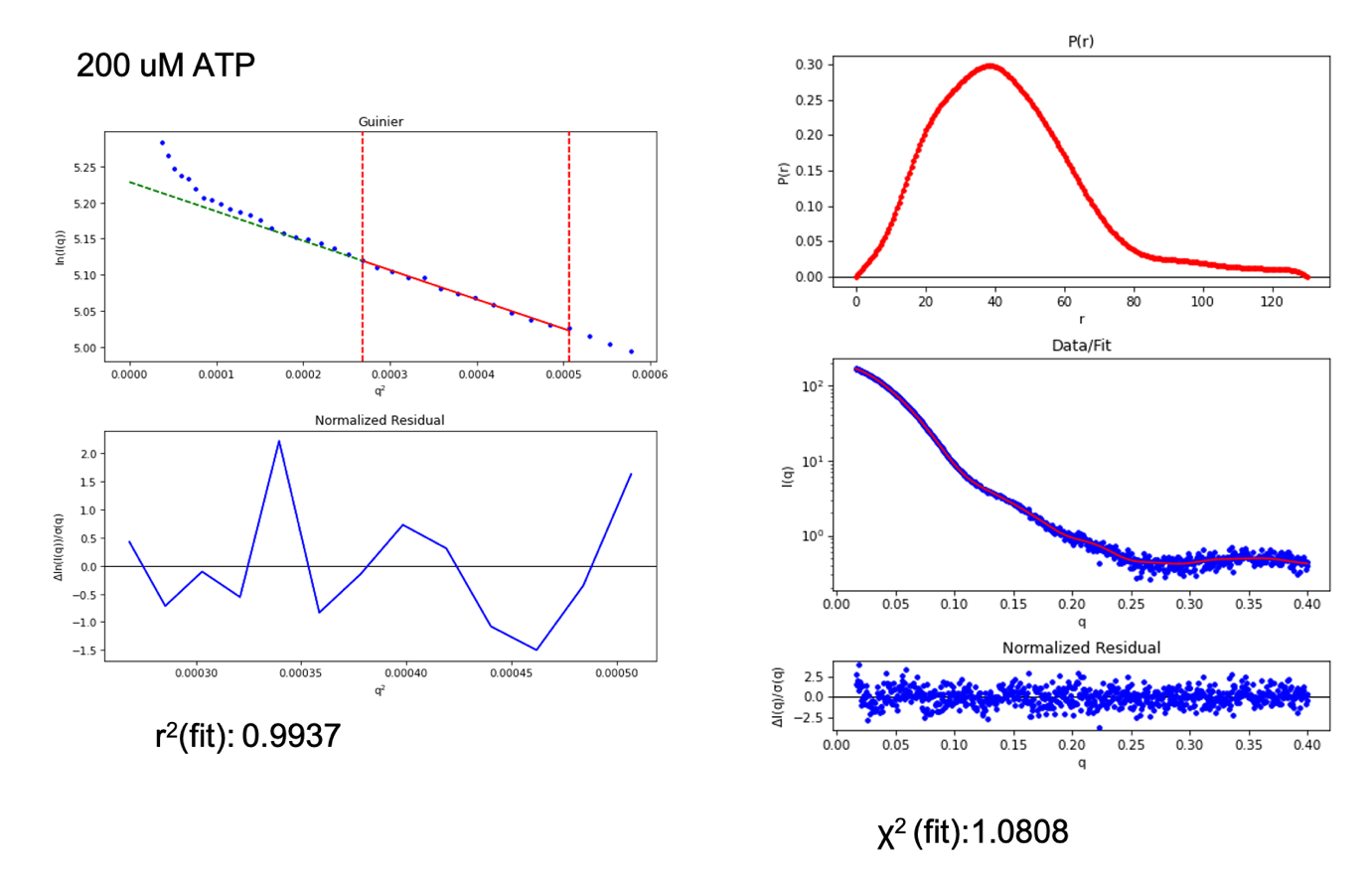


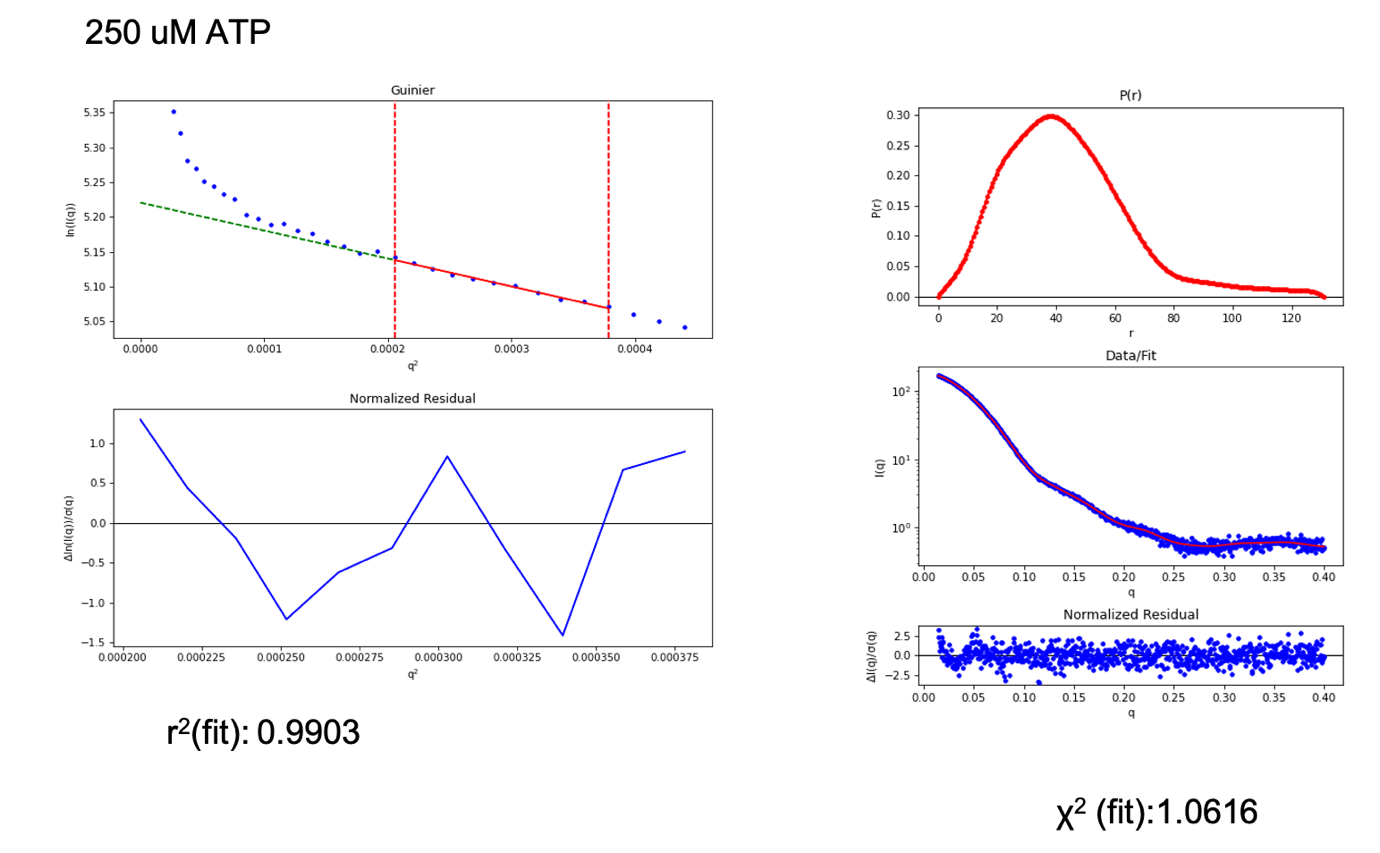


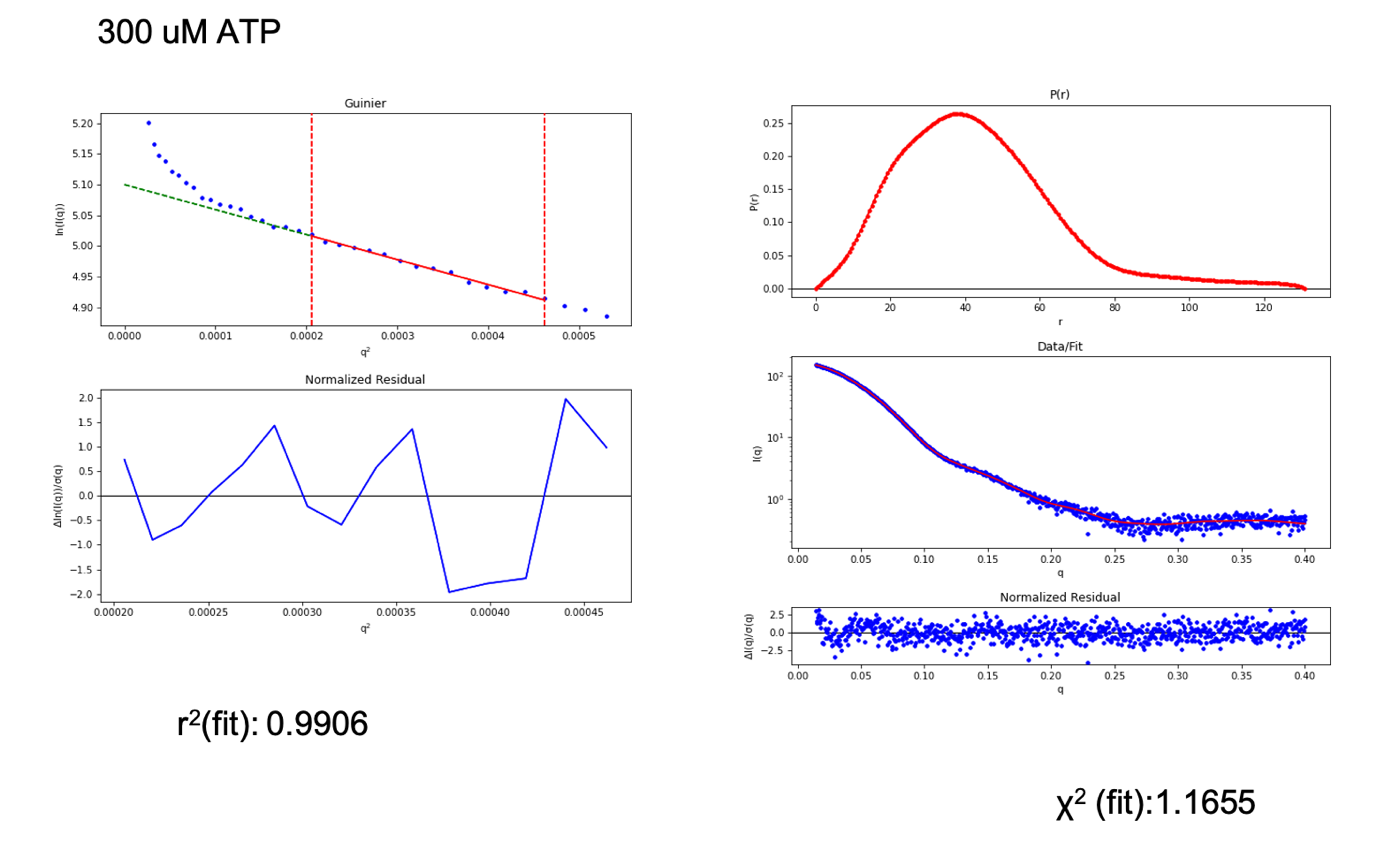


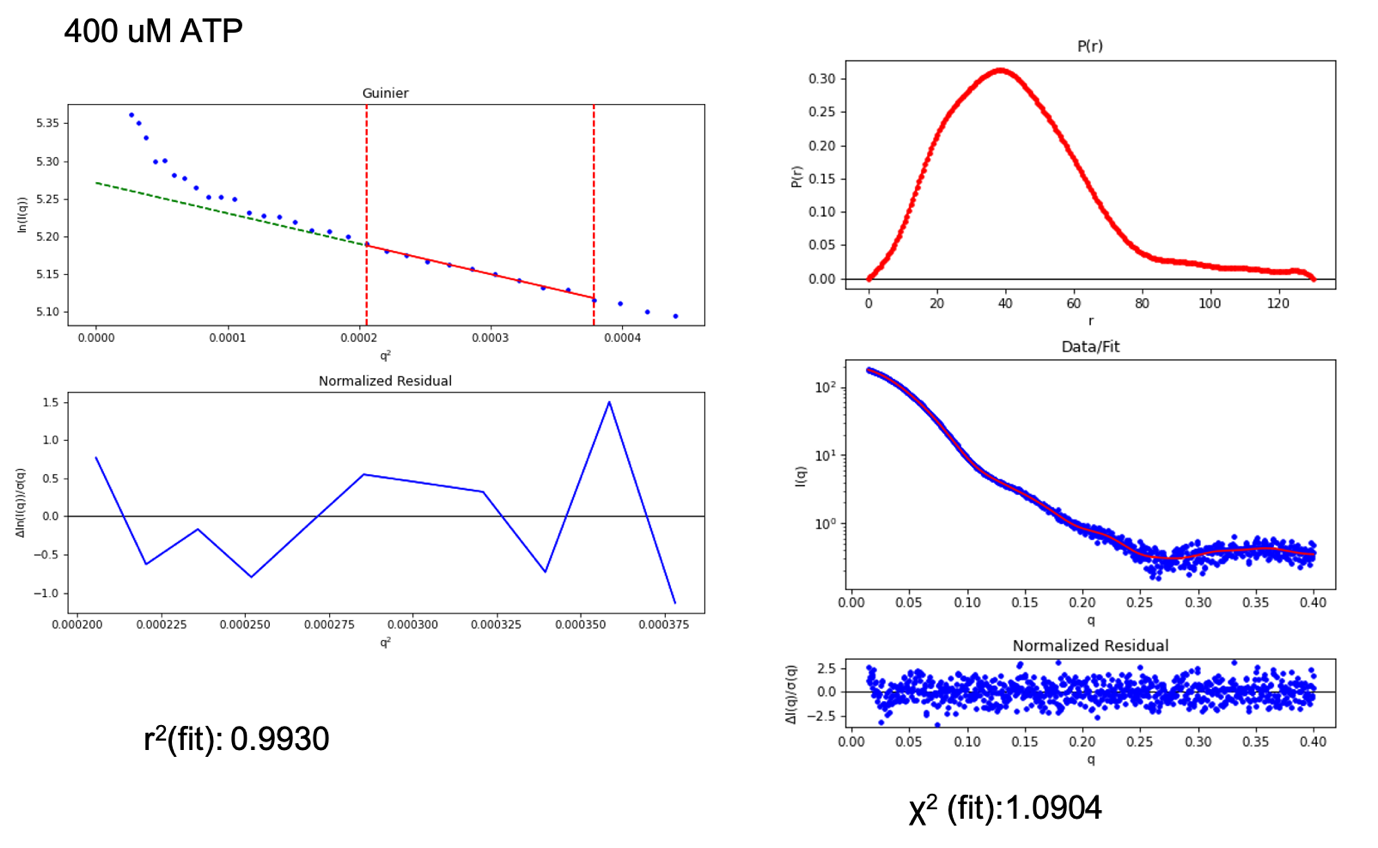


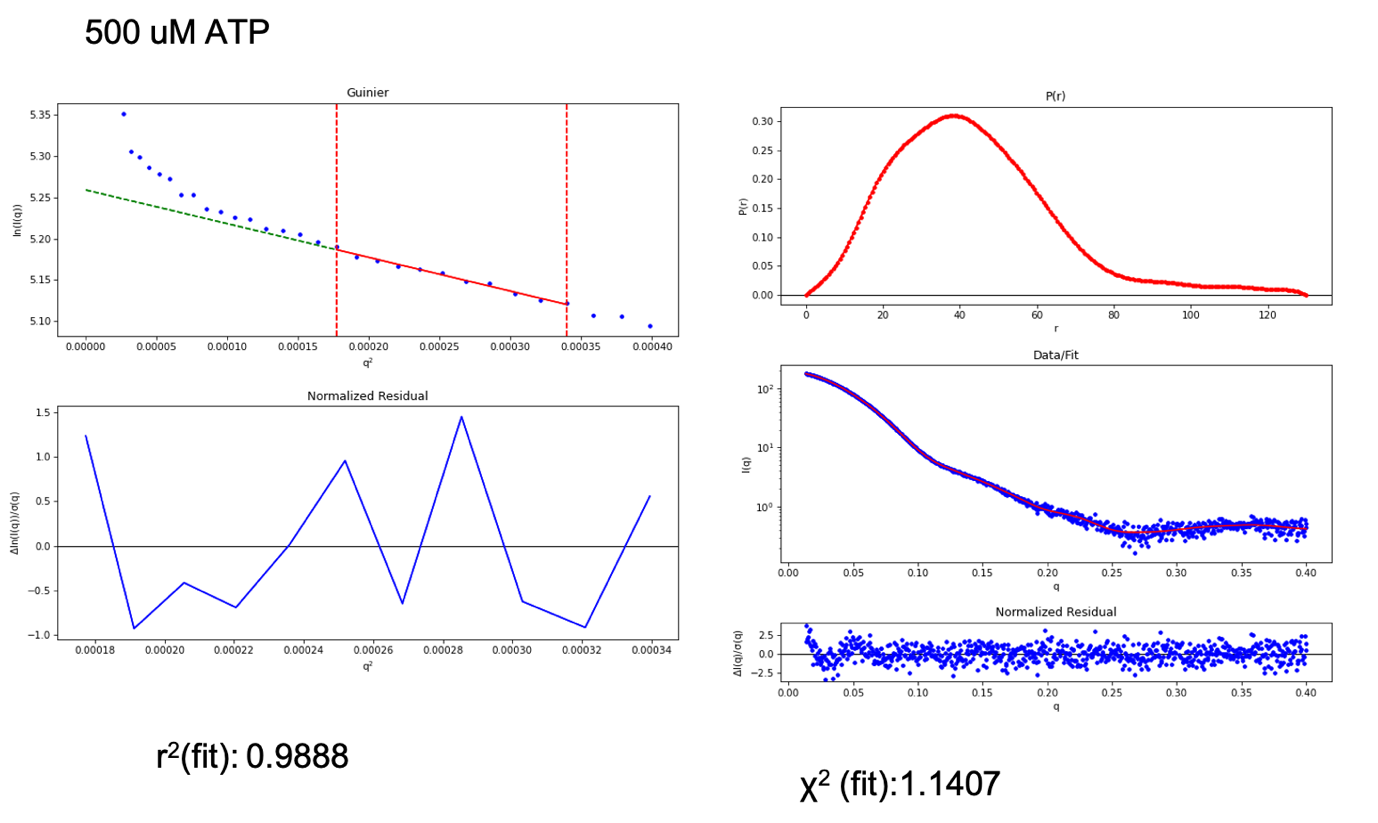


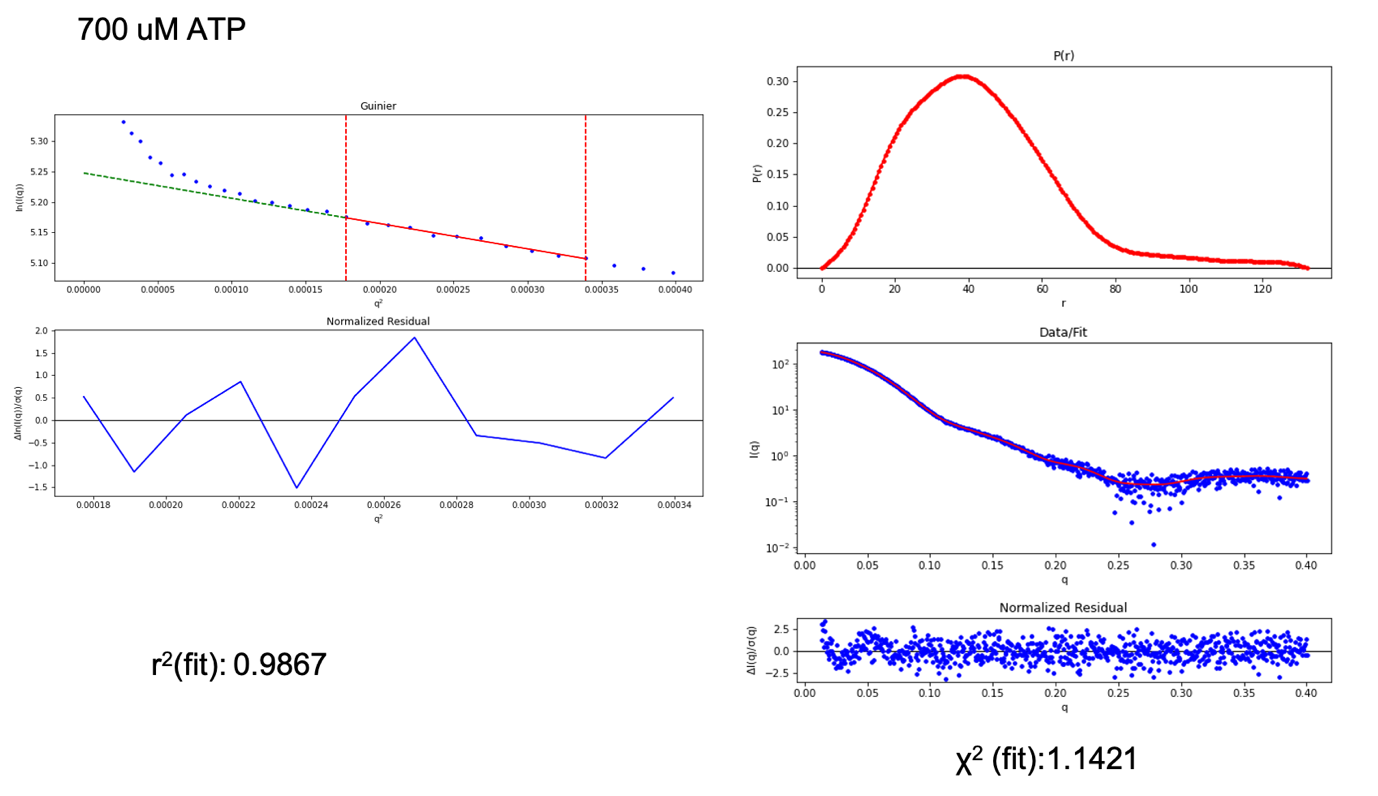


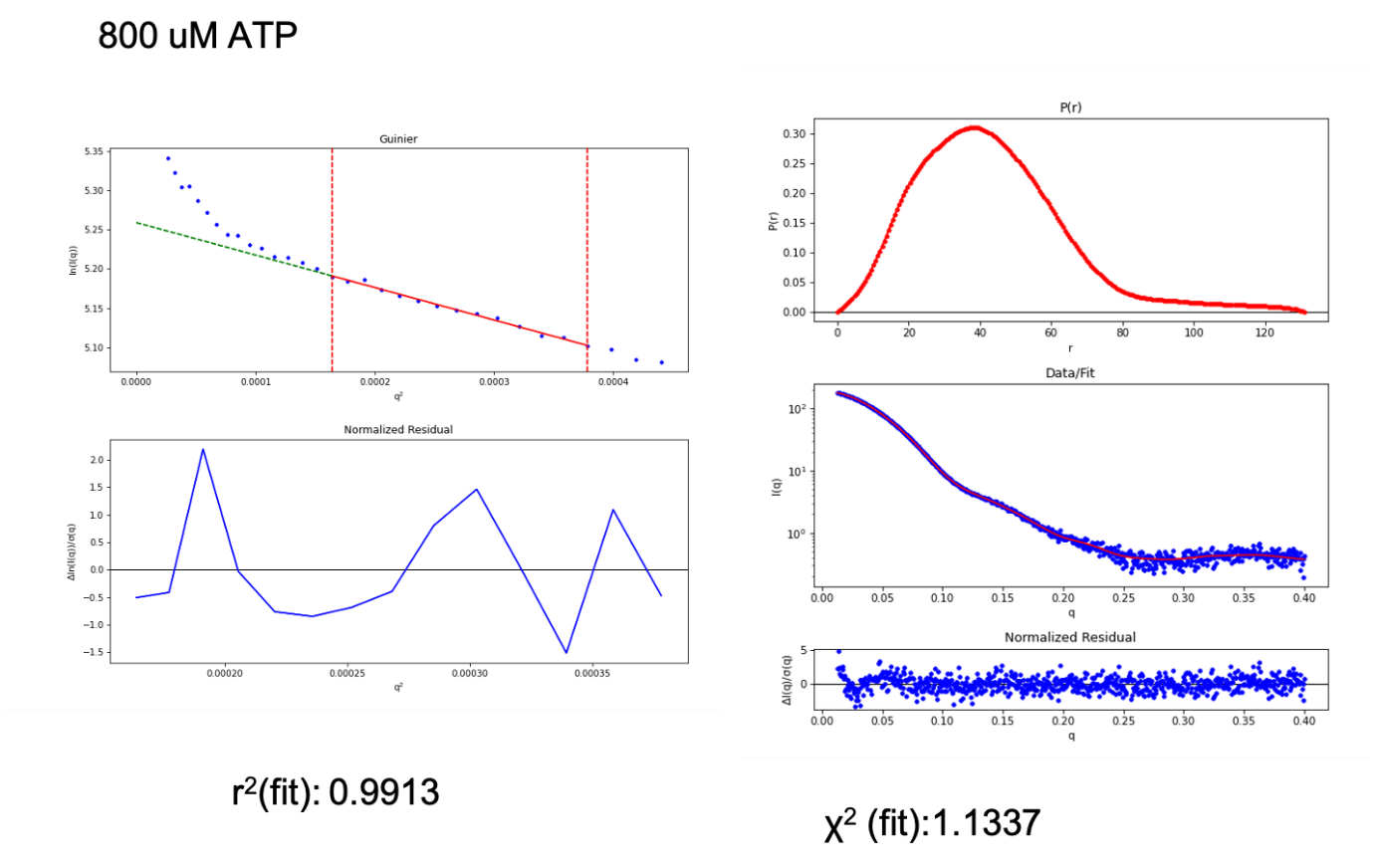


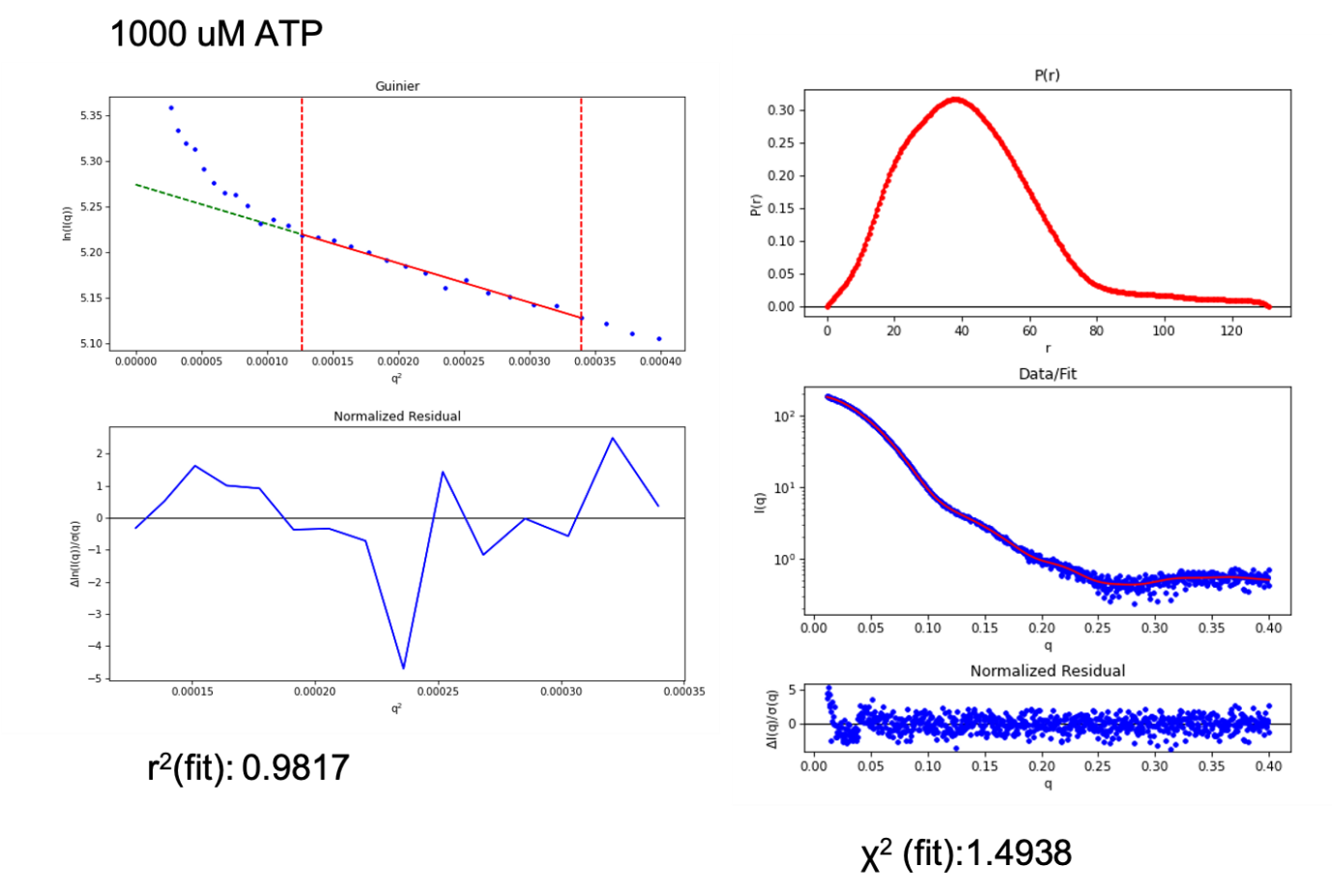


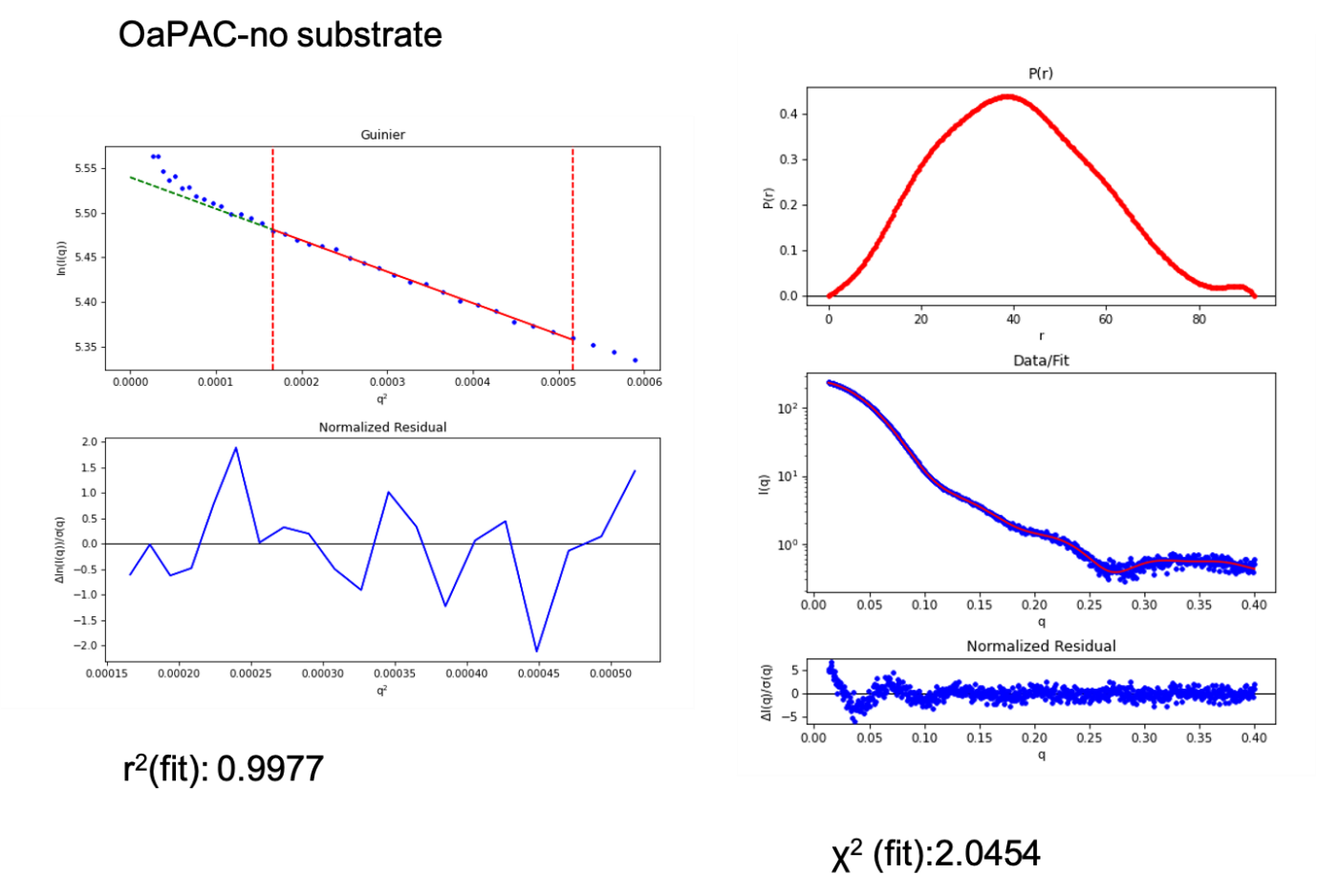


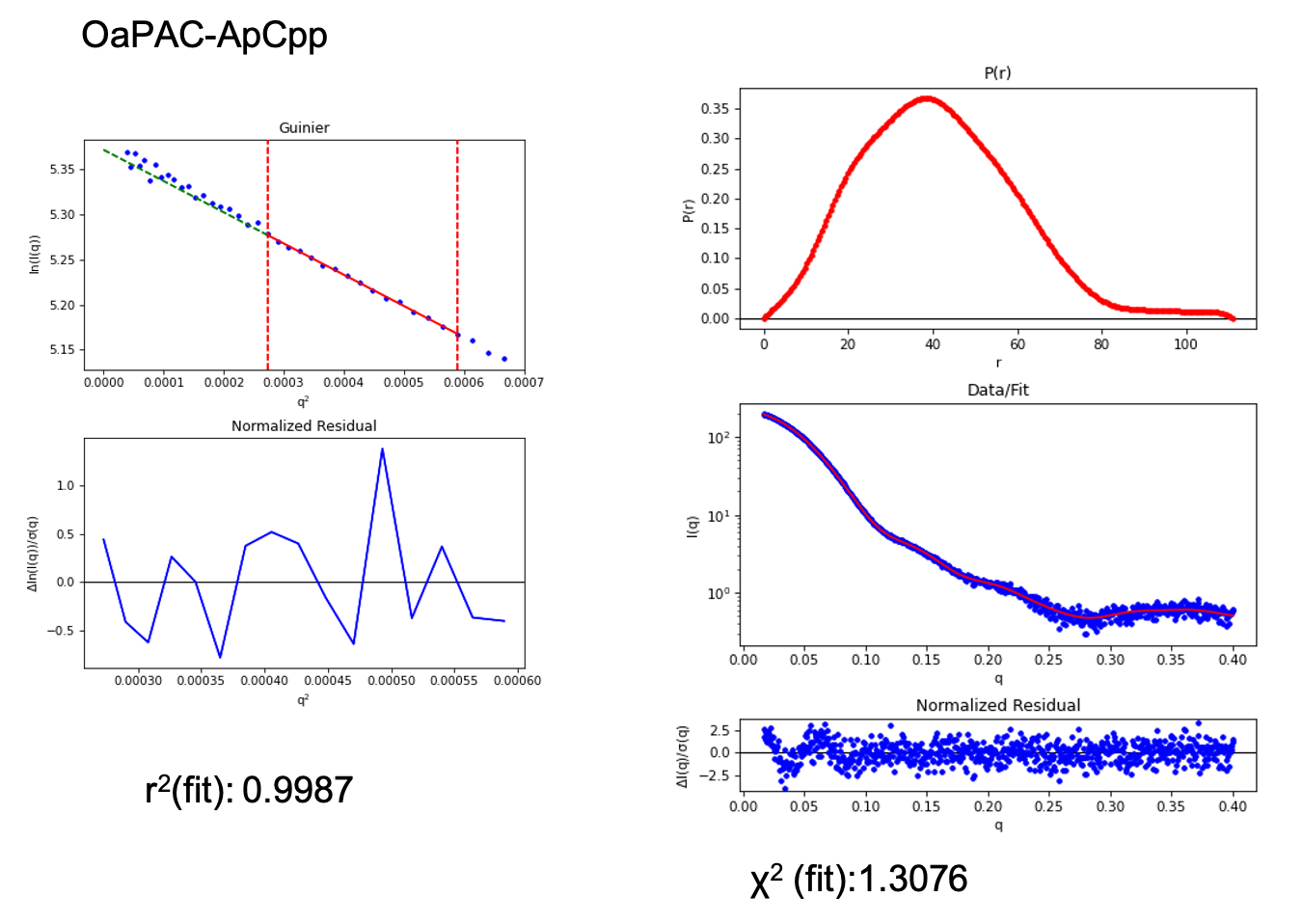


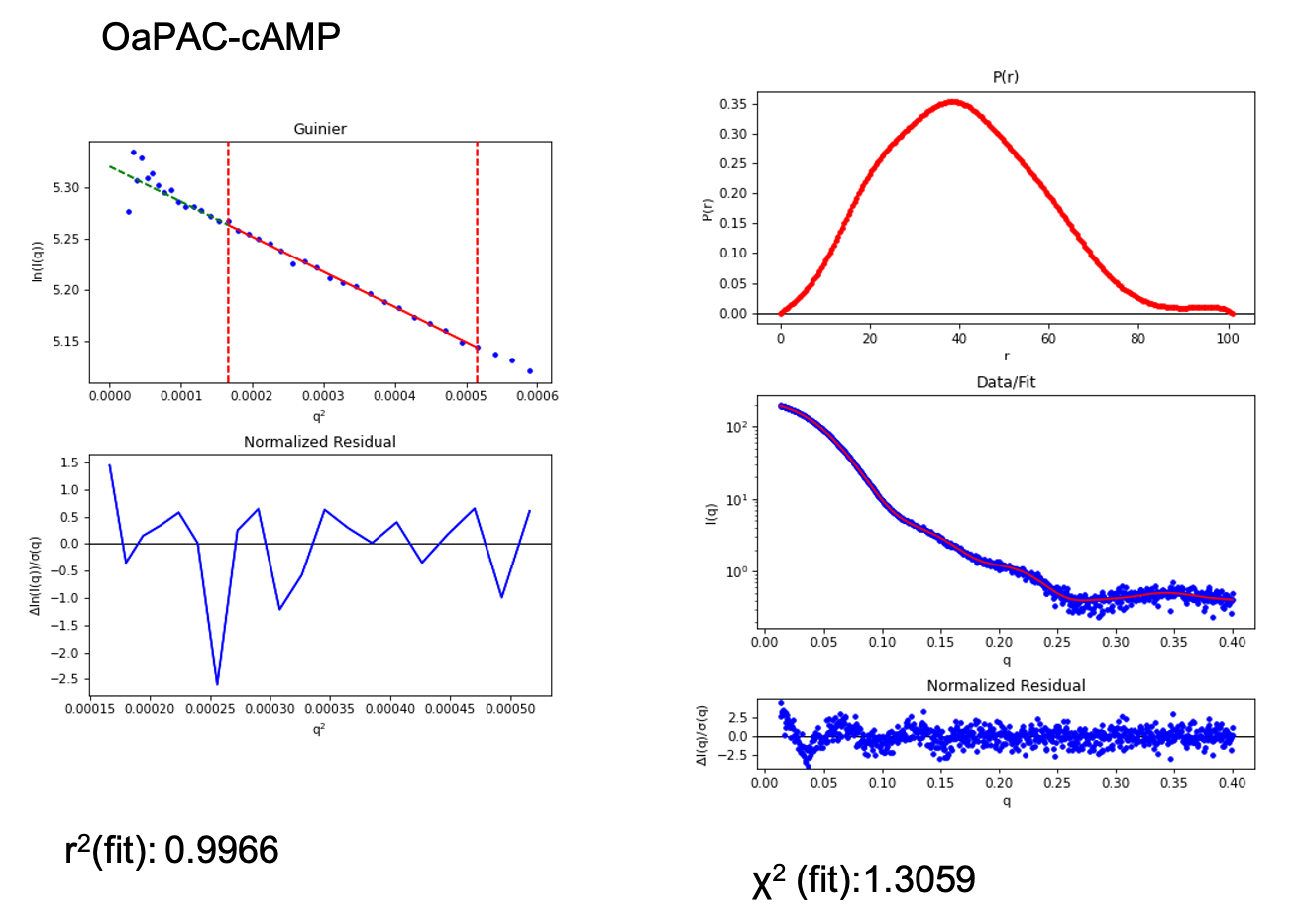


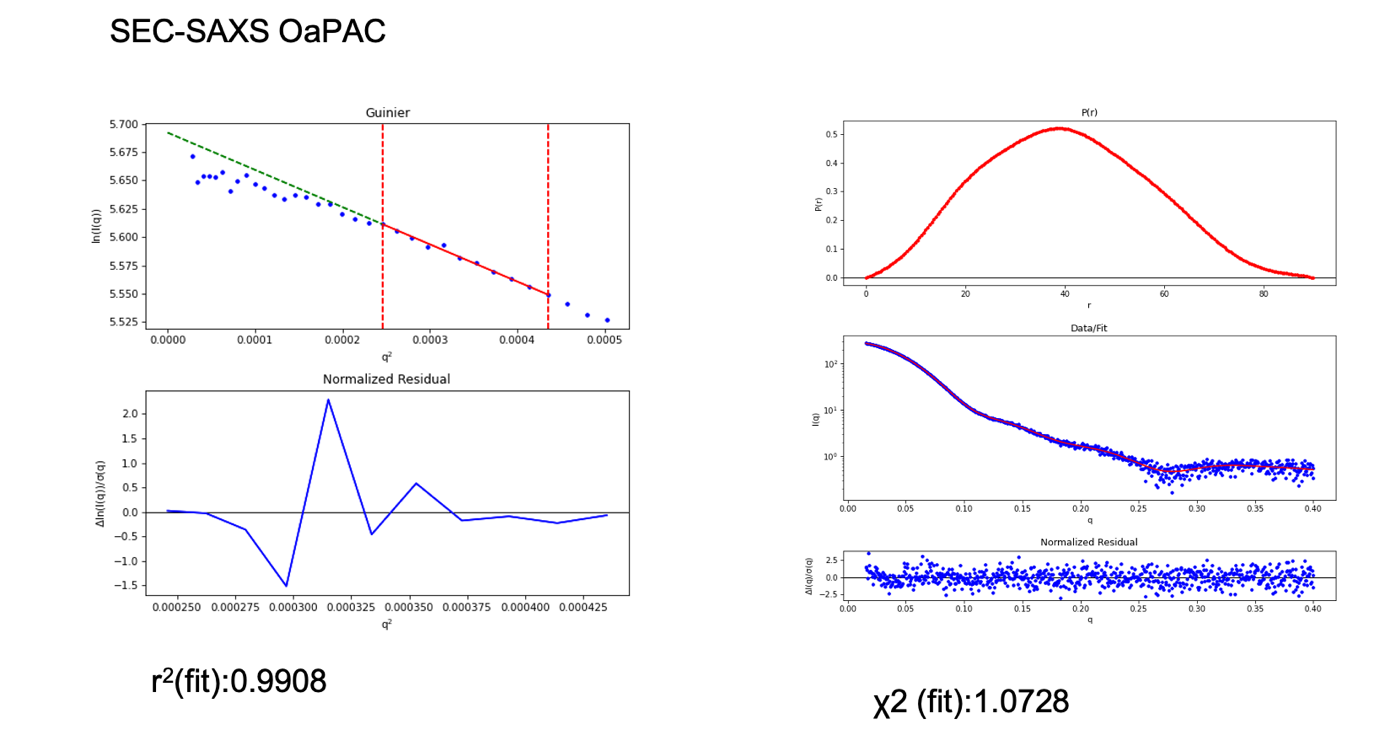


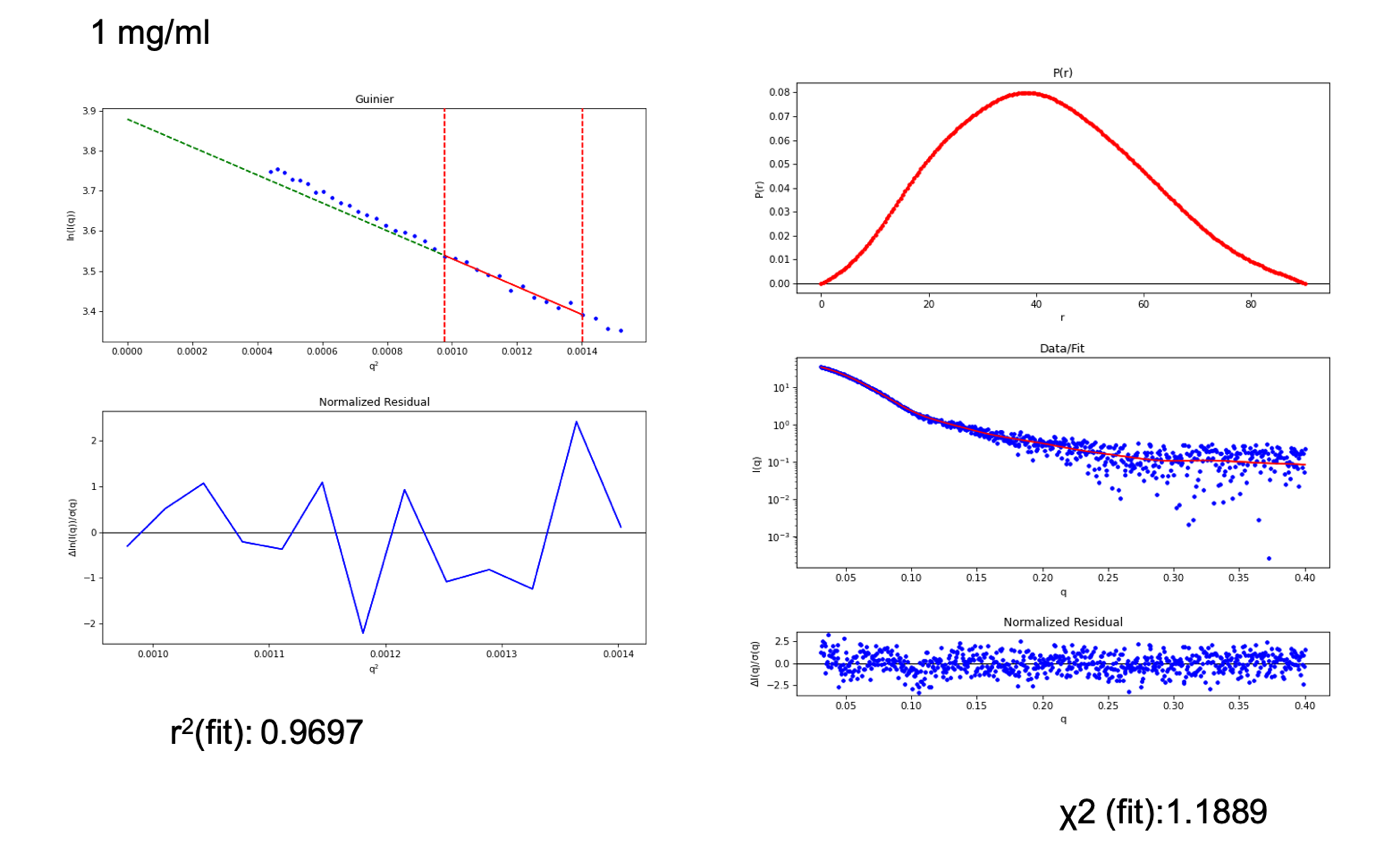


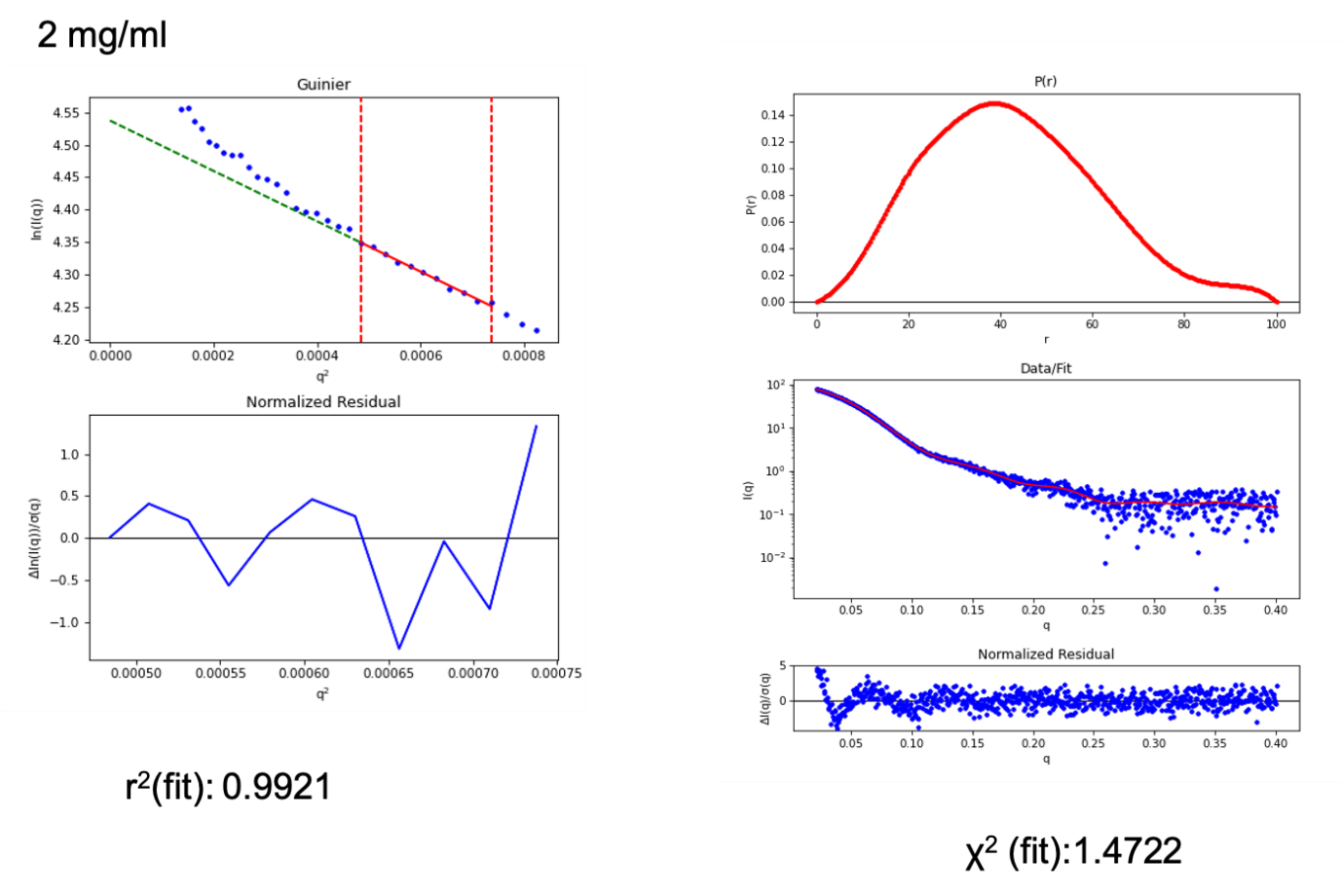


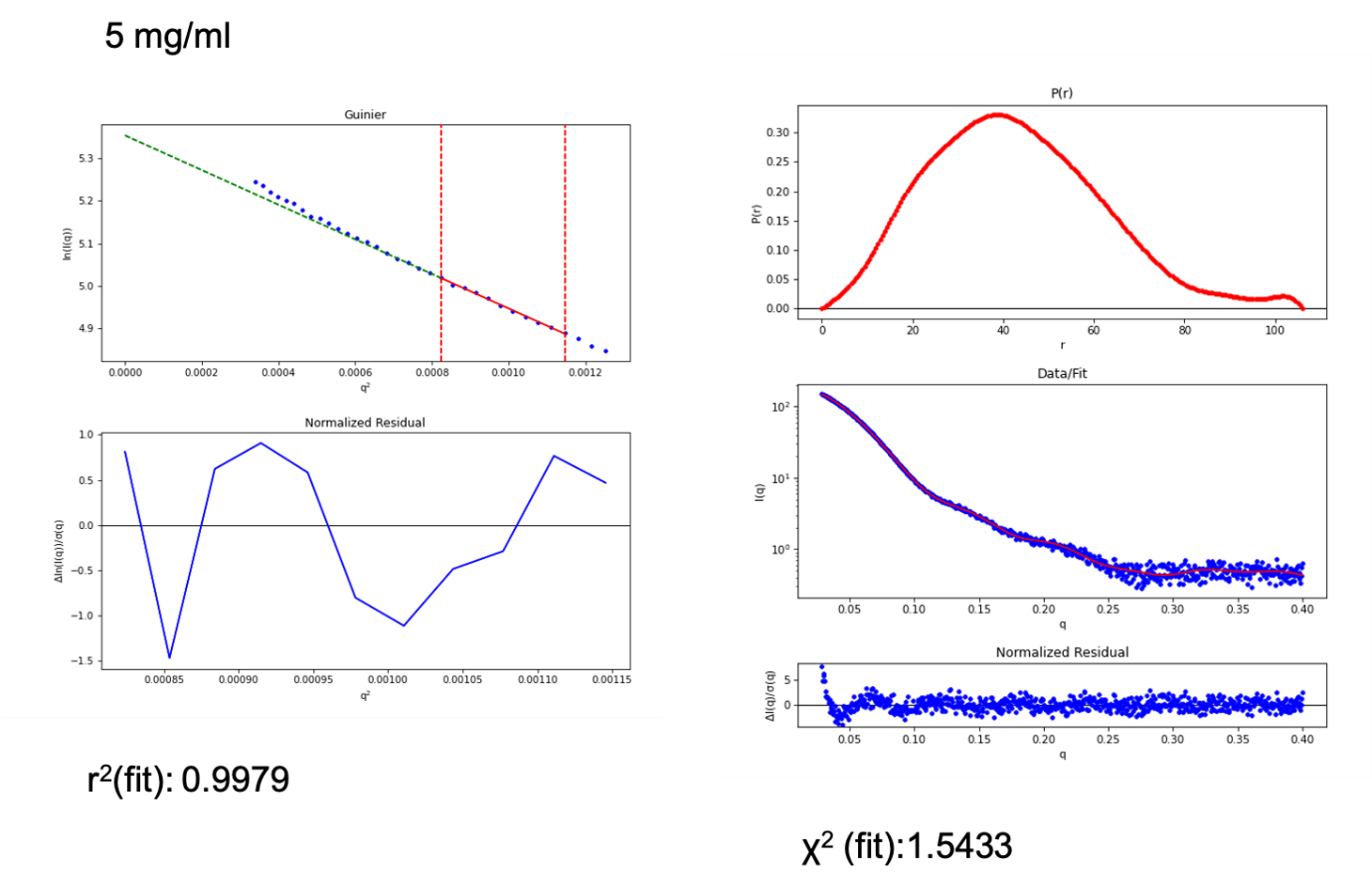


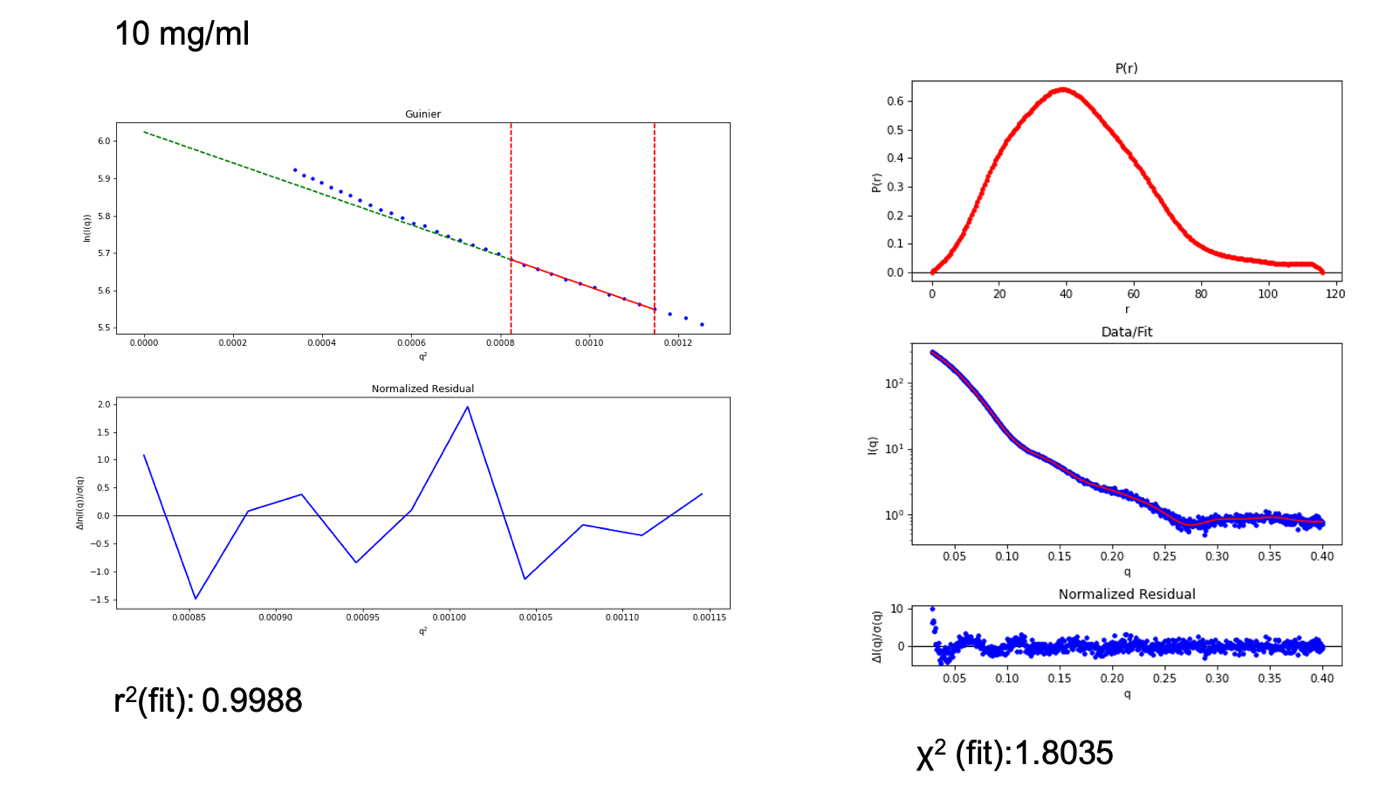


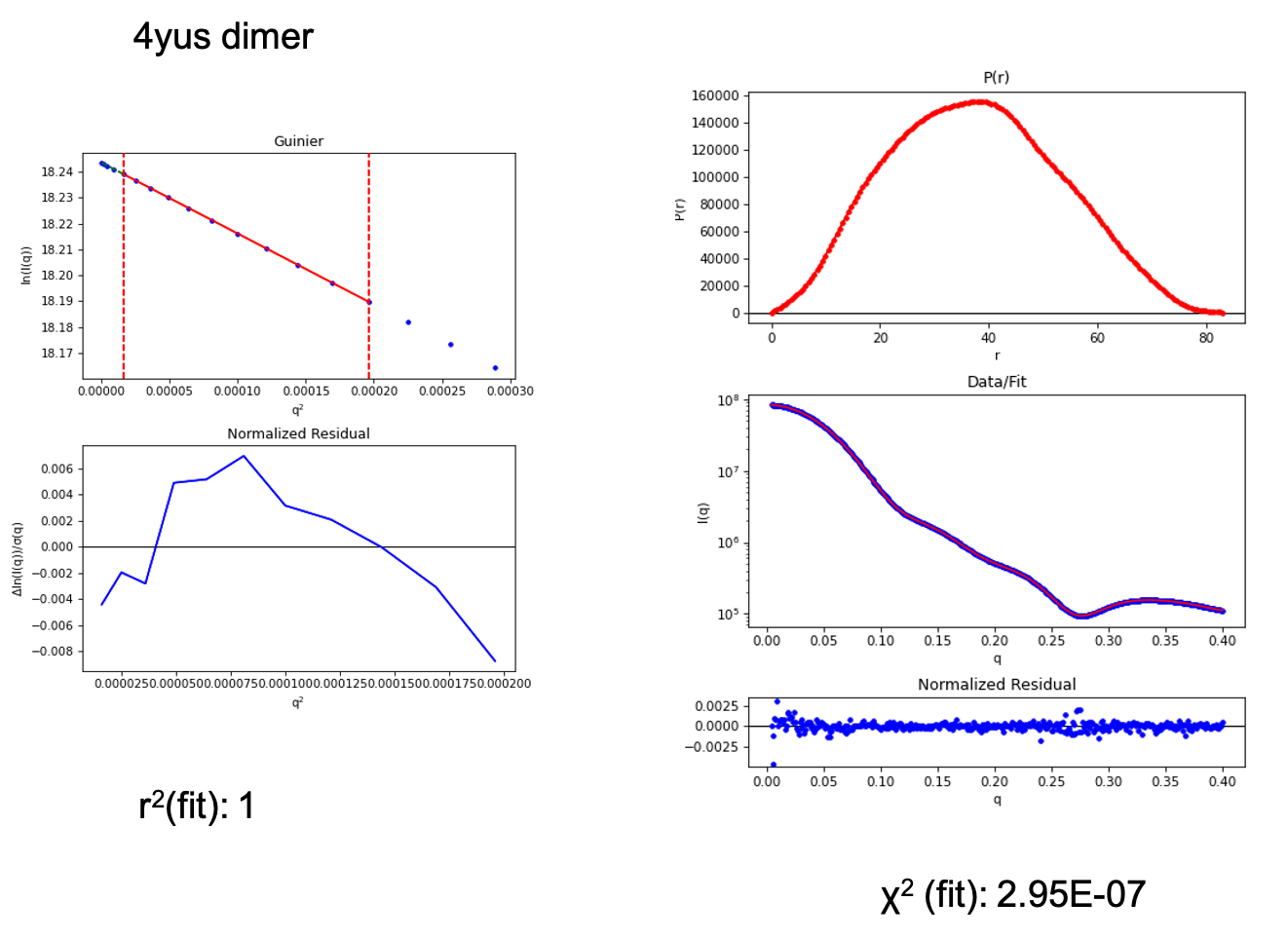


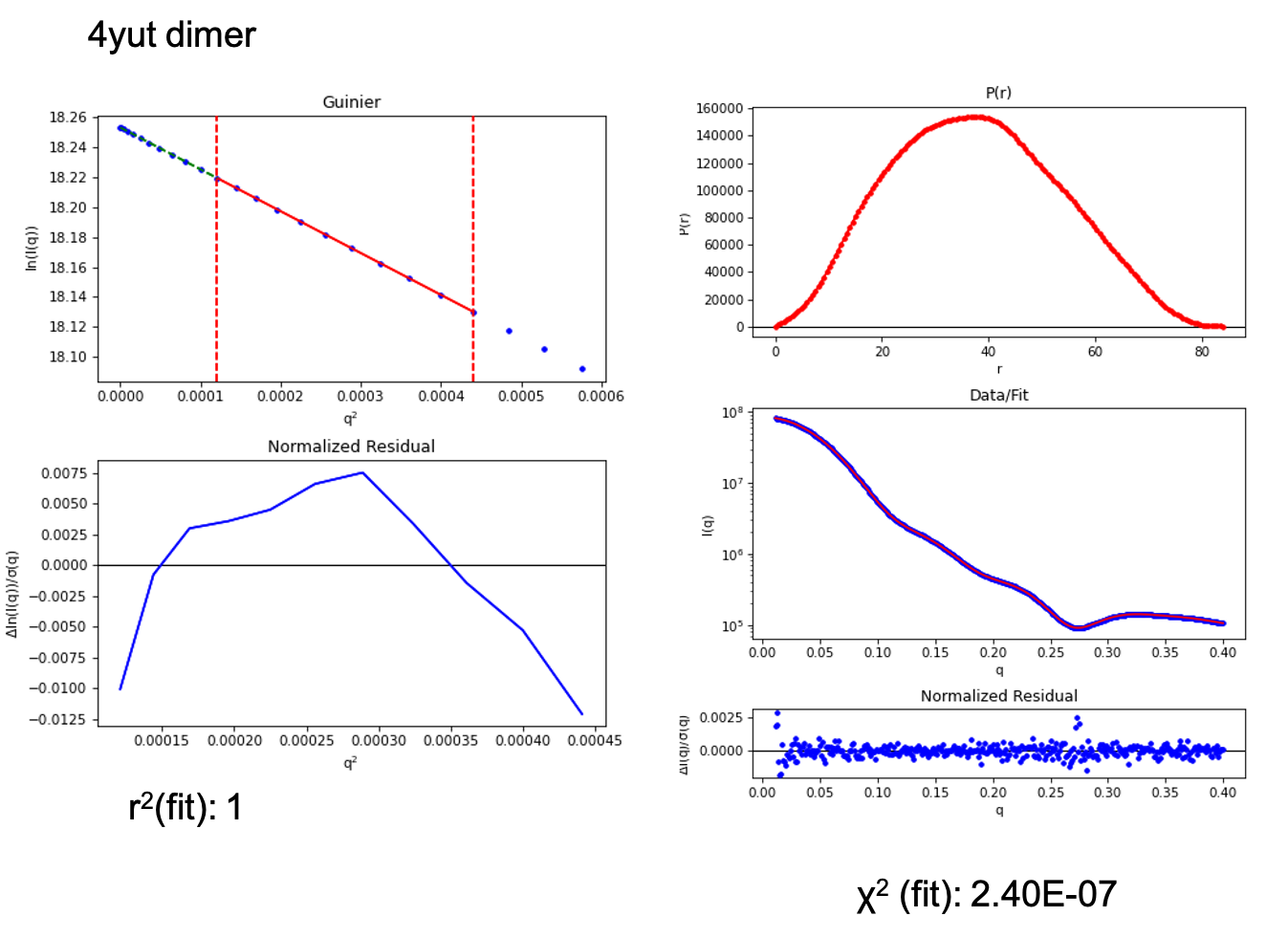


- **Supplementary Note 5: Effect of GuHCl on OaPAC, calculation of the dissociation constant *K*_D_ of mantATP to OaPAC** **and** **circular dichroism spectra of OaPAC and its ATP complex**.


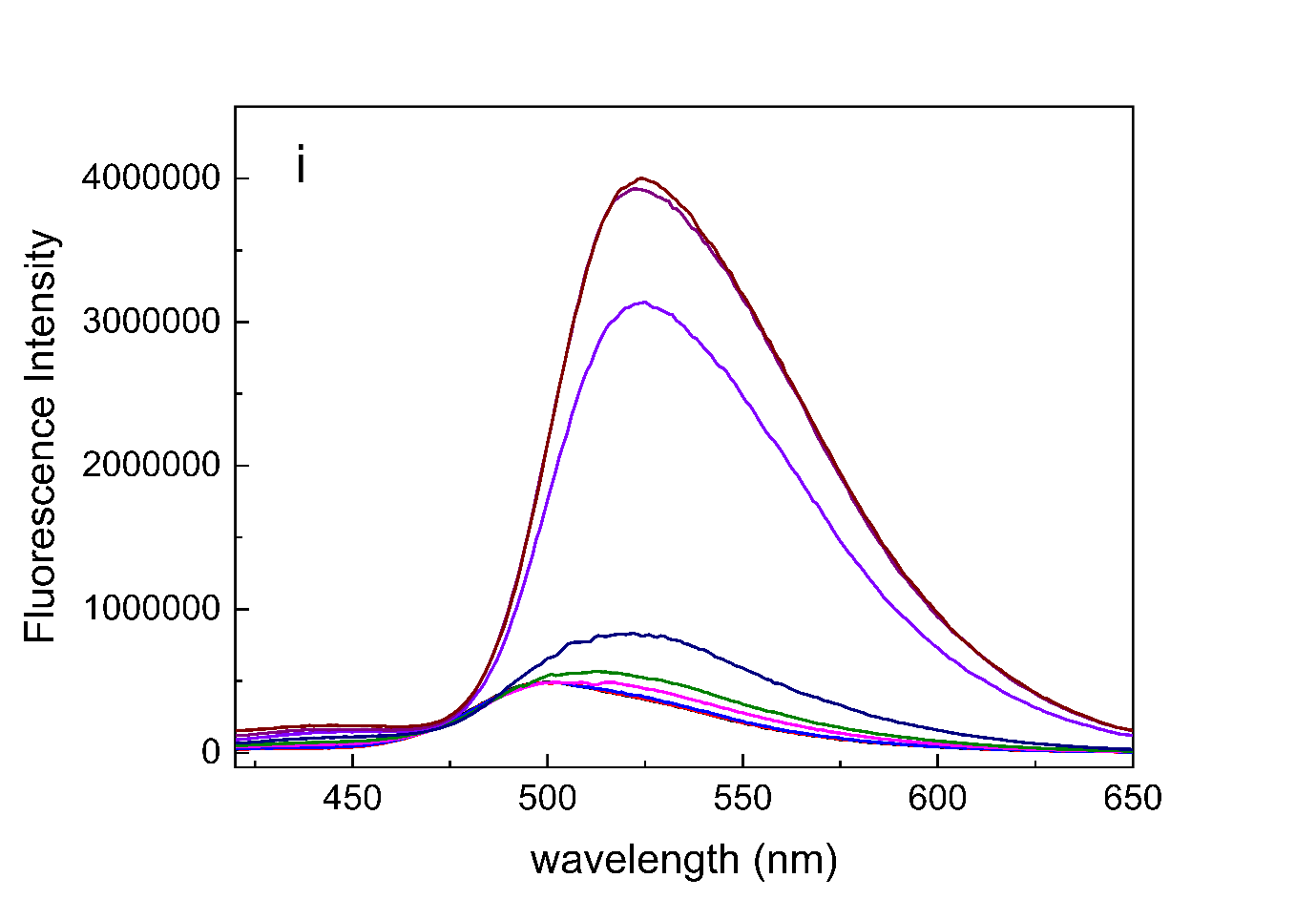


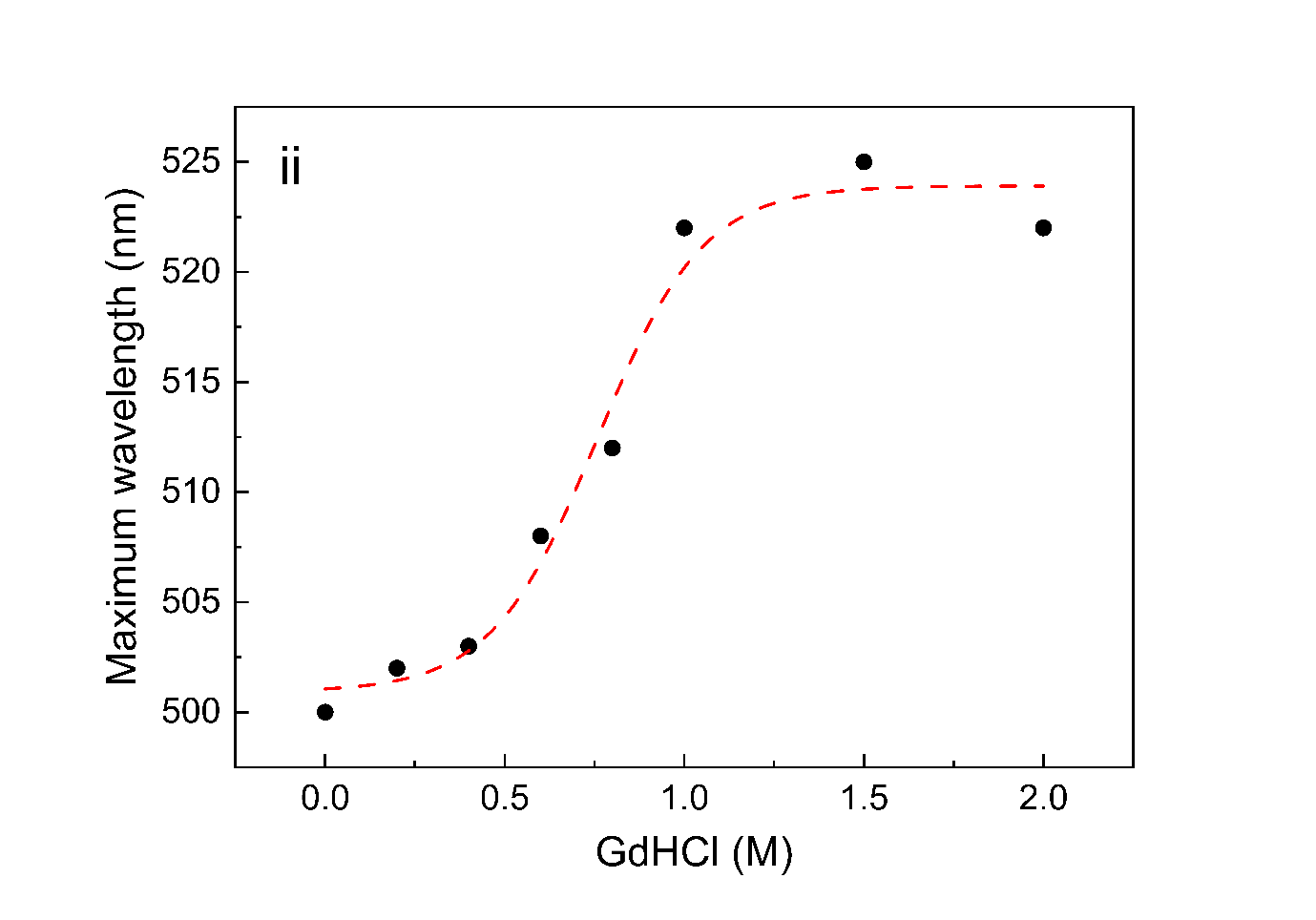


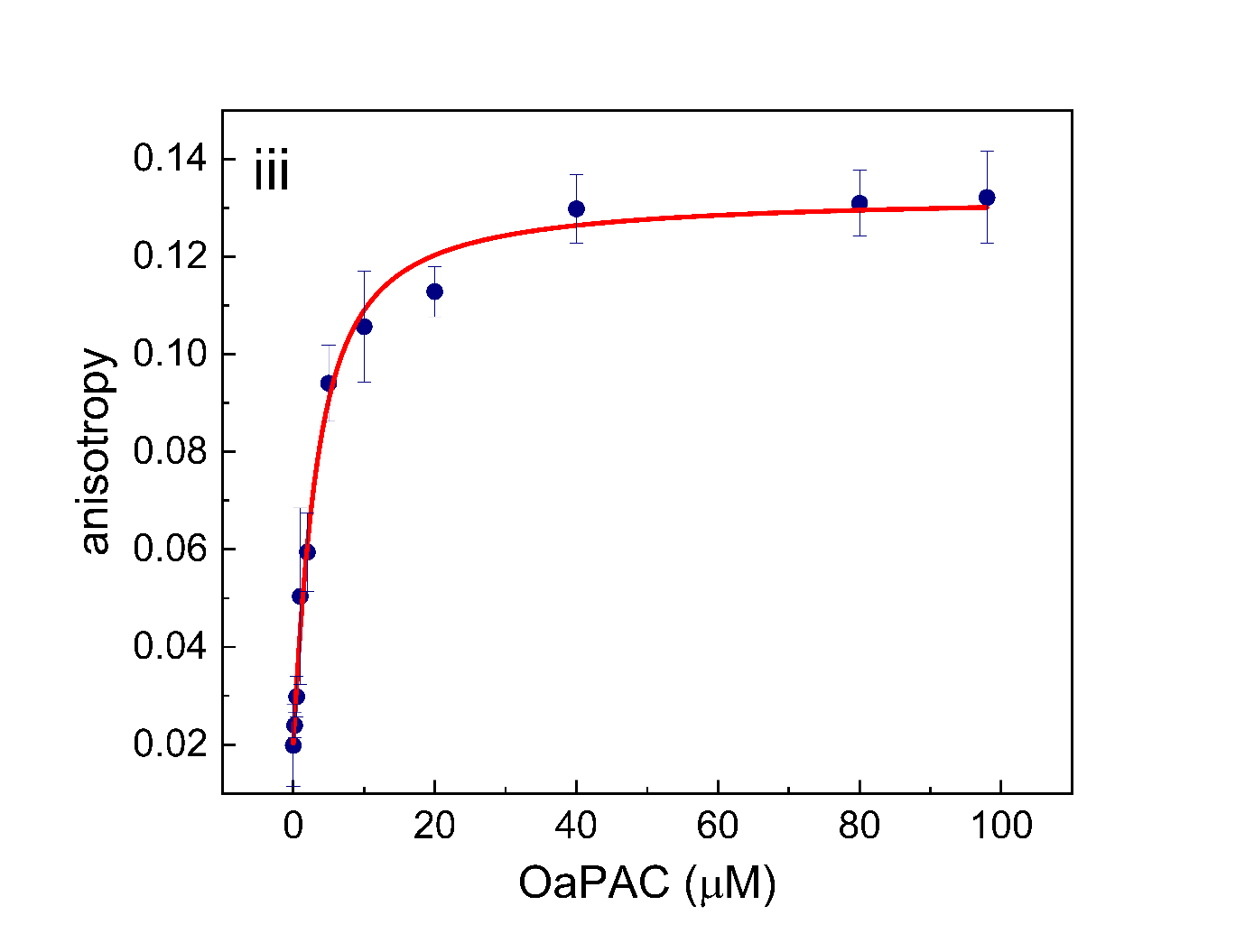


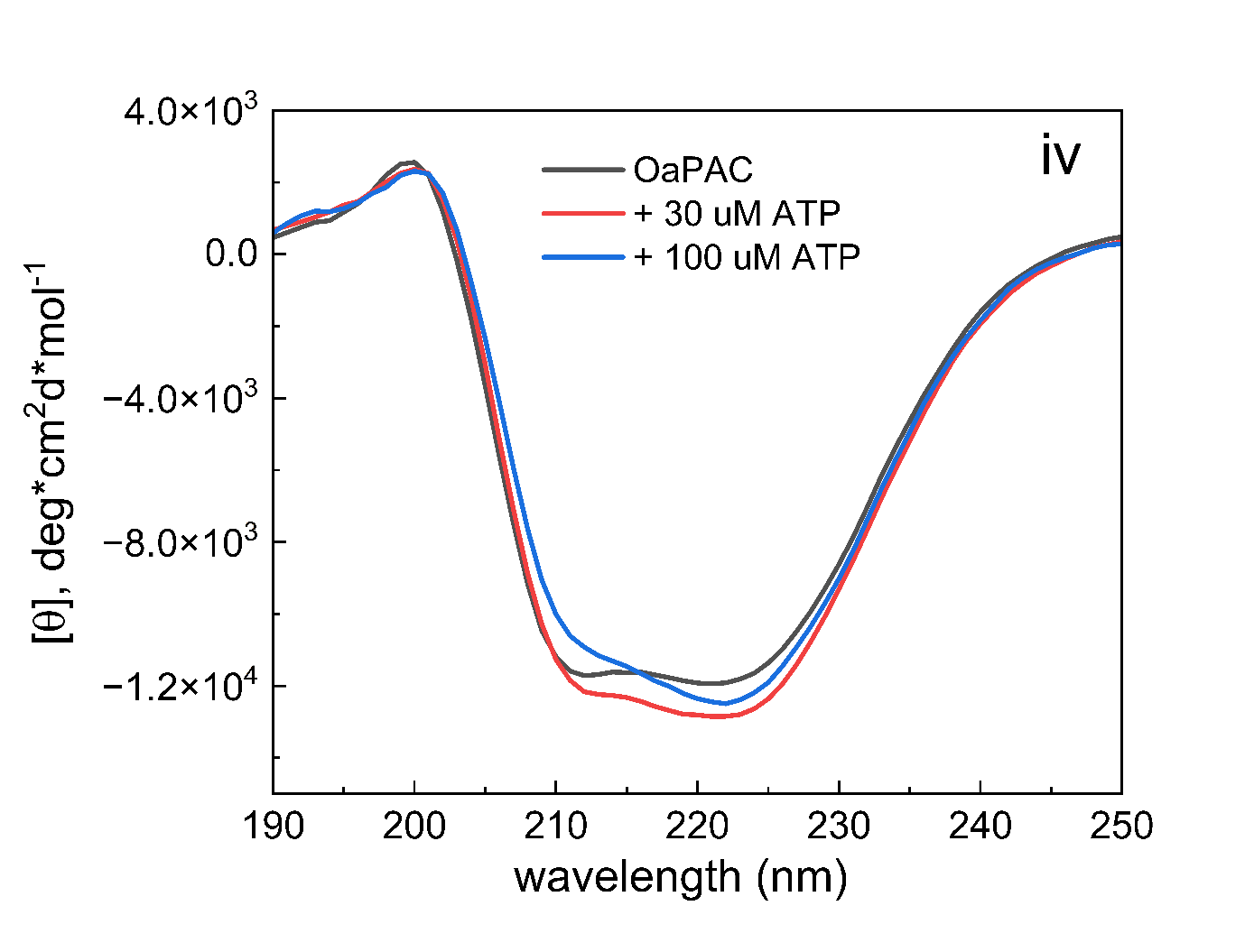


Supplementary Figure 5. i) Emission spectra of OaPAC in the presence of increasing amounts of GuHCl ii) Plot of the emission maximum versus [GuHCl]. iii) Steady-state anisotropy of mantATP (2 μM) upon increasing concentration of OaPAC. Fitting to the binding curve is shown by solid lines [mantATP: *K*_D_= 2.25 ± 0.4 μM (n=3)]. Error bars represent standard deviation iv) CD spectra of OaPAC and its complex with ATP at two different concentrations (OaPAC: black line, OaPAC +30 μΜ ATP: red line, OaPAC +100 μΜ ATP: blue line).

**Supplementary Note 6: The conversion of ATP to cAMP by OaPAC is negligible in the dark.**

An enzymatic assay was employed to measure the enzymatic activity of OaPAC in the dark- and light-adapted states as described in ref 42. Briefly, a spectrophotometric assay that detects pyrophosphate released by OaPAC upon conversion of ATP to cAMP was used. The inorganic pyrophosphatase enzyme converts pyrophosphate into two equivalents of phosphate which is then consumed by the 2-amino-6-mercapto-7-methylpurine riboside/purine nucleoside phosphorylase reaction and detected by an increase in absorbance at 360 nm.


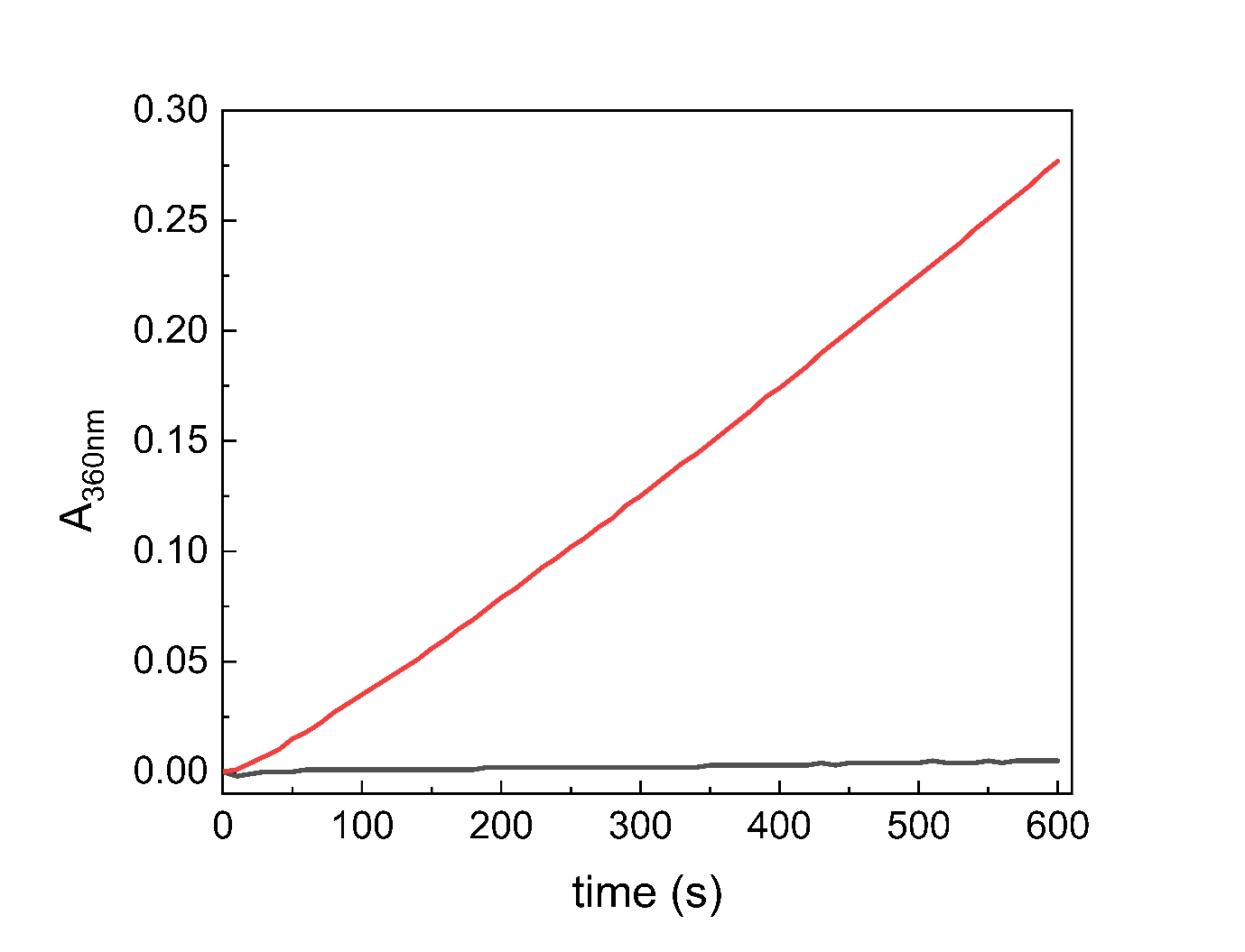


Supplementary Figure 6. Enzymatic activity of wild-type OaPAC in dark and light conditions. Kinetics of ATP conversion in the dark (black line) and in the light (red line) showing that the ATP conversion in the dark is negligible.

- **Supplementary Note 7: Comparison of the experimental SAXS data and SAXS-derived models with the OaPAC crystal structure**

i


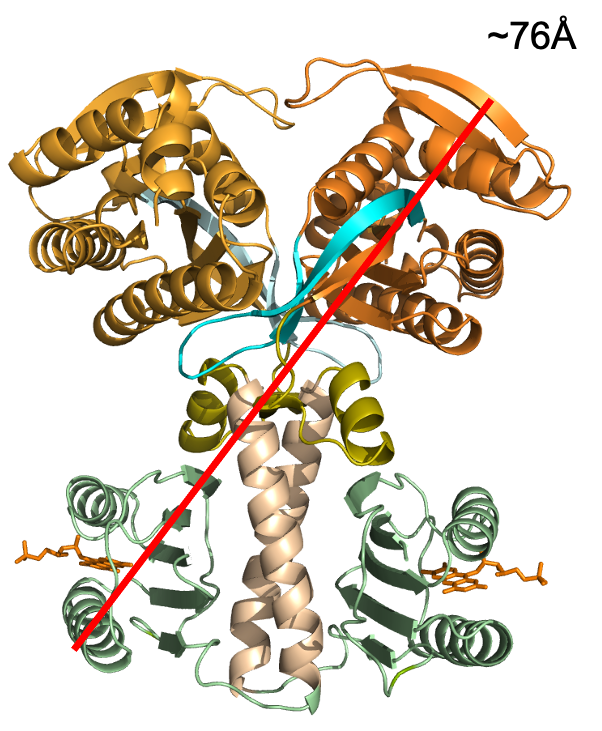


ii


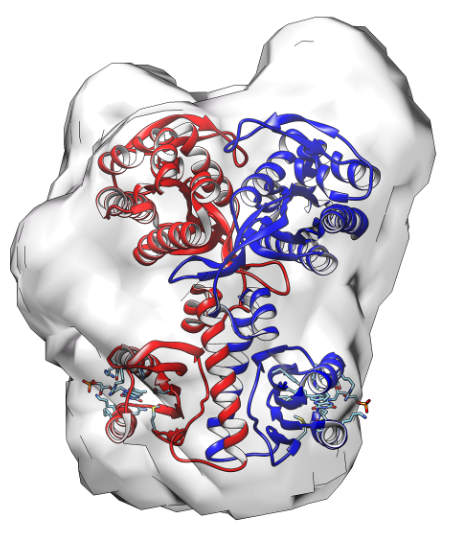


iii


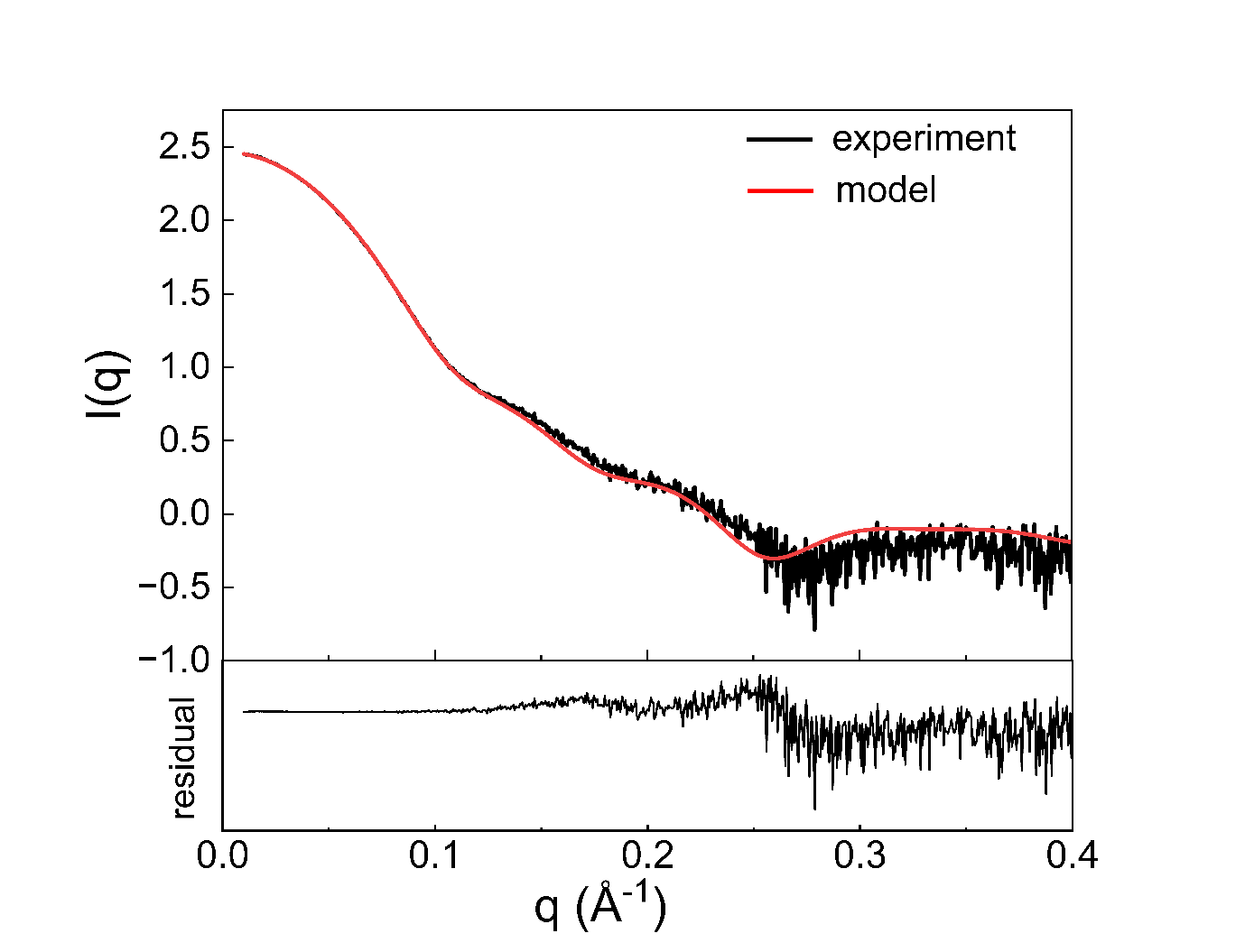


Supplementary Figure 7. i. Ribbon diagram of OaPAC showing an estimated maximum distance observed in the crystal form of the enzyme (pdb:4yut). ii. Superimposition of the *ab initio* electron density with the crystal structure of OaPAC described in the text. iii. Comparison of the experimental SEC-SAXS profile of OaPAC (black line) with the theoretical one derived from the crystal structure (pdb:4yut) (red line) using CRYSOL (χ^2^=3.486).

- **Supplementary Note 8: SAXS curves of OaPAC and its complex with ATP and theoretical curves of bPAC-Y7F and OaPAC and their complexes with an ATP analogue and ATP, respectively.**


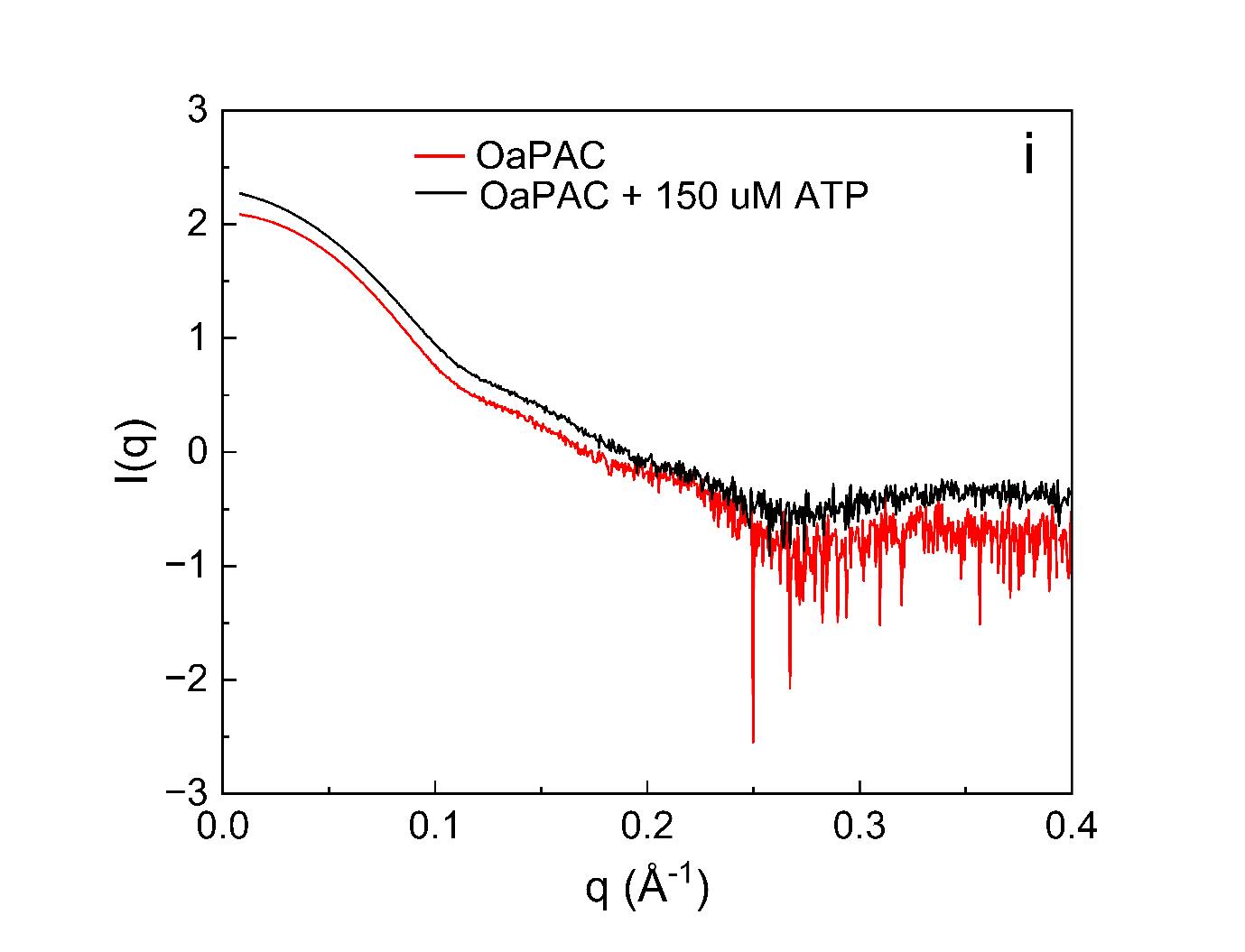


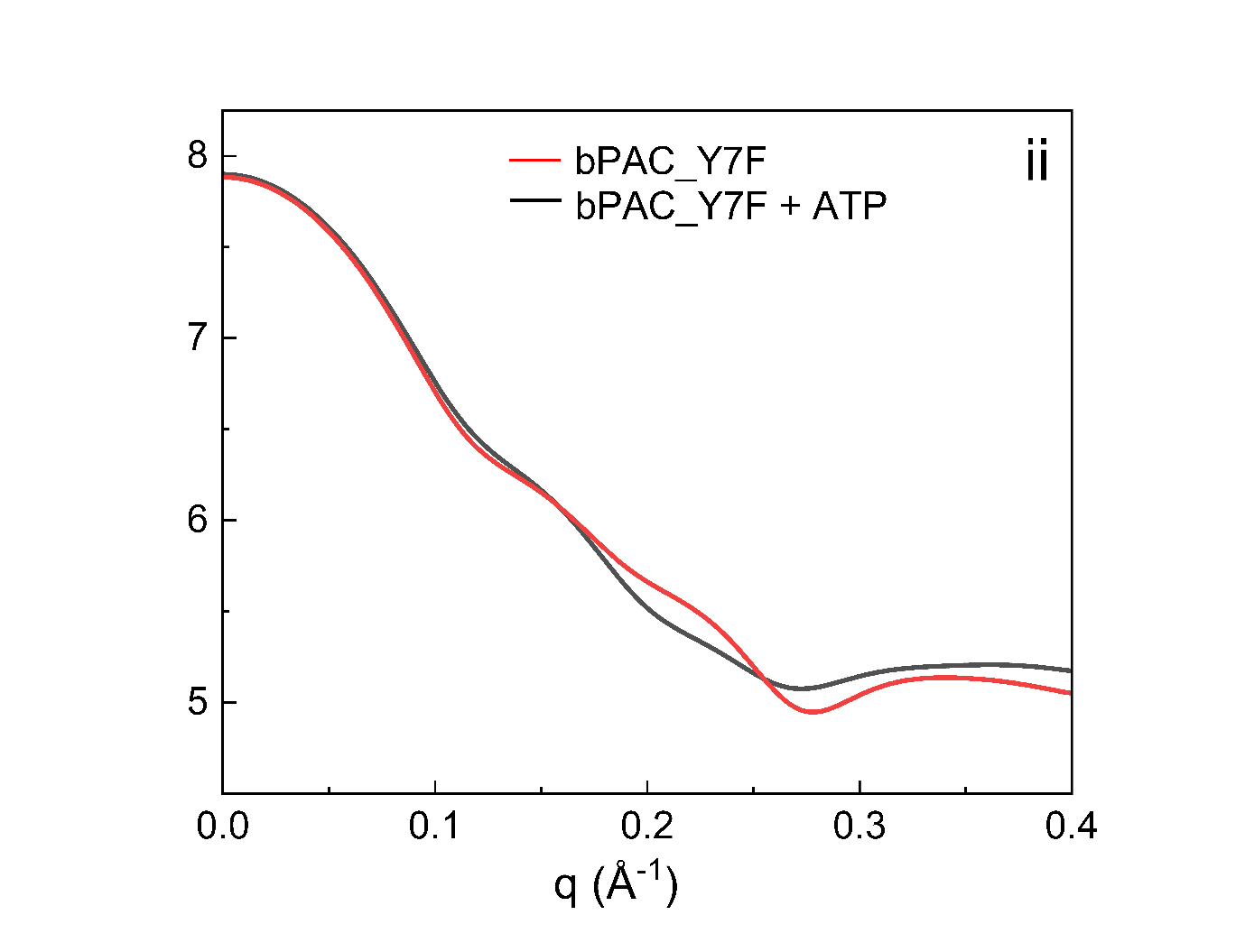


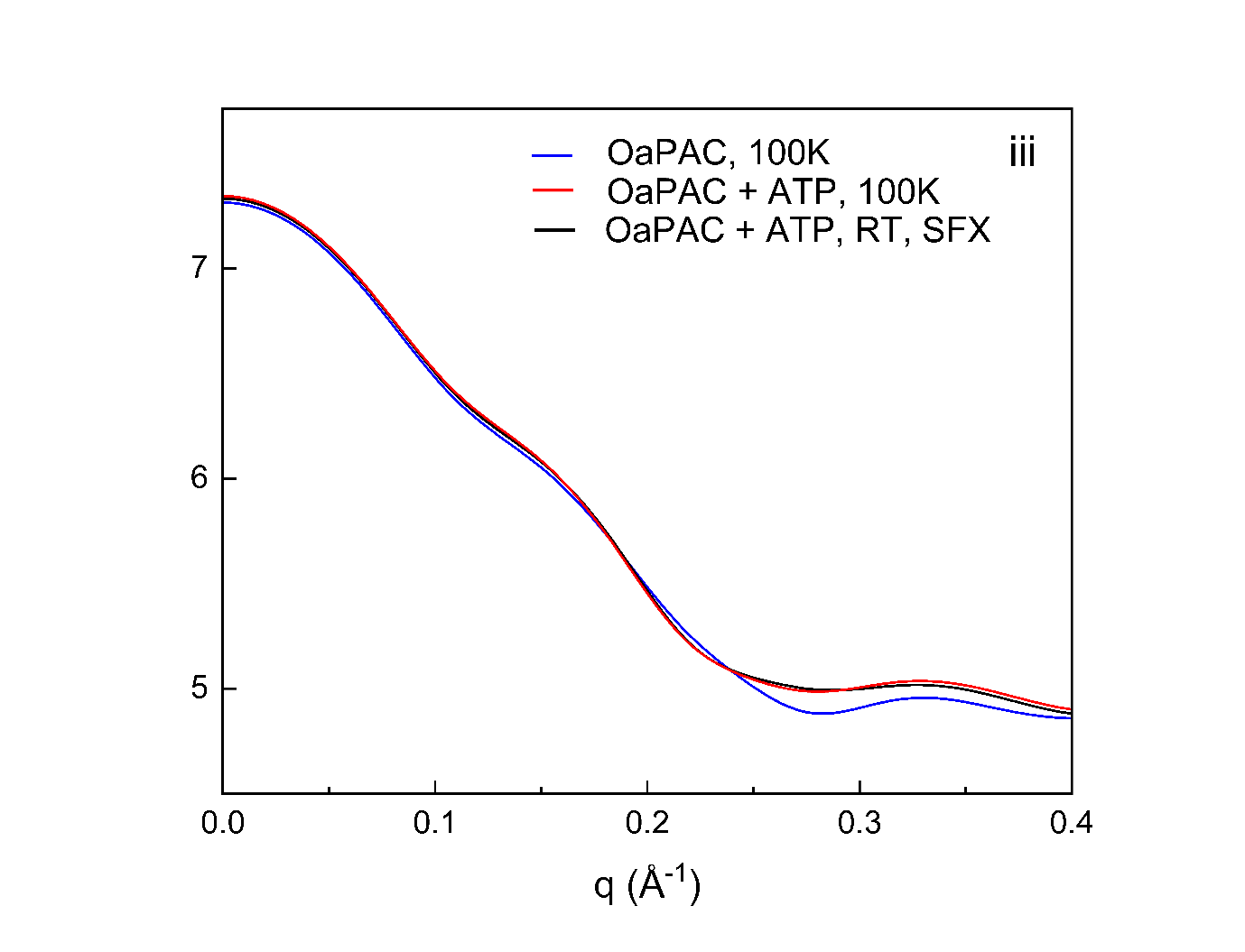


Supplementary Figure 8. i). SAXS data of OaPAC (red line) and its complex with ATP (black line). ii) Theoretical SAXS data of the Y7F mutant of bPAC (red line, pdb:5nby) and its complex with an ATP analogue (black line, pdb:5mbk). iii) Theoretical SAXS data of OaPAC at 100K (blue line, pdb:8qfe) and its complexes with ATP at 100K (red line, pdb:8qff) and room-temperature (black line, pdb:8qfh, SFX: serial femtosecond crystallography).

The following movies showing binding events of the buffer and single molecules of OaPAC are provided.

Supplementary Movie 1: Buffer molecules arriving at the cover slide of the MP set up.

Supplementary Movie 2: Single molecules of OaPAC arriving at the cover slide of the MP set up.
