# Supplementary material for: ATP-dependent conformational dynamics in a photoactivated adenylate cyclase revealed by fluorescence spectroscopy and small-angle X-ray scattering
Source: Commun Biol. 2024 Feb 2;7:147. doi: 10.1038/s42003-024-05842-1 (PMC10837130; doi:10.1038/s42003-024-05842-1)
Supplement: Supplementary file 2 — Description of Additional Supplementary Files [file 42003_2024_5842_MOESM2_ESM.pdf]

Description of Additional Supplementary Files

File name: Supplementary Movie 1
Description: Buffer molecules arriving at the cover slide of the MP set up.

File name: Supplementary Movie 2
Description: Potential single molecules of OaPAC arriving at the cover slide of the MP set up.
